# Blockade of T Cell Contact-Activation of Human Monocytes by High-Density Lipoproteins Reveals a New Pattern of Cytokine and Inflammatory Genes

**DOI:** 10.1371/journal.pone.0009418

**Published:** 2010-02-25

**Authors:** Lyssia Gruaz, Céline Delucinge-Vivier, Patrick Descombes, Jean-Michel Dayer, Danielle Burger

**Affiliations:** 1 Division of Immunology and Allergy, Inflammation and Allergy Research Group, Hans Wilsdorf Laboratory, Department of Internal Medicine and Faculty of Medicine, University of Geneva, Geneva, Switzerland; 2 Genomics Platform, National Center of Competence in Research ‘Frontiers in Genetics’, University of Geneva, Geneva, Switzerland; 3 Faculty of Medicine, University of Geneva, Geneva, Switzerland; Fundação Oswaldo Cruz, Brazil

## Abstract

**Background:**

Cellular contact with stimulated T cells is a potent inducer of cytokine production in human monocytes and is likely to play a substantial part in chronic/sterile inflammatory diseases. High-density lipoproteins (HDL) specifically inhibit the production of pro-inflammatory cytokines induced by T cell contact.

**Methodology/Principal Findings:**

To further elucidate the pro-inflammatory functions of cellular contact with stimulated T cells and its inhibition by HDL, we carried out multiplex and microarray analyses. Multiplex analysis of monocyte supernatant revealed that 12 out of 27 cytokines were induced upon contact with stimulated T cells, which cytokines included IL-1Ra, G-CSF, GM-CSF, IFNγ, CCL2, CCL5, TNF, IL-1β, IL-6, IL-8, CCL3, and CCL4, but only the latter six were inhibited by HDL. Microarray analysis showed that 437 out of 54,675 probe sets were enhanced in monocytes activated by contact with stimulated T cells, 164 probe sets (*i.e.*, 38%) being inhibited by HDL. These results were validated by qPCR. Interestingly, the cytokines induced by T cell contact in monocytes comprised IL-1β, IL-6 but not IL-12, suggesting that this mechanism might favor Th17 polarization, which emphasizes the relevance of this mechanism to chronic inflammatory diseases and highlights the contrast with acute inflammatory conditions that usually involve lipopolysaccharides (LPS). In addition, the expression of miR-155 and production of prostaglandin E_2_—both involved in inflammatory response—were triggered by T cell contact and inhibited in the presence of HDL.

**Conclusions/Significance:**

These results leave no doubt as to the pro-inflammatory nature of T cell contact-activation of human monocytes and the anti-inflammatory functions of HDL.

## Introduction

Imbalance in cytokine homeostasis is thought to play an important part in the pathogenesis of chronic inflammatory diseases such as rheumatoid arthritis and multiple sclerosis. This suggests that the mechanisms underlying the production of pro-inflammatory cytokines and their natural inhibitors fail to undergo natural regulation. In chronic/sterile immuno-inflammatory diseases, in the absence of an infectious agent, the factors triggering pro-inflammatory cytokine production are still elusive. We demonstrated that T cells may exert a pathological effect through direct cellular contact with monocytes/macrophages, inducing massive up-regulation of interleukin-1β (IL-1β) and tumor-necrosis factor (TNF) [1]–[3]. Indeed, the levels of T cell contact-induced production of IL-1β and TNF are comparable to those observed with optimal doses of lipopolysaccharides (LPS), a stimulus related to acute/infectious inflammation [2]. Besides triggering pro-inflammatory cytokine production, contact-mediated activation of monocytes induces the production and/or shedding of cytokine inhibitors such as secreted IL-1 receptor antagonist (sIL-1Ra) and soluble receptors of IL-1 and TNF [4]–[7]. Since T cells isolated from inflamed rheumatoid arthritis synovium constitutively display the ability to induce cytokine production in human monocytes in vitro [8], this mechanism is likely to occur also in vivo and thus to be involved in the pathogenesis of chronic inflammatory disorders leading to tissue destruction. The importance of T cell contact-mediated activation of monocytes/macrophages in chronic inflammatory conditions was further borne out by the modulation occurring after drugs were administered to patients for treatment. Indeed, interferon β (IFNβ) and glatiramer acetate, i.e., both immunomodulators used to treat multiple sclerosis, modulate the production of IL-1β and sIL-1Ra in T cell contact-activated monocytes in a manner that correlates with measurements in treated patients [9], [10]. This also applies to leflunomide which is used to treat rheumatoid arthritis patients [11].

In chronic inflammation, after extravasation, most T cells remain in the perivascular region, and other infiltrating cells such as monocytes/macrophages and neutrophils have to cross the perivascular layer of T cells to make contact with the latter cells before penetrating further into the target tissue. Consequently, direct cell-cell contact with T cells is less frequent outside perivascular regions. However, cells can disseminate cell surface molecules by generating microparticles and thus ensure “distant” cellular contact. Microparticles are fragments (0.1–0.8 µm diameter) shed from the plasma membrane of stimulated or apoptotic cells. We recently determined that microparticles generated by stimulated T cells display an ability to activate monocytes that is similar to that displayed by membranes of stimulated T cells or soluble extracts of the latter [12]. High-density lipoproteins (HDL) specifically inhibit the induction of IL-1β and TNF in monocytes activated by cellular contact with stimulated T cells [13]. This anti-inflammatory function of HDL proved to be due to their main protein component, apolipoprotein A–I (apo A–I), which binds activating factors at the surface of stimulated T cells [13]. In vivo, apo A–I infiltration into inflamed rheumatoid arthritis joints might lower the production of pro-inflammatory cytokines [14]. Of note, contrary to the production of IL-1β and TNF, that of the anti-inflammatory cytokine sIL-1Ra induced by contact with stimulated T cells is not affected by HDL [12]. It follows that stimulated T cells activate different monocyte functions by expressing surface molecules. The activity of the latter may be inhibited or not by HDL.

This study was undertaken to identify molecules that are induced in monocytes by cellular contact with stimulated T cells and inhibited in the presence of HDL. The experimental approach involved multiplex measurement of cytokines, chemokines and growth factors produced by activated monocytes, as well as microarray profiling. Studies of cell-cell interactions such as those occurring in T cell contact-activation of human monocytes are usually complicated by the simultaneous presence of at least two viable cell types. To obviate this problem and possible interferences due to potentially phagocytic target cells, isolated membranes from stimulated HUT-78 cells were solubilized and used as a stimulus referred to as CE_sHUT_ [9]. HUT-78 cells have previously been shown to activate monocytes to a similar extent as freshly isolated T lymphocytes [2]. The results show that direct contact with stimulated T cells induces the expression of genes mostly related to inflammatory pathways but different from those induced under acute/infectious inflammatory conditions (*e.g.*, induced by LPS), and that HDL inhibit the expression of pro- rather than anti-inflammatory molecules.

## Results

### Apolipoprotein A–I Is the HDL Component Displaying Inhibitory Activity

**Figure 1:**
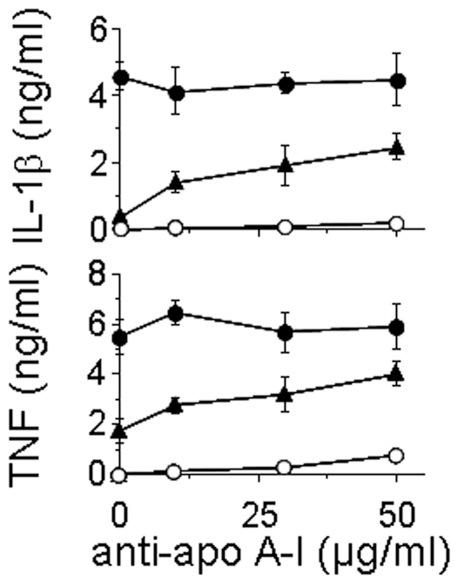
Apo A–I mediates the inhibitory effects of HDL in T cell contact-induced cytokine production in monocytes. Monocytes (5×10^4^ cells/200 µl/well; 96-well plates) were activated (closed symbols) or not (open circles) by CE_sHUT_ for 24 h in the presence (triangles) or absence (circles) of HDL. Cell culture supernatants were tested by Quantikine kits (R&D) for the presence of IL-1β and TNF. Results are expressed as mean±SD of a representative experiment carried out in triplicate.

We previously demonstrated that apolipoprotein A–I was the HDL component that inhibited T cell contact-activation of monocytes [13]. Since HDL preparations may contain several particle subpopulations, we first ascertained that the inhibitory activity of the HDL preparation used in this study was due to apo A–I. As shown in Figure 1, the inhibitory activity of HDL was reversed in a dose-dependent manner by antibodies to apo A–I. Antibodies to apo A–I alone did not affect IL-1β and TNF production by human monocytes. Similarly, they did not change CE_sHUT_-induced production of IL-1β or TNF in the absence of HDL. Isotype IgG used as control did not display any effect (not shown). This further confirms that apo A–I was indeed the inhibitory component of HDL.

### HDL Inhibit CE_sHUT_-Induced Inflammatory Cytokine Production in Human Monocytes

**Figure 2:**
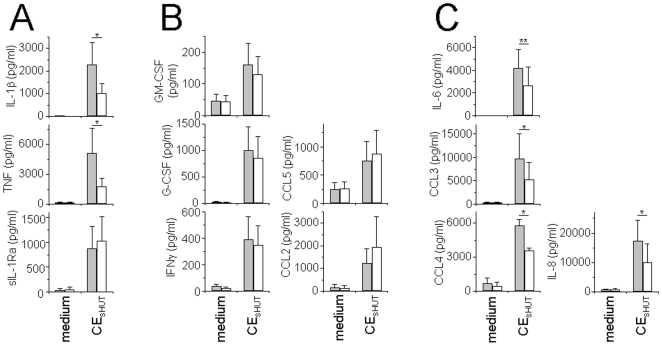
Modulation of cytokine production by HDL in CE_sHUT_-activated monocytes. Monocytes (5×10^4^ cells/200 µl/well; 96-well plates) were activated by CE_sHUT_ for 24 h in the presence (empty columns) or absence (grey columns) of HDL. Cell culture supernatants were tested for the presence of the indicated cytokines by BioPlex using a 27-Plex kit. Results were obtained from monocytes isolated from 3 individual donors and are expressed as mean ± SD (n = 3). (A) Cytokines previously shown to be modulated by CE_sHUT_; (B) CE_sHUT_-induced cytokines whose expression was not modulated by HDL; and (C) CE_sHUT_-induced cytokines whose expression was inhibited in the presence of HDL.

To ascertain the anti-inflammatory functions of HDL in chronic inflammatory conditions, human monocytes were activated by CE_sHUT_ in the presence or absence of HDL. Culture supernatants were analyzed for the production of cytokines, chemokines and growth factors using a 27-Plex kit (BioRad). Fifteen factors out of 27 were not significantly induced by CE_sHUT_, including cytokines specifically produced by T cells (*i.e.*, IL-2, IL-4, IL-5, IL-9, IL-13, and IL-17), cytokines and chemokines produced by a variety of cells (*i.e.*, IL-7, IL-10, IL-12, IL-15, CCL11 and CXCL10) and growth factors (*i.e.*, basic fibroblast growth factor – FGF, platelet-derived growth factor - PDGF, vascular endothelial growth factor - VEGF). As shown in Figure 2, the production of 12 out of 27 measured factors was induced by CE_sHUT_ including IL-1β, TNF and IL-1Ra. In contrast to sIL-1Ra production, that of IL-1β and TNF was inhibited by HDL (Figure 2A), corroborating previous results. In addition, CE_sHUT_ induced monocytes to produce factors involved in their localization, survival and differentiation such as CCL5 (RANTES), CCL2 (MCP-1), interferon-γ (IFNγ), granulocyte-macrophage colony-stimulating factor (GM-CSF), and macrophage-CSF (M-CSF). The production of the latter was moderate and it was not affected by HDL (Figure 2B). In contrast, CE_sHUT_ strongly induced the production of cytokines and chemokines that display robust pro-inflammatory functions, *i.e.*, IL-6, IL-8, CCL3 (MIP-1α) and CCL4 (MIP-1β), the latter being inhibited in the presence of HDL (Figure 2C). These data demonstrate that direct cellular contact with stimulated T cells (mimicked by CE_sHUT_) induced the production of many factors in addition to that of IL-1β, TNF and sIL-1Ra that had previously been studied. However, CE_sHUT_ was not a general activator of monocytes, it rather activated specific pathways relevant to inflammation. The premise that HDL mainly inhibited pro-inflammatory rather than anti-inflammatory factors strengthens the claim to their anti-inflammatory functions. Together, these results suggest that CE_sHUT_ triggered the expression of multiple factors in human monocytes, some of whose expression was modulated by HDL. This prompted us to conduct a microarray analysis to define genes whose expression was triggered by CE_sHUT_ and inhibited in the presence of HDL.

### Modulation of IL-1β Transcript Expression by CE_sHUT_ and HDL

**Figure 3:**
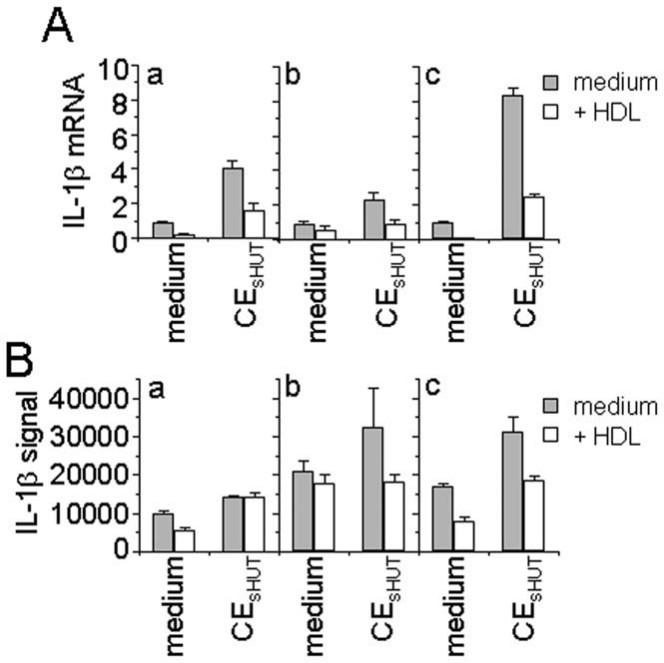
Inhibition by HDL of CE_sHUT_-triggered IL-1β mRNA. Monocytes (7×10^6^ cells/P-100mm/6 ml) isolated from 3 different donors (a, b and c) were activated by CE_sHUT_ (6 µg/ml) in the presence (empty columns) or absence (grey columns) of HDL (0.2 mg/ml) for 3 h. (A) Isolated RNA was subjected to qPCR to assess IL-1β transcript levels. Results are presented as mean ± SD of triplicates. (B) Isolated RNA was subjected to microarray analysis. Results are expressed as mean ± SD, n = 3.

**Figure 4:**
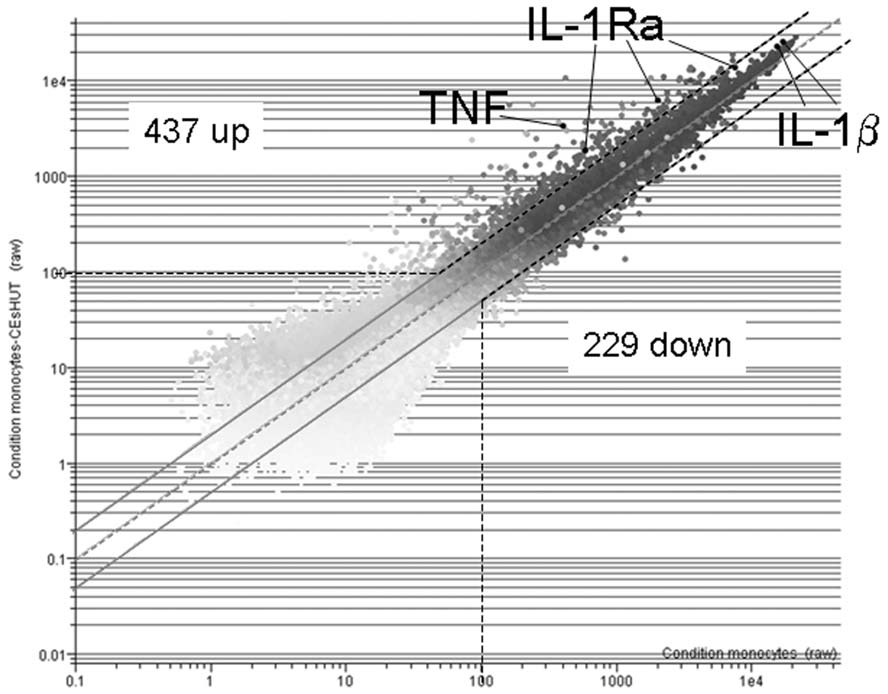
Analysis of gene expression in monocytes activated by CE_sHUT_. Total RNA was used in Affymetrix microarrays to analyse gene expression. The scatter plot (GeneSpring) shows the amounts expressed in CE_sHUT_-activated monocytes (y-axis) as a function of amounts expressed in resting monocytes (x-axis), applying a cut-off of 2.0. Each dot represents one probe set; probe sets for IL-1β, IL-1Ra and TNF are indicated. Probe sets with signals below 100 were discarded (dashed lines). Using the latter criteria, 437 probe sets were up-regulated, and 229 down-regulated as indicated.

Microarray analysis was carried out with monocytes prepared from 3 different human donors. The activation stage of monocytes was ascertained by measuring the levels of IL-1β mRNA whose modulations in the present settings are well known [12], [13]. As shown by qPCR, CE_sHUT_ induced the expression of IL-1β mRNA in the 3 monocyte preparations (Figure 3A). Although they differed depending on monocyte preparation, the IL-1β transcript levels were more than 2-fold higher in monocytes activated by CE_sHUT_ than in resting monocytes. In the presence of HDL, the CE_sHUT_-induced IL-1β transcript levels of donors a and b reverted to basal levels (Figure 3Aa and 3Ab) while those of donor c were inhibited by 83% Figure 3Ac). Although these data validated the experiments for microarray analysis, the induction of IL-1β transcript levels differed from that observed by qPCR (Figure 3B). Indeed, none of the two *IL1B* probe sets generated signal enhancement or inhibition by HDL that reached the 2-fold cut-off in any of the monocyte preparations, although the mean enhancement (1.55- and 1.48-fold) induced by CE_sHUT_ reverted completely in the presence of HDL. However, levels of the TNF transcript as determined by microarray analysis were similar to those previously obtained by qPCR [12], validating monocyte activation by CE_sHUT_ and inhibition by HDL in the 3 experiments. The discrepancy between qPCR and microarray measurements of IL-1β transcript levels was likely to be due to high signal values observed in resting monocytes for the 2 *IL1B* probe sets in microarray analysis (Figure 4). With such saturating signals, neither enhancement nor inhibition could be determined with accuracy. This was not true for TNF and two out of five IL-1Ra probe sets that were in the range where signal variations were optimal (Figure 4).

### Comparison of Microarray Analysis of Gene Expression with Protein Production

In order to corroborate results from measuring protein production and gene expression, results shown in Figure 2 were compared to those of microarray analysis. There was no enhancement of gene expression for cytokines, chemokines and growth factors that were not detected in supernatants of CE_sHUT_-activated monocytes (*i.e.*, IL-7, IL-10, IL-12, IL-15, CCL11, FGF, and VEGF) with the exception of PDGF-β and CXCL10. Indeed, the signals of *PDGFB* and *CXCL10* probe sets were enhanced respectively by factors of 2.88±0.26 and 20.09±21.42 in monocytes activated by CE_sHUT_ (Table S1), whereas proteins did not reach the detection limit in cell supernatants. This discrepancy could not be explained for CXCL10; however, the premise that PDGF is a heterodimer of PDGF-α and -β and *PDGFA* transcript remained undetectable in both resting and activated monocytes, might explain that PDGF was not detected in monocyte supernatants. In contrast, signals of probe sets for CCL5, G-CSF (*CSF3*) and GM-CSF (*CSF2*) that were measured in supernatants of CE_sHUT_-activated monocytes were not increased. This might be due to the time-course of the latter gene expression, the transcript being measured after 3 h of monocyte activation and that of secreted proteins after 24 h. Thus, the transcription of these genes might be delayed. Interestingly, factors displaying anti-inflammatory functions such as IL-10 and TGFβ were not induced, the signal of the *TGFB* probe set being even diminished 2.84-fold (Table S1). The probe set of IL-23 α-subunit (*IL23A*) was enhanced 18.24-fold, whereas IL-23 was not detectable in supernatants of CE_sHUT_-activated monocytes. This was not due to an incapacity of monocytes to produce IL-23, since the same monocyte preparations yielded 781±572 pg/ml IL-23 (mean ± SD, n = 3) when activated by 100 ng/ml LPS (not shown). Interestingly, probe sets for IL-12 α- and β-subunits were not detected in either resting or CE_sHUT_-activated monocytes, further suggesting that the active form of IL-23 which is composed of a dimer of IL-23 α-subunit and IL-12 β-subunit was not produced by CE_sHUT_-activated monocytes. Together, these results corroborate those obtained by measuring protein secretion in CE_sHUT_-activated monocyte supernatants (Figure 2).

### HDL Mainly Affect the Expression of Molecules Related to Inflammation in CE_sHUT_-Activated Monocytes

**Figure 5:**
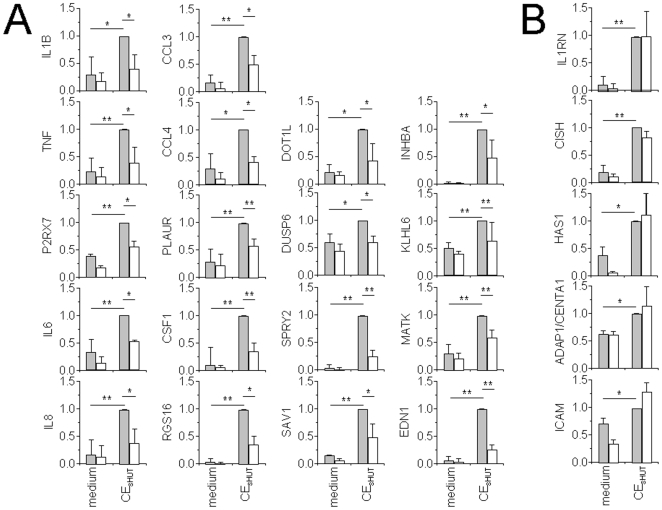
qPCR assessment of selected transcripts to validate microarray results. Several genes listed in Table S4 were selected and RNA, isolated from the 3 monocyte preparations, was subjected to qPCR. (A) Genes that were validated (*i.e.*, those displaying similar behavior as in microarray analysis). (B) Genes that were not validated. Results were normalized to fully activated condition (CE_sHUT_), considered to be 1. Data are expressed as mean ± SD, n = 3; ** p<0.01; * p<0.05.

Microarray analysis was carried out with the following criteria: 1) since signals differ greatly as a function of monocyte preparation we proceed to paired comparisons within experiment carried out with the same monocyte preparation; 2) probe sets with signals <100 in the CE_sHUT_-activated condition were discarded considered as not different from background; and 3) only probe sets with fold changes ≤−2.0 or ≥2.0 in at least 2 out of the 3 experiments were considered as affected by CE_sHUT_. By using this strategy, 666 probe sets were identified that were either enhanced (437, *i.e.*, 66%) or decreased (229, *i.e.*, 34%) upon monocyte activation by CE_sHUT_ (Figure 4; complete list in Table S1). In the presence of HDL, 1101 probe sets were modulated, 48% (524 probe sets) being increased and 52% (577 probe sets) decreased in [CE_sHUT_ + HDL]-activated monocytes (complete list in Table S2). The premise that genes modulated by CE_sHUT_ in the presence of HDL were more numerous than in the absence of HDL suggests that the expression of many genes was hampered upon T cell contact-activation of monocytes. Noticeably, 104 genes were significantly induced by CE_sHUT_ and not by [CE_sHUT_ + HDL], whereas 168 genes were increased by [CE_sHUT_ + HDL] and not by CE_sHUT_. Ingenuity Pathway Analysis (IPA) for genes whose expression was exclusive to one or the other condition demonstrate that genes induced by CE_sHUT_ were mainly related to inflammation whereas this correlation was poorer for genes uniquely induced by [CE_sHUT_ + HDL], both in terms of gene number and fitting to IPA defined pathways, *i.e.*, p-values (Table 1). HDL per se affected the expression of only 92 probe sets (39 increased, 53 decreased) in human monocytes (see complete list in Table S3). Alike *IL1B* some genes (probe sets) displayed high basal expression in resting monocytes (Figure 3 and 4). To take into account basal signals, we chose to compare signal ratios (fold changes) of “CE_sHUT_ versus medium” condition to “[CE_sHUT_ + HDL] versus medium” condition by pair, *i.e.*, fold changes in “CE_sHUT_ versus medium” condition were compared to fold changes in “[CE_sHUT_ + HDL] versus medium” condition in each experiment and the mean value was taken into account to determine the difference in probe set signals induced by CE_sHUT_ alone or by CE_sHUT_ in the presence of HDL. As presented in Table S4, 164 probe sets representing 113 molecules displayed ≥1.4-fold higher levels in [CE_sHUT_] than in [CE_sHUT_ + HDL] condition demonstrating that their induced expression was inhibited by HDL. To validate these results and our calculation strategy, genes were chosen from Table S4 and subjected to transcript level measurement by qPCR (Table 2). In addition, IL-1β and IL-8 were added to this list because they both displayed high basal signals in microarray analysis and their protein production was modulated by HDL (Figure 2 and Table 2). As shown in Figure 5, transcripts of the 23 chosen genes were all significantly increased in monocytes activated by CE_sHUT_ thus correlating microarray results. Eighteen out of 23 transcripts were inhibited in the presence of HDL. Noticeably, HDL per se diminished the levels of several transcripts in “resting” monocytes including CCL3 and CCL4 (Figure 5), thus correlating results of Table S3. However, the transcript expression of 5 genes, *i.e.*, *IL1RN*, *CISH*, *HAS1*, *ADAP1/CENTA1*, and *ICAM1* was not inhibited in the presence of HDL (Figure 5B). These genes displayed different expression patterns when measured by qPCR or by microarray analysis. No obvious reason could be determined for this discrepancy.

**Table 1:** IPA Pathways Associated with Genes whose Expression is Exclusive to [CE_sHUT_] or [CE_sHUT_ + HDL].

Condition	Diseases and Disorders	p-value*	number
[CE_sHUT_]	Inflammatory Response	5.2×10^−9^–2.85×10^−3^	25
	Inflammatory Diseases	1.66×10^−7^–2.82×10^−3^	32
[CE_sHUT_ + HDL]	Inflammatory Response	1.22×10^−4^–9.52×10^−3^	23
	Inflammatory Diseases	1.16×10^−3^–9.52×10^−3^	11

The 104 genes increased exclusively in monocytes activated by CE_sHUT_ ([CE_sHUT_]) and the 168 genes increased exclusively in monocytes activated by CE_sHUT_ in the presence of HDL ([CE_sHUT_ + HDL]) were subjected to IPA analysis. The table displays the number of genes related to inflammation. The significance is expressed as range of p values based on Fisher's exact test.

**Table 2:** Genes Chosen for Validation by qPCR.

Affymetrix ID	Gene Symbol	Description	Induction by CE_sHUT_ (fold change)	Inhibition by HDL (fold change)
90265_at	ADAP1/CENTA1	Arf-GAP with dual PH domain-containing protein 1 (centaurin, α 1)	2.30	−2.23
205114_s_at	CCL3	Chemokine (C-C motif) ligand 3	3.89	−1.55
204103_at	CCL4	Chemokine (C-C motif) ligand 4	2.64	−2.00
223961_s_at	CISH	Cytokine inducible SH2-containing protein (CIS)	4.91	−2.29
209716_at	CSF1	Macrophage-colony stimulating factor 1 (M-CSF)	10.86	−5.19
226201_at	DOT1L	Histone-lysine N-methyltransferase, H3 lysine-79 specific	2.17	−2.90
208893_s_at	DUSP6	Dual specificity phosphatase 6	3.78	−2.21
222802_at	EDN1	Endothelin 1	5.03	−3.80
207316_at	HAS1	Hyaluronan synthase 1	3.52	−1.95
202638_s_at	ICAM1	Intracellular adhesion molecule 1 (CD54)	2.22	−1.90
212659_s_at	IL1RN	Interleukin-1 receptor antagonist	3.69	−4.30
205207_at	IL6	Interleukin-6	3.18	−1.70
204926_at	INHBA	Inhibin, beta A (activin A, activin AB alpha polypeptide)	8.38	−2.17
1560397_s_at	KLHL6	Kelch-like 6	2.31	−2.63
206267_s_at	MATK	Megakaryocyte-associated tyrosine kinase	3.60	−3.09
207091_at	P2RX7	P2X purinoceptor 7 (ATP receptor)	2.77	−1.53
210845_s_at	PLAUR	Urokinase plasminogen activator surface receptor	2.57	−1.41
209325_s_at	RGS16	Regulator of G-protein signaling 16	11.15	−4.36
238729_x_at	SAV1	Protein salvador homolog 1	10.18	−2.26
204011_at	SPRY2	Protein sprouty homolog 2	24.82	−2.02
207113_s_at	TNF	Tumor necrosis factor α	7.85	−2.06
205067_at	IL1B	Interleukin 1β	1.55	−1.42
202859_x_at	IL8	Interleukin 8	1.30	−1.31

### HDL Inhibit the CE_sHUT_-Induced Expression and Secretion of Non-Protein Compounds

**Figure 6:**
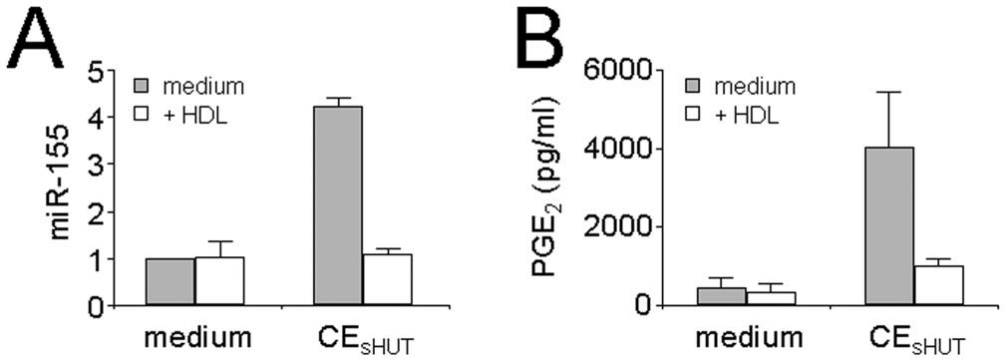
HDL inhibit miR-155 expression and PGE_2_ production induced by CE_sHUT_ in human monocytes. (A) Total RNA isolated from monocytes activated (CE_sHUT_) or not (medium) in the presence (white columns) or absence (grey columns) of HDL and subjected to qPCR analysis for the presence of miR-155. (B) Monocytes were activated as in Figure 2, and PGE_2_ production measured in cell supernatants. Results are expressed as mean ± SD of 3 different experiments.

CE_sHUT_ not only induced the transcription of genes encoding proteins but also that of non-protein-coding genes such as *MIRHG2/BIC* which is inhibited in the presence of HDL (Table S4). *MIRHG2/BIC* is the microRNA host gene 2 which contains miR-155. In acute inflammation, miR-155 expression is induced by LPS and participates in the up-regulation of IL-1β and TNF in LPS-activated dendritic cells [15]. Since miR-155 plays an important part in inflammatory processes, its induction by CE_sHUT_ and inhibition by HDL were assessed by qPCR. As illustrated in Figure 6A, miR-155 expression was indeed induced by CE_sHUT_ and inhibited in the presence of HDL, thus validating the microarray results (Table S4). Furthermore, CE_sHUT_ induced the expression of enzymes involved in the synthesis of non-protein pro-inflammatory compounds such as eicosanoids. To determine whether the induction of cyclooxygenase-2 (COX-2) transcript by CE_sHUT_ and its inhibition by HDL (Table S4) were associated with the expression of a functional enzyme, we tested monocyte supernatants for the production of its product, *i.e.*, prostaglandin E_2_ (PGE_2_). As shown in Figure 6B, CE_sHUT_-activated monocytes did indeed produce PGE_2_, which production was inhibited in the presence of HDL. Together, the results presented in Figure 6 further point to contact with stimulated T cells as a pro-inflammatory stimulus of monocytes that does not only induce cytokines but also non-protein components such as microRNAs and prostaglandins related to inflammation.

### HDL Inhibited the Expression of Genes Involved in Inflammatory Processes

**Figure 7:**
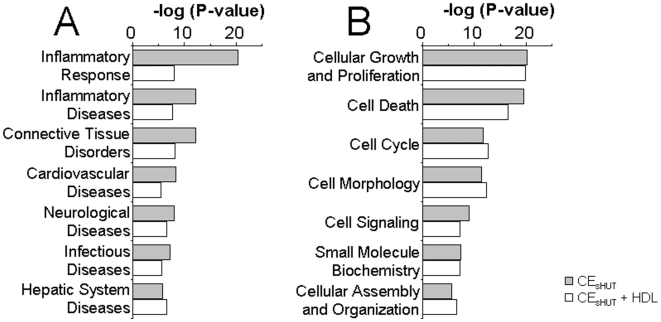
HDL inhibit inflammatory functions induced by CE_sHUT_ in human monocytes. Microarray results of [CE_sHUT_] versus medium (grey columns) and [CE_sHUT_ + HDL] versus medium (empty columns) were subjected to comparative analysis in the IPA system. (A) Genes related to “Diseases and Disorders”; (B) genes related to “Molecular and Cellular Functions”.

In order to establish that HDL mainly affected inflammatory functions, we run an IPA comparing the following conditions: CE_sHUT_ versus medium (666 probe sets) and [CE_sHUT_+HDL] versus medium (1101 probe sets, Table S2). The analysis revealed that among the biofunctions that were modulated by cellular contact with stimulated T cells, those that were inhibited by HDL related mainly to inflammation and immunity (Figure 7). Of the genes associated with “Diseases and Disorders” (according to IPA), those correlated to inflammatory response and inflammatory diseases in CE_sHUT_ (p-values between 5.25×10^−20^ and 1.28×10^−5^) were a better match than those in [CE_sHUT_ + HDL] (p-values between 7.52×10^−9^ and 6.42×10^−5^). Of note, genes related to rheumatoid arthritis, atherosclerosis and multiple sclerosis were also attributed by IPA to “Connective Tissue Disorders”, “Cardiovascular Disease”, and “Neurological Disease”, respectively. These genes proved to be a better match in the [CE_sHUT_] conditions than in the [CE_sHUT_ + HDL] conditions. This is further evidence of HDL down-modulating inflammation-related genes in monocytes activated by CE_sHUT_. Genes implicated in cell survival and metabolism, including those related to “cell growth and proliferation”, “cell death”, and “cell cycle”, were only modulated to a small extent or not at all in the presence of HDL (Figure 7B). These results suggest that, in human monocytes, CE_sHUT_ mainly affected the expression of molecules related to inflammation and that their expression was inhibited in the presence of HDL. IPA of probe sets, whose signals were increased by CE_sHUT_ and decreased by HDL (Table S4), revealed that genes whose CE_sHUT_-induced expression was inhibited by HDL were mainly involved in inflammatory diseases and disorders (Table 3). The global outcome of the IPA of microarray results suggests that CE_sHUT_ mainly affected inflammatory and immune functions in human monocytes and that HDL opposed these functions due to its inhibitory effect.

**Table 3:** IPA Pathways Associated with Genes whose CE_sHUT_-Induced Expression is Inhibited by HDL.

Diseases and Disorders	p-value	number
Cancer	9.19×10^−17^–2×10^−6^	59
Inflammatory Diseases	2.51×10^−11^–2.35×10^−6^	41
Hematological Diseases	2.4×10^−11^–2.19×10^−6^	33
Inflammatory Response	1.97×10^−15^–2.35×10^−6^	32
Connective Tissue Disorders or Skeletal and Muscular Disorders	2.59×10^−10^–1.91×10^−6^	29
Reproductive System Diseases	1.06×10^−10^–1.87×10^−6^	28
Neurological Diseases	1.67×10^−9^–1.53×10^−6^	27
Immunological Diseases	2.4×10^−11^–2.19×10^−6^	25
Genetic Disorders	2.59×10^−10^–2.35×10^−6^	25
Dermatological Diseases and Conditions	8.58×10^−12^–1.11×10^−6^	21
Cardiovascular Diseases	4.41×10^−10^–1.94×10^−6^	20

## Discussion

This study demonstrates that activation of monocytes by cellular contact with stimulated T cells as mimicked by CE_sHUT_ is a pro-inflammatory mechanism distinct from that induced by LPS. In addition to the prototypical pro-inflammatory cytokines IL-1β and TNF, CE_sHUT_ induces the expression of many genes related to inflammatory processes, thus generating a microenvironment which may contribute to TH17 polarization. HDL does not randomly inhibit T cell contact-activated monocytes but specifically inhibit CE_sHUT_-induced pro-inflammatory genes. In contrast, HDL do not affect or inhibit - or only to a small extent - the expression of molecules displaying anti-inflammatory and regulatory functions.

According to Multiplex analysis of soluble factors induced by CE_sHUT_, of the important pro-inflammatory factors, IL-12 is not produced by activated human monocytes. This is substantiated by microarray analysis which failed to reveal transcripts for IL-12p40 (*IL12B*) or IL-12p35 (*IL12A*) in both resting and CE_sHUT_-activated monocytes. This suggests that T cell contact-mediated activation of monocytes does not generate a cytokine environment propitious to Th1 polarization [16]. IL-1β and IL-6 however, which are involved in Th17 differentiation [17], are highly induced by CE_sHUT_ in monocytes (Figure 2), suggesting that contact with stimulated T cells might result in a bias towards pro-inflammatory Th17 cells [18]. This is particularly important in view of recent results showing that Th17 rather than Th1 cells display pathogenic functions in chronic inflammatory diseases such as multiple sclerosis and rheumatoid arthritis [19]. Interestingly, LPS, *i.e.*, the prototypical stimulus of acute inflammation, induces IL-12 in human monocytes [20], thus distinguishing the latter inflammatory mechanism from chronic/sterile inflammation which implicates contact with stimulated T cells. Together these observations suggest that T cell contact-activated monocytes might generate a cytokine microenvironment inclined to give rise to a bias towards Th17 at the inflammatory site.

IL-10, whose production is induced upon human monocyte activation by LPS [21], was not detected in CE_sHUT_-activated monocyte supernatants, nor was its transcript detected (*i.e.*, signal <100) in microarray analysis. These results are reminiscent of previous data showing that IL-10 mRNA was not expressed in monocytes activated by membranes of stimulated T cells for 1 or 24 h [22]. Similarly, cytokine-stimulated T lymphocytes, which are comparable to rheumatoid arthritis synovium T lymphocytes [8], fail to trigger IL-10 production in monocytes [23], but not in M-CSF-differentiated macrophages [24]. Together these observations suggest that T cell contact-induced IL-10 production depends on the differentiation stage of monocytes/macrophages. Since IL-10 is involved in the reduction and resolution of inflammation [25], its lack of production upon T cell contact-activation of monocytes might explain in part the persistence of inflammation in chronic, destructive inflammatory diseases.

The microarray analysis of CE_sHUT_-activated monocytes also identified the up-regulation (4.75-fold) of the *MIRHG2/BIC* probe set. *MIRHG2/BIC* is the host gene of miR-155. The expression of miR-155 was validated by qPCR (Figure 6), demonstrating the enhancement of miR-155 expression (4.22±0.2-fold) upon CE_sHUT_-activation of human monocytes and its inhibition to basal levels in the presence of HDL. MicroRNAs have emerged as a major class of gene expression regulators linked to most biological functions. MiR-155 is up-regulated by LPS and other inflammatory mediators [26]. Recently, miR-155 has been identified as a regulator of the Toll-like receptor/interleukin-1 (TLR/IL-1) signalling pathway [15]. Of note, the production of IL-1β and TNF was lower in human miR-155 knock-down dendritic cells than in untransfected cells [15]. Thus, the premise that CE_sHUT_ induces the expression of miR-155 which is diminished in the presence of HDL (Tables S1 and S4) lends additional weight to its classification as a pro-inflammatory mechanism. Other genes involved in mRNA stability are induced upon CE_sHUT_-activation of monocytes; they include the exosome component RRP41 [27], whose probe set (*EXOSC4*) levels are inhibited in the presence of HDL.

In addition to cytokines, chemokines and growth factors (Figure 2) COX-2 transcript expression was enhanced 4.88-fold in monocytes activated by CE_sHUT_, suggesting that human monocytes activated by CE_sHUT_ may produce eicosanoids. CE_sHUT_ induced the production of PGE_2_ in monocytes, which was inhibited by HDL (Figure 6). This is in keeping with the premise that COX-2 expression was inhibited in the presence of HDL (Table S4). Together, these data suggest that HDL may inhibit the CE_sHUT_-induced synthesis of eicosanoids, which constitutes an additional argument in favor of their anti-inflammatory action. Since PGE_2_ is also involved in Th17 polarization and maintenance [28], this result support the hypothesis that CE_sHUT_-activated monocytes may generate a microenvironment propitious to the generation of Th17 cells.

The production of IL-1β is tightly regulated at several levels by “roadblocks” as defined by Dinarello [29]. The latter include the facilitation of IL-1β processing by the caspase-1 inflammasome through ATP activation of the P2X_7_ receptor. As shown in Table S4, the transcription of genes whose proteins are involved in IL-1β processing and secretion was induced by CE_sHUT_ and inhibited in the presence of HDL. Indeed, *CIAS1/NLRP3* and *P2RX7* genes that code for NALP-3 and P2X_7_ receptor - both involved in the IL-1β secretion process [30] - are induced 3.01- and 2.77-fold, respectively, upon CE_sHUT_-activation, and inhibited in the presence of HDL (Tables S1 and S4). This demonstrates that in addition to inducing the IL-1β transcript, cellular contact with stimulated T cells triggers processes that underlie optimal/detrimental production of IL-1β upon chronic/sterile inflammatory conditions.

The present microarray results were obtained from monocytes activated by CE_sHUT_ for 3 h. Although genes might be up- or down-modulated at other time-points, the results of multiplex and ELISA validated most of results seen with cytokine transcripts. Modulation of highly expressed genes (i.e., displaying saturating, basal signals higher than 10,000) like IL-1β and IL-8 might be similarly regulated but are outside the range of the present analysis. However, these consisted mainly of ribosomal protein transcripts. Of the transcripts that might be regulated by extracellular signals, only two were likely to be modulated by CE_sHUT_ and inhibited in the presence of HDL, i.e. *IER3* (immediate early response 3) and *FTH1* (ferritin, heavy polypeptide 1). We thus conclude that this analysis includes all genes of interest that are modulated by contact with stimulated T cells at 3 h.

In conclusion, the present results demonstrate that direct cellular contact with stimulated T cells induces pro-inflammatory factors and pathways in human monocytes and that HDL modulates these pathways to take on a less inflammatory pattern. CE_sHUT_-activation of monocytes induces conditions that may favor a bias toward Th17 in neighboring T cells. Interestingly, mechanisms able to induce a cytokine environment favoring such a bias, *i.e.*, inflammatory mechanisms which do not induce IL-12 production, have been poorly described although TLR2 ligands such as zymosan might favor IL-23 rather than IL-12 production [31]. This highlights the importance of this mechanism in chronic/sterile inflammatory diseases such as multiple sclerosis and rheumatoid arthritis, and in turn the role of its specific inhibition by HDL. Activation of human monocytes by contact with stimulated T cells might be a clue to the mechanisms underlying systemic chronic/sterile inflammatory conditions which remain poorly understood, but clearly do not seem to fit the classical pattern of transition from acute to chronic inflammation [32], [33].

## Materials and Methods

### Materials

Fetal calf serum (FCS), streptomycin, penicillin, L-glutamine, RPMI-1640, and PBS free of Ca^2+^ and Mg^2+^ (Gibco, Paisley, Scotland); purified phytohemagglutinin (PHA) (EY Laboratories, San Marco, CA); TriReagent, phorbol myristate acetate (PMA), polymyxin B sulfate (Sigma Chemicals Co., St. Louis, MO); and goat anti-apolipoprotein A–I antibodies and IgG (Academy Bio-medical Co.) were purchased from the designated suppliers. Other reagents were of analytical grade or better.

### Monocytes

Peripheral blood monocytes were isolated from buffy coats of healthy blood donors as previously described [13]. In order to rule out activation by endotoxins, polymyxin B (2 µg/ml) was added to all solutions during the isolation procedure.

### T Cell Stimulation, and Plasma Membrane Isolation and Solubilization

The human T cell line HUT-78 was obtained from the ATCC (Rockville, MD). Cells were maintained in RPMI-1640 medium supplemented with 10% heat-inactivated FCS, 50 µg/ml streptomycin, 50 IU/ml penicillin and 2 mM L-glutamine in 5% CO_2_-air humidified atmosphere at 37°C. HUT-78 cells (2×10^6^ cells/ml) were stimulated for 6 h by PHA (1 µg/ml) and PMA (5 ng/ml). Plasma membranes of stimulated or unstimulated HUT-78 cells were prepared and solubilized in 8 mM CHAPS as previously described [9], [34]. CHAPS extracts of membranes from stimulated HUT-78 cells were referred to as CE_sHUT_ [12].

### Isolation of HDL

Human serum HDL were isolated as previously described [13]. In order to optimize the inhibitory effect of HDL, they were mixed with the stimulus prior to addition to monocytes.

### Cytokine Production

Monocytes (5×10^4^ cells/well/200 µl) were activated or not with 6 µg/ml CE_sHUT_ in RPMI 1640 medium supplemented with 10% heat-inactivated FCS, 50 µg/ml streptomycin, 50 U/ml penicillin, 2 mM L-glutamine and 5 µg/ml polymyxin B sulfate (medium) in 96-well plates and cultured for 24 h. The production of cytokines was measured in culture supernatants by Bio-Plex® analysis (Human Cytokine 27-Plex Panel, BioRad). Alternatively, some cytokines were determined by commercially available kits, Quantikine® from R&D.

### PGE_2_ Production

Monocytes (5×10^4^ cells/well/200 µl) were activated or not with 6 µg/ml CE_sHUT_ in RPMI 1640 medium supplemented with 10% heat-inactivated FCS, 50 µg/ml streptomycin, 50 U/ml penicillin, 2 mM L-glutamine and 5 µg/ml polymyxin B sulfate (medium) in 96-well plates and cultured for 24 h. The production of PGE_2_ was measured as described previously [35].

### Quantitative Real-Time PCR

Quantitative real-time duplex PCR analysis (qPCR) was conducted as previously described [36]. The mRNA expression levels were normalized with the expression of a housekeeping gene (18S) analyzed simultaneously. All probes were purchased from Applied Biosystems. Measurements were conducted in triplicates. miR-155 presence was ascertained by a commercially available kit (TaqMan MicroRNA assay, has-miR-155, Applied Biosystems) according to supplier's instructions.

### Microarray Analysis

Fourteen million of human monocytes (7×10^6^ cells/100 mm petri dish/6 ml) isolated from 3 normal blood donors were activated or not for 3 h by 6 µg/ml CE_sHUT_ in the presence or absence of 0.2 mg/ml HDL. When CE_sHUT_ was not added, its volume was compensated by PBS containing 8 mM CHAPS. Total RNA was isolated and processed using the RNeasy extraction kit (Qiagen), according to manufacturer's protocol. RNA was tested for integrity by capillary electrophoresis (2100 Bioanalyzer, Agilent) and used for the synthesis of biotin-labeled cRNA in accordance with the two-cycle target labeling protocol developed by Affymetrix (GeneChip® Eukaryotic Small Sample Target Labeling Assay Version II, Affymetrix). Biotin-labeled cRNA was hybridized to Affymetrix GeneChip® HG-U133 plus 2.0 containing 54,675 distinct probe sets. Three chips were hybridized and scanned, each corresponding to one donor, for each condition. Signal values and detection calls (present, marginal or absent) for each probe set were determined using the Affymetrix MAS5.0 algorithm. When required, the resulting lists of genes were subjected to analysis by Ingenuity Pathway Analysis (IPA, version 7.5, http://www.ingenuity.com/). IPA is a software application for associating genes that are part of a common regulatory biological network and thus for setting up, analyzing and understanding complex biological systems.

### Statistics

Where required, significance of differences between groups was assessed using Student's t test.

## Supporting Information

Table S1 Probe sets whose signal was modified in the presence of CE_sHUT_.
(1.33 MB DOC)

Table S2 Probe sets whose signal was affected in the presence of CE_sHUT_ and HDL.
(1.76 MB DOC)

Table S3 Probe sets whose signal was modulated by HDL.
(0.15 MB DOC)

Table S4 Probe sets whose signals was lower in monocytes activated by CE_sHUT_ + HDL than in monocytes activated by CE_sHUT_.
(0.29 MB DOC)
