# Supplementary material for: Blockade of T Cell Contact-Activation of Human Monocytes by High-Density Lipoproteins Reveals a New Pattern of Cytokine and Inflammatory Genes
Source: PLoS One. 2010 Feb 25;5(2):e9418. doi: 10.1371/journal.pone.0009418 (PMC2828473; doi:10.1371/journal.pone.0009418)
Supplement: Table S1 — Probe sets whose signal was modified in the presence of CEsHUT. (1.33 MB DOC) [file pone.0009418.s001.doc]

# Table S1: Probe sets whose signal was modified in the presence of CE_sHUT_

| **Affymetrix ID** | **Gene Symbol** | **Description** | **Representative ID** | **Fold Change** |
| --- | --- | --- | --- | --- |
| 238439_at | ANKRD22 | ankyrin repeat domain 22 | AI925518 | 72.22 |
| 230067_at | --- | Transcribed locus | AA151659 | 64.72 |
| 227140_at | INHBA | Inhibin, beta A (activin A, activin AB alpha polypeptide) | AI343467 | 41.17 |
| 204363_at | F3 | coagulation factor III (thromboplastin, tissue factor) | NM_001993 | 34.71 |
| 210511_s_at | INHBA | inhibin, beta A (activin A, activin AB alpha polypeptide) | M13436 | 27.56 |
| 204011_at | SPRY2 | sprouty homolog 2 (Drosophila) | NM_005842 | 24.82 |
| 221266_s_at | TM7SF4 | transmembrane 7 superfamily member 4 /// transmembrane 7 superfamily member 4 | NM_030788 | 24.50 |
| 204533_at | CXCL10 | chemokine (C-X-C motif) ligand 10 | NM_001565 | 20.09 |
| 209324_s_at | RGS16 | regulator of G-protein signalling 16 | BF304996 | 19.63 |
| 226498_at | FLT1 | Fms-related tyrosine kinase 1 (vascular endothelial growth factor/vascular permeability factor receptor) | AA149648 | 19.41 |
| 228186_s_at | RSPO3 | R-spondin 3 homolog (Xenopus laevis) | BF589322 | 19.01 |
| 219386_s_at | SLAMF8 | SLAM family member 8 | NM_020125 | 18.57 |
| 212946_at | KIAA0564 | KIAA0564 protein | AK025432 | 18.49 |
| 220054_at | IL23A | interleukin 23, alpha subunit p19 | NM_016584 | 18.24 |
| 239196_at | ANKRD22 | ankyrin repeat domain 22 | AI097229 | 16.44 |
| 204475_at | MMP1 | matrix metallopeptidase 1 (interstitial collagenase) | NM_002421 | 15.57 |
| 210517_s_at | AKAP12 | A kinase (PRKA) anchor protein (gravin) 12 | AB003476 | 14.85 |
| 229625_at | GBP5 | Guanylate binding protein 5 | BG545653 | 13.67 |
| 219385_at | SLAMF8 | SLAM family member 8 | NM_020125 | 13.65 |
| 226497_s_at | FLT1 | Fms-related tyrosine kinase 1 (vascular endothelial growth factor/vascular permeability factor receptor) | AA149648 | 13.04 |
| 227529_s_at | AKAP12 | A kinase (PRKA) anchor protein (gravin) 12 | BF511276 | 11.94 |
| 221085_at | TNFSF15 | tumor necrosis factor (ligand) superfamily, member 15 | NM_005118 | 11.84 |
| 229242_at | --- | Transcribed locus | BF439063 | 11.81 |
| 238581_at | GBP5 | Guanylate binding protein 5 | BG271923 | 11.16 |
| 209325_s_at | RGS16 | regulator of G-protein signalling 16 | U94829 | 11.15 |
| 209716_at | CSF1 | colony stimulating factor 1 (macrophage) | M37435 | 10.86 |
| 38037_at | HBEGF | heparin-binding EGF-like growth factor | M60278 | 10.68 |
| 236606_at | SAV1 | Salvador homolog 1 (Drosophila) | N50912 | 10.50 |
| 238729_x_at | SAV1 | Salvador homolog 1 (Drosophila) | BF983202 | 10.18 |
| 204824_at | ENDOG | endonuclease G | NM_004435 | 9.77 |
| 204411_at | KIF21B | kinesin family member 21B | NM_017596 | 9.65 |
| 213338_at | RIS1 | Ras-induced senescence 1 | BF062629 | 9.58 |
| 203889_at | SGNE1 | secretory granule, neuroendocrine protein 1 (7B2 protein) | NM_003020 | 9.09 |
| 235249_at | --- | MRNA; cDNA DKFZp313H0740 (from clone DKFZp313H0740) | AA736589 | 8.97 |
| 237204_at | DPH2L1 | Candidate tumor suppressor in ovarian cancer 2 | AI681671 | 8.49 |
| 213895_at | EMP1 | epithelial membrane protein 1 | BF445047 | 8.39 |
| 204926_at | INHBA | inhibin, beta A (activin A, activin AB alpha polypeptide) | NM_002192 | 8.38 |
| 206932_at | CH25H | cholesterol 25-hydroxylase | NM_003956 | 8.10 |
| 205249_at | EGR2 | early growth response 2 (Krox-20 homolog, Drosophila) | NM_000399 | 8.06 |
| 207113_s_at | TNF | tumor necrosis factor (TNF superfamily, member 2) | NM_000594 | 7.85 |
| 203821_at | HBEGF | heparin-binding EGF-like growth factor | NM_001945 | 7.83 |
| 227530_at | AKAP12 | A kinase (PRKA) anchor protein (gravin) 12 | BF511276 | 7.81 |
| 220882_at | --- | --- | NM_018612 | 7.74 |
| 231578_at | GBP1 /// LOC400759 | guanylate binding protein 1, interferon-inducible, 67kDa /// similar to Interferon-induced guanylate-binding protein 1 (GTP-binding protein 1) (Guanine nucleotide-binding protein 1) (HuGBP-1) | AW014593 | 7.35 |
| 39248_at | AQP3 | aquaporin 3 | N74607 | 7.15 |
| 223377_x_at | CISH | cytokine inducible SH2-containing protein | AF035947 | 7.05 |
| 236898_at | --- | Transcribed locus | AW242604 | 7.03 |
| 209969_s_at | STAT1 | signal transducer and activator of transcription 1, 91kDa | BC002704 | 7.01 |
| 215990_s_at | BCL6 | B-cell CLL/lymphoma 6 (zinc finger protein 51) | S67779 | 6.92 |
| 1559777_at | --- | CDNA FLJ32866 fis, clone TESTI2003718 | AK057428 | 6.80 |
| 209706_at | NKX3-1 | NK3 transcription factor related, locus 1 (Drosophila) | AF247704 | 6.78 |
| 218276_s_at | SAV1 | salvador homolog 1 (Drosophila) | NM_021818 | 6.72 |
| 203915_at | CXCL9 | chemokine (C-X-C motif) ligand 9 | NM_002416 | 6.67 |
| 235574_at | GBP4 | guanylate binding protein 4 | AW392952 | 6.51 |
| 226474_at | NOD27 | nucleotide-binding oligomerization domains 27 | AA005023 | 6.47 |
| AFFX-HUMISGF3A/M97935_5_at | STAT1 | signal transducer and activator of transcription 1, 91kDa | AFFX-HUMISGF3A/M97935_5 | 6.41 |
| 218145_at | TRIB3 | tribbles homolog 3 (Drosophila) | NM_021158 | 6.41 |
| 202270_at | GBP1 | guanylate binding protein 1, interferon-inducible, 67kDa /// guanylate binding protein 1, interferon-inducible, 67kDa | NM_002053 | 6.40 |
| 227034_at | C2orf26 | chromosome 2 open reading frame 26 | BE669553 | 6.36 |
| 201325_s_at | EMP1 | epithelial membrane protein 1 | NM_001423 | 6.31 |
| 209457_at | DUSP5 | dual specificity phosphatase 5 | U16996 | 6.13 |
| 227262_at | HAPLN3 | hyaluronan and proteoglycan link protein 3 | BE348293 | 6.00 |
| 231577_s_at | GBP1 | guanylate binding protein 1, interferon-inducible, 67kDa | AW014593 | 6.00 |
| 219716_at | APOL6 | apolipoprotein L, 6 | NM_030641 | 5.99 |
| 209875_s_at | SPP1 | secreted phosphoprotein 1 (osteopontin, bone sialoprotein I, early T-lymphocyte activation 1) | M83248 | 5.98 |
| 218451_at | CDCP1 | CUB domain containing protein 1 | NM_022842 | 5.95 |
| 222573_s_at | SAV1 | salvador homolog 1 (Drosophila) | AI679398 | 5.82 |
| 234491_s_at | SAV1 | salvador homolog 1 (Drosophila) | AJ292969 | 5.72 |
| 202269_x_at | GBP1 | guanylate binding protein 1, interferon-inducible, 67kDa /// guanylate binding protein 1, interferon-inducible, 67kDa | BC002666 | 5.64 |
| 209544_at | RIPK2 | receptor-interacting serine-threonine kinase 2 | AF027706 | 5.64 |
| 1568592_at | LOC400368 | hypothetical gene supported by BC031266 | BM976092 | 5.60 |
| 227250_at | KREMEN1 | Kringle containing transmembrane protein 1 | BF221745 | 5.59 |
| 220745_at | IL19 | interleukin 19 | NM_013371 | 5.52 |
| 202307_s_at | TAP1 | transporter 1, ATP-binding cassette, sub-family B (MDR/TAP) | NM_000593 | 5.45 |
| 221223_x_at | CISH | cytokine inducible SH2-containing protein | NM_013324 | 5.42 |
| 219622_at | RAB20 | RAB20, member RAS oncogene family | NM_017817 | 5.42 |
| 209210_s_at | PLEKHC1 | pleckstrin homology domain containing, family C (with FERM domain) member 1 | Z24725 | 5.41 |
| 200887_s_at | STAT1 | signal transducer and activator of transcription 1, 91kDa | NM_007315 | 5.34 |
| 240287_at | LOC341720 | similar to immune-responsive gene 1 | BG236136 | 5.29 |
| AFFX-HUMRGE/M10098_3_at | SRP68 | signal recognition particle 68kDa | AFFX-HUMRGE/M10098_3 | 5.22 |
| 237718_at | EIF4E | eukaryotic translation initiation factor 4E | AA913840 | 5.22 |
| 201324_at | EMP1 | epithelial membrane protein 1 | NM_001423 | 5.17 |
| AFFX-HUMISGF3A/M97935_MB_at | STAT1 | signal transducer and activator of transcription 1, 91kDa | AFFX-HUMISGF3A/M97935_MB | 5.16 |
| 223939_at | SUCNR1 | succinate receptor 1 | AF348078 | 5.15 |
| 1569095_at | --- | Homo sapiens, clone IMAGE:4133286, mRNA | BC016366 | 5.10 |
| 220865_s_at | TPRT | trans-prenyltransferase | NM_014317 | 5.05 |
| 222802_at | EDN1 | endothelin 1 | J05008 | 5.03 |
| 219496_at | C2orf26 | chromosome 2 open reading frame 26 | NM_023016 | 4.95 |
| 240421_x_at | --- | MRNA; cDNA DKFZp313N2115 (from clone DKFZp313N2115) | AI693524 | 4.95 |
| 223961_s_at | CISH | cytokine inducible SH2-containing protein | D83532 | 4.91 |
| 1554997_a_at | PTGS2 | prostaglandin-endoperoxide synthase 2 (prostaglandin G/H synthase and cyclooxygenase) | AY151286 | 4.88 |
| 238725_at | --- | Transcribed locus, weakly similar to XP_496299.1 PREDICTED: hypothetical protein LOC148206 [Homo sapiens] | AW392551 | 4.87 |
| 204089_x_at | MAP3K4 | mitogen-activated protein kinase kinase kinase 4 | NM_006724 | 4.79 |
| 210139_s_at | PMP22 | peripheral myelin protein 22 | L03203 | 4.78 |
| 203887_s_at | THBD | thrombomodulin | NM_000361 | 4.76 |
| 229437_at | BIC | BIC transcript | BG231961 | 4.75 |
| 218951_s_at | PLCXD1 | phosphatidylinositol-specific phospholipase C, X domain containing 1 | NM_018390 | 4.70 |
| 232375_at | STAT1 | Signal transducer and activator of transcription 1, 91kDa | AI539443 | 4.68 |
| 206569_at | IL24 | interleukin 24 | NM_006850 | 4.64 |
| 219270_at | MGC4504 | hypothetical protein MGC4504 | NM_024111 | 4.63 |
| 210118_s_at | IL1A | interleukin 1, alpha | M15329 | 4.58 |
| 226893_at | ABL2 | V-abl Abelson murine leukemia viral oncogene homolog 2 (arg, Abelson-related gene) | AW173164 | 4.56 |
| 215284_at | SNX9 | Sorting nexin 9 | AF070575 | 4.48 |
| 209765_at | ADAM19 | ADAM metallopeptidase domain 19 (meltrin beta) | Y13786 | 4.46 |
| AFFX-HUMISGF3A/M97935_MA_at | STAT1 | signal transducer and activator of transcription 1, 91kDa | AFFX-HUMISGF3A/M97935_MA | 4.46 |
| 228242_at | --- | Transcribed locus | BF055201 | 4.43 |
| 1570007_at | FAD158 | Leucine rich repeat containing 8 family, member C | BC036122 | 4.42 |
| 203888_at | THBD | thrombomodulin | NM_000361 | 4.39 |
| 219412_at | RAB38 | RAB38, member RAS oncogene family | NM_022337 | 4.37 |
| 237252_at | THBD | thrombomodulin | AW119113 | 4.27 |
| 204803_s_at | RRAD | Ras-related associated with diabetes | NM_004165 | 4.23 |
| 202531_at | IRF1 | interferon regulatory factor 1 | NM_002198 | 4.22 |
| 231996_at | N4BP2 | Nedd4 binding protein 2 | AB037834 | 4.17 |
| 229011_at | EMP1 | Epithelial membrane protein 1 | AA150501 | 4.15 |
| 203108_at | GPRC5A | G protein-coupled receptor, family C, group 5, member A | NM_003979 | 4.14 |
| 235175_at | GBP4 | guanylate binding protein 4 | BG260886 | 4.08 |
| 201328_at | ETS2 | v-ets erythroblastosis virus E26 oncogene homolog 2 (avian) | AL575509 | 4.01 |
| 202760_s_at | AKAP2 /// PALM2-AKAP2 | A kinase (PRKA) anchor protein 2 /// PALM2-AKAP2 protein | NM_007203 | 4.00 |
| 235688_s_at | TRAF4 | TNF receptor-associated factor 4 | AI992283 | 4.00 |
| 204015_s_at | DUSP4 | dual specificity phosphatase 4 | BC002671 | 3.98 |
| 206148_at | IL3RA | interleukin 3 receptor, alpha (low affinity) | NM_002183 | 3.98 |
| 236198_at | --- | Transcribed locus | AW292872 | 3.94 |
| 236439_at | BCL6 | B-cell CLL/lymphoma 6 (zinc finger protein 51) | AI733564 | 3.94 |
| 206995_x_at | SCARF1 | scavenger receptor class F, member 1 | NM_003693 | 3.92 |
| 206115_at | EGR3 | early growth response 3 | NM_004430 | 3.91 |
| 205114_s_at | CCL3 /// CCL3L1 /// CCL3L3 | chemokine (C-C motif) ligand 3 /// chemokine (C-C motif) ligand 3-like 1 /// chemokine (C-C motif) ligand 3-like 3 | NM_002983 | 3.89 |
| 211924_s_at | PLAUR | plasminogen activator, urokinase receptor /// plasminogen activator, urokinase receptor | AY029180 | 3.88 |
| 228176_at | EDG3 | endothelial differentiation, sphingolipid G-protein-coupled receptor, 3 | AA534817 | 3.88 |
| 216236_s_at | SLC2A3 /// SLC2A14 | solute carrier family 2 (facilitated glucose transporter), member 3 /// solute carrier family 2 (facilitated glucose transporter), member 14 | AL110298 | 3.87 |
| 236313_at | CDKN2B | cyclin-dependent kinase inhibitor 2B (p15, inhibits CDK4) | AW444761 | 3.84 |
| 201329_s_at | ETS2 | v-ets erythroblastosis virus E26 oncogene homolog 2 (avian) | NM_005239 | 3.84 |
| 201437_s_at | EIF4E | eukaryotic translation initiation factor 4E | NM_001968 | 3.83 |
| 223434_at | GBP3 | guanylate binding protein 3 | AL136680 | 3.82 |
| 230741_at | --- | CDNA FLJ41454 fis, clone BRSTN2011597 | AI655467 | 3.81 |
| 201431_s_at | DPYSL3 | dihydropyrimidinase-like 3 | NM_001387 | 3.80 |
| 208893_s_at | DUSP6 | dual specificity phosphatase 6 | BC005047 | 3.78 |
| 242633_x_at | ZNF75A | Zinc finger protein 75a | AA829635 | 3.75 |
| 235122_at | HIVEP3 | human immunodeficiency virus type I enhancer binding protein 3 | AI800713 | 3.75 |
| 223068_at | EML4 | echinoderm microtubule associated protein like 4 | AV707345 | 3.73 |
| 216243_s_at | IL1RN | interleukin 1 receptor antagonist | BE563442 | 3.72 |
| 204748_at | PTGS2 | prostaglandin-endoperoxide synthase 2 (prostaglandin G/H synthase and cyclooxygenase) | NM_000963 | 3.70 |
| 228314_at | --- | CDNA FLJ37485 fis, clone BRAWH2014379 | BE877357 | 3.70 |
| 212659_s_at | IL1RN | interleukin 1 receptor antagonist | AW083357 | 3.69 |
| 206100_at | CPM | carboxypeptidase M | NM_001874 | 3.68 |
| 223533_at | LRRC8C | leucine rich repeat containing 8 family, member C | AL136919 | 3.68 |
| 201242_s_at | ATP1B1 | ATPase, Na+/K+ transporting, beta 1 polypeptide | BC000006 | 3.67 |
| 226390_at | STARD4 | START domain containing 4, sterol regulated | AA628398 | 3.66 |
| 242727_at | ARL8 | ADP-ribosylation factor-like 8 | BG032269 | 3.61 |
| 227458_at | PDCD1LG1 | CD274 antigen | AI608902 | 3.61 |
| 206267_s_at | MATK | megakaryocyte-associated tyrosine kinase | NM_002378 | 3.60 |
| 227607_at | AMSH-LP | associated molecule with the SH3 domain of STAM (AMSH) like protein | AI638611 | 3.60 |
| 204014_at | DUSP4 | dual specificity phosphatase 4 | NM_001394 | 3.60 |
| 220346_at | MTHFD2L | methylenetetrahydrofolate dehydrogenase (NADP+ dependent) 2-like | NM_025001 | 3.59 |
| 207339_s_at | LTB | lymphotoxin beta (TNF superfamily, member 3) | NM_002341 | 3.58 |
| 206359_at | SOCS3 | suppressor of cytokine signaling 3 | BG035761 | 3.55 |
| 1552711_a_at | FLJ32499 | hypothetical protein FLJ32499 | NM_144607 | 3.55 |
| 201243_s_at | ATP1B1 | ATPase, Na+/K+ transporting, beta 1 polypeptide | NM_001677 | 3.54 |
| 205476_at | CCL20 | chemokine (C-C motif) ligand 20 | NM_004591 | 3.54 |
| 203471_s_at | PLEK | pleckstrin | NM_002664 | 3.53 |
| 207316_at | HAS1 | hyaluronan synthase 1 | NM_001523 | 3.52 |
| 223974_at | MGC11082 | hypothetical protein MGC11082 | BC005130 | 3.51 |
| 202759_s_at | PALM2-AKAP2 | PALM2-AKAP2 protein | BE879367 | 3.50 |
| 1563469_at | ARID5B | AT rich interactive domain 5B (MRF1-like) | AL832681 | 3.49 |
| 202768_at | FOSB | FBJ murine osteosarcoma viral oncogene homolog B | NM_006732 | 3.49 |
| 203725_at | GADD45A | growth arrest and DNA-damage-inducible, alpha | NM_001924 | 3.47 |
| 204279_at | PSMB9 | proteasome (prosome, macropain) subunit, beta type, 9 (large multifunctional peptidase 2) | NM_002800 | 3.46 |
| 1558836_at | --- | MRNA; cDNA DKFZp667A182 (from clone DKFZp667A182) | BQ024490 | 3.45 |
| 201693_s_at | EGR1 | early growth response 1 | AV733950 | 3.42 |
| 202340_x_at | NR4A1 | nuclear receptor subfamily 4, group A, member 1 | NM_002135 | 3.42 |
| 221893_s_at | ADCK2 | aarF domain containing kinase 2 | N32831 | 3.42 |
| 212614_at | ARID5B | AT rich interactive domain 5B (MRF1-like) | BG285011 | 3.42 |
| 226117_at | TIFA | TRAF-interacting protein with a forkhead-associated domain | AA195074 | 3.38 |
| 1558517_s_at | --- | CDNA FLJ37485 fis, clone BRAWH2014379 | CA773938 | 3.38 |
| 212762_s_at | TCF7L2 | transcription factor 7-like 2 (T-cell specific, HMG-box) | AI375916 | 3.37 |
| 231899_at | ZC3H12C | zinc finger CCCH-type containing 12C | AB051513 | 3.37 |
| 205781_at | C16orf7 | chromosome 16 open reading frame 7 | NM_004913 | 3.37 |
| 209795_at | CD69 | CD69 antigen (p60, early T-cell activation antigen) | L07555 | 3.33 |
| 226034_at | --- | Homo sapiens, clone IMAGE:3881549, mRNA | BE222344 | 3.31 |
| 244423_at | C10orf45 | Chromosome 10 open reading frame 45 | R99268 | 3.31 |
| 209545_s_at | RIPK2 | receptor-interacting serine-threonine kinase 2 | AF064824 | 3.31 |
| 203470_s_at | PLEK | pleckstrin | AI433595 | 3.28 |
| 219032_x_at | OPN3 | opsin 3 (encephalopsin, panopsin) | NM_014322 | 3.28 |
| 229390_at | LOC441168 | hypothetical protein LOC441168 | AV734646 | 3.28 |
| 236213_at | NFE2L2 | Nuclear factor (erythroid-derived 2)-like 2 | AI809760 | 3.27 |
| 227404_s_at | EGR1 | Early growth response 1 | AI459194 | 3.27 |
| 203395_s_at | HES1 | hairy and enhancer of split 1, (Drosophila) | NM_005524 | 3.26 |
| 244035_at | BCL2 | B-cell CLL/lymphoma 2 | BF003032 | 3.23 |
| 202988_s_at | RGS1 | regulator of G-protein signalling 1 | NM_002922 | 3.22 |
| 239835_at | KBTBD8 | kelch repeat and BTB (POZ) domain containing 8 | AA669114 | 3.20 |
| 1564274_at | C9orf47 | chromosome 9 open reading frame 47 | AK094842 | 3.20 |
| 206374_at | DUSP8 | dual specificity phosphatase 8 | NM_004420 | 3.20 |
| 226025_at | ANKRD28 | ankyrin repeat domain 28 | AV740426 | 3.20 |
| 226694_at | PALM2-AKAP2 | PALM2-AKAP2 protein | BG540494 | 3.19 |
| 225924_at | FLJ25371 | Hypothetical protein FLJ25371 | AI478634 | 3.18 |
| 205207_at | IL6 | interleukin 6 (interferon, beta 2) | NM_000600 | 3.18 |
| 241869_at | APOL6 | apolipoprotein L, 6 | AW026509 | 3.18 |
| 232829_at | OR52K3P | olfactory receptor, family 52, subfamily K, member 3 pseudogene | AF143328 | 3.17 |
| 242388_x_at | TAGAP | T-cell activation GTPase activating protein | AW576600 | 3.16 |
| 1555962_at | B3GNT7 | UDP-GlcNAc:betaGal beta-1,3-N-acetylglucosaminyltransferase 7 | CA503291 | 3.16 |
| 203140_at | BCL6 | B-cell CLL/lymphoma 6 (zinc finger protein 51) /// B-cell CLL/lymphoma 6 (zinc finger protein 51) | NM_001706 | 3.15 |
| 231313_at | FLJ27354 | hypothetical gene supported by AK130864 | AW134984 | 3.14 |
| 226099_at | ELL2 | elongation factor, RNA polymerase II, 2 | AI924426 | 3.14 |
| 229307_at | ANKRD28 | ankyrin repeat domain 28 | N32051 | 3.14 |
| 214867_at | KIAA0913 | KIAA0913 | AL080182 | 3.13 |
| 204200_s_at | PDGFB | platelet-derived growth factor beta polypeptide (simian sarcoma viral (v-sis) oncogene homolog) | NM_002608 | 3.13 |
| 204802_at | RRAD | Ras-related associated with diabetes | NM_004165 | 3.12 |
| 1557116_at | --- | --- | BM980001 | 3.12 |
| 242907_at | GBP2 | guanylate binding protein 2, interferon-inducible | BF509371 | 3.11 |
| 1563445_x_at | CTSLL3 | cathepsin L-like 3 | L25629 | 3.11 |
| 222870_s_at | B3GNT1 | UDP-GlcNAc:betaGal beta-1,3-N-acetylglucosaminyltransferase 1 | AF288208 | 3.10 |
| 209099_x_at | JAG1 | jagged 1 (Alagille syndrome) | U73936 | 3.08 |
| 235042_at | CMYA1 | cardiomyopathy associated 1 | AW755250 | 3.08 |
| 223027_at | SNX9 | sorting nexin 9 | BF972871 | 3.07 |
| 201866_s_at | NR3C1 | nuclear receptor subfamily 3, group C, member 1 (glucocorticoid receptor) | NM_000176 | 3.07 |
| 203234_at | UPP1 | uridine phosphorylase 1 | NM_003364 | 3.06 |
| 228674_s_at | EML4 | echinoderm microtubule associated protein like 4 | AA524507 | 3.06 |
| 200628_s_at | WARS | tryptophanyl-tRNA synthetase | M61715 | 3.05 |
| 202497_x_at | SLC2A3 | solute carrier family 2 (facilitated glucose transporter), member 3 | AI631159 | 3.05 |
| 230620_at | --- | Transcribed locus | BE550967 | 3.04 |
| 227099_s_at | LOC387763 | hypothetical LOC387763 | AW276078 | 3.03 |
| 214446_at | ELL2 | elongation factor, RNA polymerase II, 2 | NM_012081 | 3.03 |
| 204048_s_at | PHACTR2 | phosphatase and actin regulator 2 | AA551142 | 3.03 |
| 228758_at | --- | Hypothetical LOC389185 | AW264036 | 3.03 |
| 218000_s_at | PHLDA1 | pleckstrin homology-like domain, family A, member 1 | NM_007350 | 3.02 |
| 216015_s_at | CIAS1 | cold autoinflammatory syndrome 1 | AK027194 | 3.01 |
| 221877_at | --- | CDNA FLJ46713 fis, clone TRACH3016885 | BF508835 | 3.00 |
| 230511_at | CREM | cAMP responsive element modulator | AI800640 | 3.00 |
| 201464_x_at | JUN | v-jun sarcoma virus 17 oncogene homolog (avian) | BG491844 | 2.98 |
| 230127_at | --- | Transcribed locus | AW044663 | 2.97 |
| 35666_at | SEMA3F | sema domain, immunoglobulin domain (Ig), short basic domain, secreted, (semaphorin) 3F | U38276 | 2.96 |
| 222303_at | --- | --- | AV700891 | 2.96 |
| 207850_at | CXCL3 | chemokine (C-X-C motif) ligand 3 | NM_002090 | 2.95 |
| 231312_at | --- | Transcribed locus | W87470 | 2.94 |
| 225922_at | FLJ25371 | Hypothetical protein FLJ25371 | BE501838 | 2.93 |
| 44120_at | ADCK2 | aarF domain containing kinase 2 | AI879381 | 2.92 |
| 205101_at | CIITA | class II, major histocompatibility complex, transactivator | NM_000246 | 2.92 |
| 204286_s_at | PMAIP1 | phorbol-12-myristate-13-acetate-induced protein 1 | NM_021127 | 2.91 |
| 1552613_s_at | CDC42SE2 | CDC42 small effector 2 | NM_020240 | 2.91 |
| 217933_s_at | LAP3 | leucine aminopeptidase 3 | NM_015907 | 2.90 |
| 200629_at | WARS | tryptophanyl-tRNA synthetase | NM_004184 | 2.90 |
| 205016_at | TGFA | transforming growth factor, alpha | NM_003236 | 2.90 |
| 204804_at | TRIM21 | tripartite motif-containing 21 | NM_003141 | 2.88 |
| 206513_at | AIM2 | absent in melanoma 2 | NM_004833 | 2.88 |
| 214349_at | --- | Hypothetical LOC388388 | AV764378 | 2.87 |
| 216950_s_at | FCGR1A | Fc fragment of IgG, high affinity Ia, receptor (CD64) | X14355 | 2.87 |
| 212518_at | PIP5K1C | phosphatidylinositol-4-phosphate 5-kinase, type I, gamma | AB011161 | 2.86 |
| 205269_at | LCP2 | lymphocyte cytosolic protein 2 (SH2 domain containing leukocyte protein of 76kDa) | AI123251 | 2.85 |
| 201661_s_at | ACSL3 | acyl-CoA synthetase long-chain family member 3 | NM_004457 | 2.85 |
| 202193_at | LIMK2 | LIM domain kinase 2 | NM_005569 | 2.85 |
| 222088_s_at | SLC2A3 /// SLC2A14 | solute carrier family 2 (facilitated glucose transporter), member 3 /// solute carrier family 2 (facilitated glucose transporter), member 14 | AA778684 | 2.85 |
| 209803_s_at | PHLDA2 | pleckstrin homology-like domain, family A, member 2 | AF001294 | 2.85 |
| 1552612_at | CDC42SE2 | CDC42 small effector 2 | NM_020240 | 2.85 |
| 202907_s_at | NBN | nibrin | NM_002485 | 2.84 |
| 226345_at | ARL8 | ADP-ribosylation factor-like 8 | AW270158 | 2.84 |
| 217997_at | PHLDA1 | pleckstrin homology-like domain, family A, member 1 | AI795908 | 2.84 |
| 207907_at | TNFSF14 | tumor necrosis factor (ligand) superfamily, member 14 | NM_003807 | 2.83 |
| 212298_at | NRP1 | neuropilin 1 | BE620457 | 2.82 |
| 214511_x_at | FCGR1A /// LOC440607 | Fc fragment of IgG, high affinity Ia, receptor (CD64) /// Fc-gamma receptor I B2 | L03419 | 2.81 |
| 1555833_a_at | --- | CDNA FLJ46713 fis, clone TRACH3016885 | AK096168 | 2.81 |
| 205193_at | MAFF | v-maf musculoaponeurotic fibrosarcoma oncogene homolog F (avian) | NM_012323 | 2.81 |
| 214438_at | HLX1 | H2.0-like homeo box 1 (Drosophila) | M60721 | 2.80 |
| 226460_at | KIAA1450 | KIAA1450 protein | AB040883 | 2.80 |
| 1564796_at | EMP1 | epithelial membrane protein 1 | BC017854 | 2.80 |
| 225987_at | STEAP4 | STEAP family member 4 | AA650281 | 2.80 |
| 224057_s_at | THAP4 | THAP domain containing 4 | BC000767 | 2.80 |
| 201466_s_at | JUN | v-jun sarcoma virus 17 oncogene homolog (avian) | NM_002228 | 2.79 |
| 214228_x_at | TNFRSF4 | tumor necrosis factor receptor superfamily, member 4 | AJ277151 | 2.79 |
| 221211_s_at | C21orf7 | chromosome 21 open reading frame 7 | NM_020152 | 2.78 |
| 224392_s_at | OPN3 | opsin 3 (encephalopsin, panopsin) /// opsin 3 (encephalopsin, panopsin) | AF303588 | 2.77 |
| 206239_s_at | SPINK1 | serine peptidase inhibitor, Kazal type 1 | NM_003122 | 2.77 |
| 207091_at | P2RX7 | purinergic receptor P2X, ligand-gated ion channel, 7 | NM_002562 | 2.77 |
| 206522_at | MGAM | maltase-glucoamylase (alpha-glucosidase) | NM_004668 | 2.77 |
| 1559585_at | --- | --- | AK096369 | 2.77 |
| 222402_at | C13orf12 | chromosome 13 open reading frame 12 | BC003390 | 2.76 |
| 213355_at | ST3GAL6 | ST3 beta-galactoside alpha-2,3-sialyltransferase 6 | AI989567 | 2.76 |
| 203373_at | SOCS2 | suppressor of cytokine signaling 2 | NM_003877 | 2.75 |
| 207630_s_at | CREM | cAMP responsive element modulator | NM_001881 | 2.74 |
| 1553785_at | RASGEF1B | RasGEF domain family, member 1B | NM_152545 | 2.74 |
| 202498_s_at | SLC2A3 | solute carrier family 2 (facilitated glucose transporter), member 3 | BE550486 | 2.74 |
| 227807_at | PARP9 | poly (ADP-ribose) polymerase family, member 9 | AI738416 | 2.73 |
| 223028_s_at | SNX9 | sorting nexin 9 | BC005022 | 2.73 |
| 38158_at | ESPL1 | extra spindle poles like 1 (S. cerevisiae) | D79987 | 2.71 |
| 208891_at | DUSP6 | dual specificity phosphatase 6 | BC003143 | 2.71 |
| 214146_s_at | PPBP | pro-platelet basic protein (chemokine (C-X-C motif) ligand 7) | R64130 | 2.71 |
| 218647_s_at | YRDC | yrdC domain containing (E.coli) | NM_024640 | 2.70 |
| 225646_at | CTSC | cathepsin C | AI246687 | 2.70 |
| 201435_s_at | EIF4E | eukaryotic translation initiation factor 4E | AW268640 | 2.69 |
| 204925_at | CTNS | cystinosis, nephropathic | NM_004937 | 2.68 |
| 202906_s_at | NBN | nibrin | AF049895 | 2.68 |
| 226844_at | MOBKL2B | MOB1, Mps One Binder kinase activator-like 2B (yeast) | AI375115 | 2.67 |
| 203006_at | INPP5A | inositol polyphosphate-5-phosphatase, 40kDa | NM_005539 | 2.67 |
| 205088_at | CXorf6 | chromosome X open reading frame 6 | NM_005491 | 2.67 |
| 201354_s_at | BAZ2A | bromodomain adjacent to zinc finger domain, 2A | AA788652 | 2.66 |
| 219938_s_at | PSTPIP2 | proline-serine-threonine phosphatase interacting protein 2 | NM_024430 | 2.66 |
| 36566_at | CTNS | cystinosis, nephropathic | AJ222967 | 2.66 |
| 202672_s_at | ATF3 | activating transcription factor 3 | NM_001674 | 2.65 |
| 221894_at | ADCK2 | aarF domain containing kinase 2 | N32831 | 2.65 |
| 204103_at | CCL4 | chemokine (C-C motif) ligand 4 | NM_002984 | 2.64 |
| 229391_s_at | LOC441168 | hypothetical protein LOC441168 | AV734646 | 2.64 |
| 235711_at | PURB | purine-rich element binding protein B | AA495775 | 2.63 |
| 201694_s_at | EGR1 | early growth response 1 | NM_001964 | 2.63 |
| 235157_at | PARP14 | Poly (ADP-ribose) polymerase family, member 14 | AW297731 | 2.62 |
| 204472_at | GEM | GTP binding protein overexpressed in skeletal muscle | NM_005261 | 2.62 |
| 228648_at | LRG1 | leucine-rich alpha-2-glycoprotein 1 | AA622495 | 2.62 |
| 216061_x_at | PDGFB | platelet-derived growth factor beta polypeptide (simian sarcoma viral (v-sis) oncogene homolog) | AU150748 | 2.62 |
| 206548_at | FLJ23556 | hypothetical protein FLJ23556 | NM_024880 | 2.61 |
| 201430_s_at | DPYSL3 | dihydropyrimidinase-like 3 | W72516 | 2.58 |
| 213457_at | MFHAS1 | malignant fibrous histiocytoma amplified sequence 1 | BF739959 | 2.58 |
| 210845_s_at | PLAUR | plasminogen activator, urokinase receptor | U08839 | 2.57 |
| 228967_at | SUI1 | Eukaryotic translation initiation factor 1 | BE964053 | 2.57 |
| 202499_s_at | SLC2A3 | solute carrier family 2 (facilitated glucose transporter), member 3 | NM_006931 | 2.57 |
| 201865_x_at | NR3C1 | nuclear receptor subfamily 3, group C, member 1 (glucocorticoid receptor) | AI432196 | 2.56 |
| 244598_at | --- | Full length insert cDNA clone ZD66F04 | W72060 | 2.56 |
| 202087_s_at | CTSL | cathepsin L | NM_001912 | 2.56 |
| 1554237_at | SDCCAG8 | serologically defined colon cancer antigen 8 | BC032454 | 2.56 |
| 211984_at | CALM1 | calmodulin 1 (phosphorylase kinase, delta) | AI653730 | 2.56 |
| 205992_s_at | IL15 | interleukin 15 | NM_000585 | 2.55 |
| 222816_s_at | ZCCHC2 | zinc finger, CCHC domain containing 2 | BE676543 | 2.55 |
| 205745_x_at | ADAM17 | ADAM metallopeptidase domain 17 (tumor necrosis factor, alpha, converting enzyme) | NM_003183 | 2.55 |
| 231078_at | --- | --- | H69701 | 2.55 |
| 231412_at | DKFZp686L14188 | hypothetical gene supported by BX538329 | H04388 | 2.54 |
| 232331_at | PPARG | Peroxisome proliferative activated receptor, gamma | AK027107 | 2.54 |
| 1560396_at | KLHL6 | Kelch-like 6 (Drosophila) | AK097976 | 2.54 |
| 231907_at | ABL2 | V-abl Abelson murine leukemia viral oncogene homolog 2 (arg, Abelson-related gene) | AK025877 | 2.53 |
| 231769_at | FBXO6 | F-box protein 6 | AF129536 | 2.53 |
| 239105_at | --- | Transcribed locus | AI681581 | 2.53 |
| 217979_at | TM4SF13 | Tetraspanin 13 | NM_014399 | 2.53 |
| 210074_at | CTSL2 | cathepsin L2 | AF070448 | 2.53 |
| 226914_at | ARPC5L | actin related protein 2/3 complex, subunit 5-like | AU158936 | 2.53 |
| 212312_at | BCL2L1 | BCL2-like 1 /// BCL2-like 1 | AL117381 | 2.52 |
| 233824_at | --- | CDNA: FLJ21428 fis, clone COL04203 | AK025081 | 2.52 |
| 201649_at | UBE2L6 | ubiquitin-conjugating enzyme E2L 6 | NM_004223 | 2.52 |
| 1559203_s_at | KRAS | v-Ki-ras2 Kirsten rat sarcoma viral oncogene homolog | BC029545 | 2.52 |
| 209967_s_at | CREM | cAMP responsive element modulator | D14826 | 2.51 |
| 244322_at | --- | CDNA FLJ38230 fis, clone FCBBF2004448 | AW362008 | 2.50 |
| 208510_s_at | PPARG | peroxisome proliferative activated receptor, gamma | NM_015869 | 2.50 |
| 217752_s_at | CNDP2 | CNDP dipeptidase 2 (metallopeptidase M20 family) | NM_018235 | 2.50 |
| 225557_at | AXUD1 | AXIN1 up-regulated 1 | AI091372 | 2.49 |
| 218723_s_at | RGC32 | response gene to complement 32 | NM_014059 | 2.49 |
| 205220_at | GPR109B | G protein-coupled receptor 109B /// G protein-coupled receptor 109B | NM_006018 | 2.49 |
| 202241_at | TRIB1 | tribbles homolog 1 (Drosophila) | NM_025195 | 2.49 |
| 200632_s_at | NDRG1 | N-myc downstream regulated gene 1 | NM_006096 | 2.48 |
| 1554999_at | RASGEF1B | RasGEF domain family, member 1B | BC036784 | 2.48 |
| 208864_s_at | TXN | thioredoxin | AF313911 | 2.48 |
| 241342_at | TMEM65 | transmembrane protein 65 | BG288115 | 2.47 |
| 205819_at | MARCO | macrophage receptor with collagenous structure /// macrophage receptor with collagenous structure | NM_006770 | 2.47 |
| 90265_at | CENTA1 | centaurin, alpha 1 | AW050627 | 2.47 |
| 202679_at | NPC1 | Niemann-Pick disease, type C1 | NM_000271 | 2.46 |
| 202905_x_at | NBN | nibrin | AI796269 | 2.45 |
| 212500_at | C10orf22 | chromosome 10 open reading frame 22 | AL049319 | 2.45 |
| 230233_at | RASGEF1B | RasGEF domain family, member 1B | BF110534 | 2.44 |
| 228738_at | MGC25181 | hypothetical protein MGC25181 | AI927858 | 2.44 |
| 210582_s_at | LIMK2 | LIM domain kinase 2 | AL117466 | 2.44 |
| 204852_s_at | PTPN7 | protein tyrosine phosphatase, non-receptor type 7 | NM_002832 | 2.44 |
| 218266_s_at | FREQ | frequenin homolog (Drosophila) | NM_014286 | 2.44 |
| 227638_at | KIAA1632 | KIAA1632 | AI393091 | 2.44 |
| 201170_s_at | BHLHB2 | basic helix-loop-helix domain containing, class B, 2 | NM_003670 | 2.43 |
| 228303_at | GALNT6 | UDP-N-acetyl-alpha-D-galactosamine:polypeptide N-acetylgalactosaminyltransferase 6 (GalNAc-T6) | AW014155 | 2.43 |
| 226982_at | ELL2 | elongation factor, RNA polymerase II, 2 | AI745624 | 2.43 |
| 213475_s_at | ITGAL | integrin, alpha L (antigen CD11A (p180), lymphocyte function-associated antigen 1; alpha polypeptide) | AC002310 | 2.41 |
| 210233_at | IL1RAP | interleukin 1 receptor accessory protein | AF167343 | 2.41 |
| 41386_i_at | JMJD3 | jumonji domain containing 3 | AB002344 | 2.41 |
| 223069_s_at | EML4 | echinoderm microtubule associated protein like 4 | AF177377 | 2.40 |
| 216321_s_at | NR3C1 | nuclear receptor subfamily 3, group C, member 1 (glucocorticoid receptor) | X03348 | 2.40 |
| 219631_at | LRP12 | low density lipoprotein-related protein 12 | NM_024937 | 2.40 |
| 211653_x_at | AKR1C2 | aldo-keto reductase family 1, member C2 (dihydrodiol dehydrogenase 2; bile acid binding protein; 3-alpha hydroxysteroid dehydrogenase, type III) /// aldo-keto reductase family 1, member C2 (dihydrodiol dehydrogenase 2; bile acid binding protein; 3-alpha hydroxysteroid dehydrogenase, type III) | M33376 | 2.40 |
| 211671_s_at | NR3C1 | nuclear receptor subfamily 3, group C, member 1 (glucocorticoid receptor) /// nuclear receptor subfamily 3, group C, member 1 (glucocorticoid receptor) | U01351 | 2.40 |
| 225612_s_at | B3GNT5 | UDP-GlcNAc:betaGal beta-1,3-N-acetylglucosaminyltransferase 5 | BE672260 | 2.40 |
| 220386_s_at | EML4 | echinoderm microtubule associated protein like 4 | NM_019063 | 2.39 |
| 1559204_x_at | KRAS | v-Ki-ras2 Kirsten rat sarcoma viral oncogene homolog | BC029545 | 2.38 |
| 205239_at | AREG | amphiregulin (schwannoma-derived growth factor) | NM_001657 | 2.38 |
| 226038_at | LONRF1 | LON peptidase N-terminal domain and ring finger 1 | BF680438 | 2.35 |
| 209039_x_at | EHD1 | EH-domain containing 1 | AF001434 | 2.35 |
| 211985_s_at | CALM1 | calmodulin 1 (phosphorylase kinase, delta) | AI653730 | 2.35 |
| 205681_at | BCL2A1 | BCL2-related protein A1 | NM_004049 | 2.35 |
| 228899_at | LOC441297 | LOC441297 | AI870903 | 2.35 |
| 235592_at | ELL2 | Elongation factor, RNA polymerase II, 2 | AW960145 | 2.34 |
| 200808_s_at | ZYX | zyxin | NM_003461 | 2.34 |
| 202748_at | GBP2 | guanylate binding protein 2, interferon-inducible /// guanylate binding protein 2, interferon-inducible | NM_004120 | 2.34 |
| 223196_s_at | SESN2 | sestrin 2 | AL136551 | 2.34 |
| 240674_at | JARID2 | Jumonji, AT rich interactive domain 2 | AW445218 | 2.34 |
| 222221_x_at | EHD1 | EH-domain containing 1 | AY007161 | 2.33 |
| 203276_at | LMNB1 | lamin B1 | NM_005573 | 2.33 |
| 201662_s_at | ACSL3 | acyl-CoA synthetase long-chain family member 3 | D89053 | 2.33 |
| 201280_s_at | DAB2 | disabled homolog 2, mitogen-responsive phosphoprotein (Drosophila) | NM_001343 | 2.33 |
| 224298_s_at | PHGDHL1 | phosphoglycerate dehydrogenase like 1 | BC004528 | 2.31 |
| 225955_at | METRNL | meteorin, glial cell differentiation regulator-like | BG231494 | 2.31 |
| 1560397_s_at | KLHL6 | kelch-like 6 (Drosophila) | AK097976 | 2.31 |
| 206420_at | IGSF6 | immunoglobulin superfamily, member 6 | NM_005849 | 2.31 |
| 227762_at | --- | Transcribed locus | AW244016 | 2.31 |
| 207375_s_at | IL15RA | interleukin 15 receptor, alpha | NM_002189 | 2.31 |
| 213618_at | CENTD1 | centaurin, delta 1 | AB011152 | 2.30 |
| 219150_s_at | CENTA1 | centaurin, alpha 1 | NM_006869 | 2.30 |
| 229715_at | --- | CDNA FLJ41663 fis, clone FEBRA2027297 | AW006182 | 2.30 |
| 214508_x_at | CREM | cAMP responsive element modulator | U44836 | 2.29 |
| 206868_at | STARD8 | START domain containing 8 | NM_014725 | 2.28 |
| 212803_at | NAB2 | NGFI-A binding protein 2 (EGR1 binding protein 2) | BF337329 | 2.28 |
| 202284_s_at | CDKN1A | cyclin-dependent kinase inhibitor 1A (p21, Cip1) | NM_000389 | 2.28 |
| 202181_at | KIAA0247 | KIAA0247 | NM_014734 | 2.28 |
| 225604_s_at | C9orf19 | chromosome 9 open reading frame 19 | AA284532 | 2.26 |
| 231956_at | KIAA1618 | KIAA1618 | AA976354 | 2.26 |
| 225478_at | MFHAS1 | Malignant fibrous histiocytoma amplified sequence 1 | BE783723 | 2.25 |
| 209301_at | CA2 | carbonic anhydrase II | M36532 | 2.24 |
| 231297_at | DOT1L | DOT1-like, histone H3 methyltransferase (S. cerevisiae) | AI479899 | 2.24 |
| 229723_at | TAGAP | T-cell activation GTPase activating protein | BF591040 | 2.24 |
| 239648_at | DCUN1D3 | DCN1, defective in cullin neddylation 1, domain containing 3 (S. cerevisiae) | AI765327 | 2.22 |
| 202638_s_at | ICAM1 | intercellular adhesion molecule 1 (CD54), human rhinovirus receptor | NM_000201 | 2.22 |
| 210357_s_at | SMOX | spermine oxidase | BC000669 | 2.22 |
| 232431_at | NR3C1 | Nuclear receptor subfamily 3, group C, member 1 (glucocorticoid receptor) | AI934556 | 2.22 |
| 244026_at | ELL2 | Elongation factor, RNA polymerase II, 2 | BF063657 | 2.22 |
| 231255_at | --- | --- | AI970090 | 2.22 |
| 202637_s_at | ICAM1 | intercellular adhesion molecule 1 (CD54), human rhinovirus receptor | AI608725 | 2.21 |
| 204285_s_at | PMAIP1 | phorbol-12-myristate-13-acetate-induced protein 1 | AI857639 | 2.21 |
| 219492_at | CHIC2 | cysteine-rich hydrophobic domain 2 | NM_012110 | 2.20 |
| 217769_s_at | C13orf12 | chromosome 13 open reading frame 12 | NM_015932 | 2.20 |
| 208991_at | STAT3 | signal transducer and activator of transcription 3 (acute-phase response factor) | AA634272 | 2.20 |
| 204769_s_at | TAP2 | transporter 2, ATP-binding cassette, sub-family B (MDR/TAP) | M74447 | 2.19 |
| 1558837_a_at | --- | MRNA; cDNA DKFZp667A182 (from clone DKFZp667A182) | BQ024490 | 2.18 |
| 241837_at | ARID5B | AT rich interactive domain 5B (MRF1-like) | AI289774 | 2.18 |
| 204401_at | KCNN4 | potassium intermediate/small conductance calcium-activated channel, subfamily N, member 4 | NM_002250 | 2.18 |
| 226201_at | DOT1L | DOT1-like, histone H3 methyltransferase (S. cerevisiae) | AI224128 | 2.17 |
| 216563_at | ANKRD12 | Ankyrin repeat domain 12 | X80821 | 2.16 |
| 207376_at | VENTX | VENT homeobox homolog (Xenopus laevis) | NM_014468 | 2.16 |
| 217823_s_at | UBE2J1 | ubiquitin-conjugating enzyme E2, J1 (UBC6 homolog, yeast) | AL562528 | 2.16 |
| 1555976_s_at | --- | LOC440476 | BU676221 | 2.14 |
| 224920_x_at | MYADM | myeloid-associated differentiation marker | AA909044 | 2.14 |
| 218695_at | EXOSC4 | exosome component 4 | NM_019037 | 2.13 |
| 239331_at | --- | Transcribed locus | AW954199 | 2.12 |
| 240655_at | ALCAM | Activated leukocyte cell adhesion molecule | BE502785 | 2.12 |
| 235221_at | CBLN3 | cerebellin 3 precursor | R52665 | 2.12 |
| 211702_s_at | USP32 | ubiquitin specific peptidase 32 /// ubiquitin specific peptidase 32 | AF350251 | 2.11 |
| 203433_at | MTHFS | 5,10-methenyltetrahydrofolate synthetase (5-formyltetrahydrofolate cyclo-ligase) | NM_006441 | 2.11 |
| 200907_s_at | KIAA0992 | palladin | AU157932 | 2.09 |
| 201465_s_at | JUN | v-jun sarcoma virus 17 oncogene homolog (avian) | BC002646 | 2.09 |
| 200983_x_at | CD59 | CD59 antigen p18-20 (antigen identified by monoclonal antibodies 16.3A5, EJ16, EJ30, EL32 and G344) | BF983379 | 2.06 |
| 218599_at | REC8L1 | REC8-like 1 (yeast) | NM_005132 | 2.04 |
| 225767_at | LOC284801 | hypothetical protein LOC284801 | AI825833 | 2.02 |
| 210621_s_at | RASA1 | RAS p21 protein activator (GTPase activating protein) 1 | M23612 | -2.02 |
| 201939_at | PLK2 | polo-like kinase 2 (Drosophila) | NM_006622 | -2.03 |
| 229394_s_at | GRLF1 | Glucocorticoid receptor DNA binding factor 1 | AI275597 | -2.03 |
| 209201_x_at | CXCR4 | chemokine (C-X-C motif) receptor 4 | L01639 | -2.04 |
| 201301_s_at | ANXA4 | annexin A4 | BC000182 | -2.04 |
| 200896_x_at | HDGF | hepatoma-derived growth factor (high-mobility group protein 1-like) | NM_004494 | -2.04 |
| 229510_at | NYD-SP21 | testes development-related NYD-SP21 | AL044520 | -2.08 |
| 220926_s_at | C1orf22 | chromosome 1 open reading frame 22 | NM_025191 | -2.08 |
| 1557170_at | NEK8 | NIMA (never in mitosis gene a)- related kinase 8 | AI073943 | -2.08 |
| 206688_s_at | CPSF4 | cleavage and polyadenylation specific factor 4, 30kDa | NM_006693 | -2.08 |
| 1557797_a_at | ZFHX1B | Zinc finger homeobox 1b | AW611486 | -2.08 |
| 213649_at | SFRS7 | splicing factor, arginine/serine-rich 7, 35kDa | AA524053 | -2.08 |
| 224952_at | DKFZP564D166 | putative ankyrin-repeat containing protein | BF115054 | -2.08 |
| 202459_s_at | LPIN2 | lipin 2 | U55968 | -2.09 |
| 225160_x_at | MGC5370 | hypothetical protein MGC5370 | AI952357 | -2.09 |
| 228145_s_at | ZNF398 | zinc finger protein 398 | AI950078 | -2.09 |
| 203789_s_at | SEMA3C | sema domain, immunoglobulin domain (Ig), short basic domain, secreted, (semaphorin) 3C | NM_006379 | -2.10 |
| 229560_at | TLR8 | toll-like receptor 8 | AW872374 | -2.11 |
| 203509_at | SORL1 | sortilin-related receptor, L(DLR class) A repeats-containing | NM_003105 | -2.11 |
| 225327_at | FLJ10980 | hypothetical protein FLJ10980 | AB037791 | -2.11 |
| 230206_at | DOCK5 | Dedicator of cytokinesis 5 | AI692645 | -2.12 |
| 233013_x_at | RPL7L1 /// LOC401197 | ribosomal protein L7-like 1 /// similar to RPL7L1 protein | AU146717 | -2.13 |
| 222981_s_at | RAB10 | RAB10, member RAS oncogene family | BC000896 | -2.14 |
| 200708_at | GOT2 | glutamic-oxaloacetic transaminase 2, mitochondrial (aspartate aminotransferase 2) | NM_002080 | -2.15 |
| 222113_s_at | EPS15L1 | epidermal growth factor receptor pathway substrate 15-like 1 | AV710549 | -2.15 |
| 222806_s_at | C11orf30 | chromosome 11 open reading frame 30 | AK023651 | -2.16 |
| 222642_s_at | TMEM33 | transmembrane protein 33 | BC000948 | -2.16 |
| 217862_at | PIAS1 | protein inhibitor of activated STAT, 1 | N24868 | -2.17 |
| 204639_at | ADA | adenosine deaminase | NM_000022 | -2.17 |
| 242903_at | IFNGR1 | Interferon gamma receptor 1 | AI458949 | -2.18 |
| 210378_s_at | SSNA1 | Sjogren's syndrome nuclear autoantigen 1 | BC004118 | -2.18 |
| 209342_s_at | IKBKB | inhibitor of kappa light polypeptide gene enhancer in B-cells, kinase beta | AF080158 | -2.19 |
| 217954_s_at | PHF3 | PHD finger protein 3 | NM_015153 | -2.19 |
| 224962_at | LOC90120 | hypothetical gene supported by AK023162 | AU153030 | -2.19 |
| 202051_s_at | ZNF262 | zinc finger protein 262 | NM_005095 | -2.19 |
| 203604_at | ZNF516 | zinc finger protein 516 | N38750 | -2.19 |
| 201209_at | HDAC1 | histone deacetylase 1 | NM_004964 | -2.20 |
| 225114_at | AGPS | Alkylglycerone phosphate synthase | AA127674 | -2.20 |
| 212381_at | USP24 | ubiquitin specific peptidase 24 | BF444943 | -2.20 |
| 218366_x_at | FLJ20859 | FLJ20859 gene | NM_022734 | -2.22 |
| 209141_at | UBE2G1 | ubiquitin-conjugating enzyme E2G 1 (UBC7 homolog, yeast) | AW299555 | -2.22 |
| 230836_at | ST8SIA4 | ST8 alpha-N-acetyl-neuraminide alpha-2,8-sialyltransferase 4 | AI422986 | -2.23 |
| 203132_at | RB1 | retinoblastoma 1 (including osteosarcoma) | NM_000321 | -2.23 |
| 218486_at | KLF11 | Kruppel-like factor 11 | AA149594 | -2.24 |
| 227087_at | INPP4A | Inositol polyphosphate-4-phosphatase, type I, 107kDa | AA126419 | -2.24 |
| 201107_s_at | THBS1 | thrombospondin 1 | AI812030 | -2.25 |
| 224904_at | PDPR | pyruvate dehydrogenase phosphatase regulatory subunit | AV724415 | -2.25 |
| 214431_at | GMPS | guanine monphosphate synthetase | NM_003875 | -2.25 |
| 221766_s_at | FAM46A | family with sequence similarity 46, member A | AW246673 | -2.26 |
| 201717_at | MRPL49 | mitochondrial ribosomal protein L49 | NM_004927 | -2.26 |
| 218224_at | PNMA1 | paraneoplastic antigen MA1 | NM_006029 | -2.26 |
| 212437_at | CENPB | centromere protein B, 80kDa | AL109804 | -2.26 |
| 203940_s_at | VASH1 | vasohibin 1 | NM_014909 | -2.26 |
| 204725_s_at | NCK1 | NCK adaptor protein 1 | NM_006153 | -2.27 |
| 236125_at | DKFZp586I1420 | Hypothetical protein DKFZp586I1420 | AA832073 | -2.27 |
| 201591_s_at | NISCH | nischarin | NM_007184 | -2.28 |
| 205922_at | VNN2 | vanin 2 /// vanin 2 | NM_004665 | -2.28 |
| 201212_at | LGMN | legumain | D55696 | -2.28 |
| 212606_at | WDFY3 | WD repeat and FYVE domain containing 3 | AL536319 | -2.29 |
| 219041_s_at | REPIN1 | replication initiator 1 | NM_014374 | -2.29 |
| 224445_s_at | ZFYVE21 | zinc finger, FYVE domain containing 21 /// zinc finger, FYVE domain containing 21 | BC005999 | -2.31 |
| 213045_at | MAST3 | microtubule associated serine/threonine kinase 3 | AB011133 | -2.31 |
| 224452_s_at | MGC12966 | hypothetical protein MGC12966 /// hypothetical protein MGC12966 | BC006110 | -2.31 |
| 218555_at | ANAPC2 | anaphase promoting complex subunit 2 | NM_013366 | -2.31 |
| 201743_at | CD14 | CD14 antigen /// CD14 antigen | NM_000591 | -2.32 |
| 1565162_s_at | MGST1 | microsomal glutathione S-transferase 1 | D16947 | -2.32 |
| 218938_at | FBXL15 | F-box and leucine-rich repeat protein 15 | NM_024326 | -2.32 |
| 229980_s_at | SNX5 | sorting nexin 5 | AA131508 | -2.32 |
| 240843_at | PTPN2 | Protein tyrosine phosphatase, non-receptor type 2 | N53696 | -2.32 |
| 226092_at | MPP5 | membrane protein, palmitoylated 5 (MAGUK p55 subfamily member 5) | BF115203 | -2.33 |
| 225904_at | C1orf96 | chromosome 1 open reading frame 96 | N64686 | -2.34 |
| 226853_at | BMP2K | BMP2 inducible kinase | AU145366 | -2.34 |
| 1552553_a_at | CARD12 | caspase recruitment domain family, member 12 | NM_021209 | -2.34 |
| 202169_s_at | AASDHPPT | aminoadipate-semialdehyde dehydrogenase-phosphopantetheinyl transferase | AF302110 | -2.34 |
| 219093_at | FLJ20701 | hypothetical protein FLJ20701 /// hypothetical protein FLJ20701 | NM_017933 | -2.34 |
| 205865_at | ARID3A | AT rich interactive domain 3A (BRIGHT- like) | NM_005224 | -2.35 |
| 222605_at | RCOR3 | REST corepressor 3 | AI807073 | -2.37 |
| 202050_s_at | ZNF262 | zinc finger protein 262 | AI650586 | -2.37 |
| 229354_at | PDCD6 /// AHRR | programmed cell death 6 /// aryl-hydrocarbon receptor repressor | AB033060 | -2.38 |
| 224918_x_at | MGST1 | microsomal glutathione S-transferase 1 | AI220117 | -2.38 |
| 200060_s_at | RNPS1 | RNA binding protein S1, serine-rich domain /// RNA binding protein S1, serine-rich domain | BC001659 | -2.38 |
| 203232_s_at | ATXN1 | ataxin 1 | NM_000332 | -2.38 |
| 228771_at | ADRBK2 | adrenergic, beta, receptor kinase 2 | AI651212 | -2.39 |
| 221986_s_at | KLHL24 | kelch-like 24 (Drosophila) | AW006750 | -2.39 |
| 212499_s_at | C14orf111 /// C14orf32 | chromosome 14 open reading frame 111 /// chromosome 14 open reading frame 32 | AK025580 | -2.40 |
| 212910_at | THAP11 | THAP domain containing 11 | W19873 | -2.40 |
| 205844_at | VNN1 | vanin 1 /// vanin 1 | NM_004666 | -2.40 |
| 216361_s_at | MYST3 | MYST histone acetyltransferase (monocytic leukemia) 3 | AJ251844 | -2.40 |
| 226640_at | LOC221955 | KCCR13L | AA015606 | -2.41 |
| 230426_at | DLD | Dihydrolipoamide dehydrogenase (E3 component of pyruvate dehydrogenase complex, 2-oxo-glutarate complex, branched chain keto acid dehydrogenase complex) | BF212846 | -2.41 |
| 215780_s_at | SET /// LOC389168 | SET translocation (myeloid leukemia-associated) /// similar to SET protein (Phosphatase 2A inhibitor I2PP2A) (I-2PP2A) (Template activating factor I) (TAF-I) (HLA-DR associated protein II) (PHAPII) (Inhibitor of granzyme A-activated DNase) (IGAAD) | Z95126 | -2.41 |
| 225225_at | OIP5 | CDNA clone IMAGE:5302006 /// Opa interacting protein 5 | BF791544 | -2.42 |
| 201185_at | HTRA1 | HtrA serine peptidase 1 | NM_002775 | -2.42 |
| 227698_s_at | RAB40C | RAB40C, member RAS oncogene family | AW007215 | -2.45 |
| 223423_at | GPR160 | G protein-coupled receptor 160 | BC000181 | -2.45 |
| 222623_s_at | ZNF639 | zinc finger protein 639 | BF001614 | -2.45 |
| 1552386_at | FLJ33641 | hypothetical protein FLJ33641 | NM_152687 | -2.46 |
| 230292_at | CHC1L | Regulator of chromosome condensation (RCC1) and BTB (POZ) domain containing protein 2 | AA868809 | -2.46 |
| 227897_at | RAP2B | RAP2B, member of RAS oncogene family | N20927 | -2.46 |
| 212560_at | C11orf32 | chromosome 11 open reading frame 32 | AV728268 | -2.48 |
| 204135_at | DOC1 | downregulated in ovarian cancer 1 | NM_014890 | -2.48 |
| 59644_at | BMP2K | BMP2 inducible kinase | AI735391 | -2.49 |
| 221664_s_at | F11R | F11 receptor | AF154005 | -2.49 |
| 204206_at | MNT | MAX binding protein | NM_020310 | -2.49 |
| 224492_s_at | ZNF627 | zinc finger protein 627 /// zinc finger protein 627 | BC006279 | -2.49 |
| 208613_s_at | FLNB | filamin B, beta (actin binding protein 278) | AV712733 | -2.49 |
| 227227_at | --- | CDNA FLJ32605 fis, clone STOMA1000175 | AI344332 | -2.50 |
| 223179_at | YPEL3 | yippee-like 3 (Drosophila) | BC005009 | -2.50 |
| 204860_s_at | BIRC1 | baculoviral IAP repeat-containing 1 | AI817801 | -2.51 |
| 1556744_a_at | LOC440971 | similar to Zinc finger protein Rlf (Rearranged L-myc fusion gene protein) (Zn-15 related protein) | AI732587 | -2.51 |
| 204759_at | RCBTB2 | regulator of chromosome condensation (RCC1) and BTB (POZ) domain containing protein 2 | NM_001268 | -2.51 |
| 209721_s_at | HOM-TES-103 | HOM-TES-103 tumor antigen-like | BC002857 | -2.51 |
| 229958_at | C8orf61 | chromosome 8 open reading frame 61 | W93695 | -2.51 |
| 223113_at | HSPC196 | hypothetical protein HSPC196 | AF151030 | -2.52 |
| 219603_s_at | ZNF226 | zinc finger protein 226 | NM_015919 | -2.52 |
| 31874_at | GAS2L1 | growth arrest-specific 2 like 1 | Y07846 | -2.53 |
| 212760_at | UBR2 | ubiquitin protein ligase E3 component n-recognin 2 | AB002347 | -2.54 |
| 239001_at | MGST1 | Microsomal glutathione S-transferase 1 | AV705233 | -2.54 |
| 202365_at | MGC5139 | hypothetical protein MGC5139 | BC004815 | -2.54 |
| 202630_at | APPBP2 | amyloid beta precursor protein (cytoplasmic tail) binding protein 2 | AA046411 | -2.54 |
| 221808_at | RAB9A | RAB9A, member RAS oncogene family | NM_004251 | -2.54 |
| 204972_at | OAS2 | 2'-5'-oligoadenylate synthetase 2, 69/71kDa | NM_016817 | -2.54 |
| 228499_at | PFKFB4 | 6-phosphofructo-2-kinase/fructose-2,6-biphosphatase 4 | AL038787 | -2.55 |
| 240440_at | NPL | N-acetylneuraminate pyruvate lyase (dihydrodipicolinate synthase) | AI368358 | -2.56 |
| 221731_x_at | CSPG2 | chondroitin sulfate proteoglycan 2 (versican) | BF218922 | -2.56 |
| 208981_at | PECAM1 | platelet/endothelial cell adhesion molecule (CD31 antigen) | AA702701 | -2.58 |
| 227972_at | TOR2A | torsin family 2, member A | AA873275 | -2.59 |
| 212934_at | LOC137886 | hypothetical protein LOC137886 | AI245523 | -2.60 |
| 209864_at | FRAT2 | frequently rearranged in advanced T-cell lymphomas 2 | AB045118 | -2.61 |
| 208983_s_at | PECAM1 | platelet/endothelial cell adhesion molecule (CD31 antigen) | M37780 | -2.62 |
| 208070_s_at | REV3L | REV3-like, catalytic subunit of DNA polymerase zeta (yeast) | NM_002912 | -2.62 |
| 218815_s_at | TMEM51 | transmembrane protein 51 | NM_018022 | -2.62 |
| 231736_x_at | MGST1 | microsomal glutathione S-transferase 1 | NM_020300 | -2.63 |
| 202519_at | MONDOA | MondoA | NM_014938 | -2.64 |
| 219340_s_at | CLN8 | ceroid-lipofuscinosis, neuronal 8 (epilepsy, progressive with mental retardation) | AF123759 | -2.64 |
| 238496_at | WHSC1L1 | Wolf-Hirschhorn syndrome candidate 1-like 1 | AA741074 | -2.64 |
| 219944_at | RSNL2 | restin-like 2 | NM_024692 | -2.64 |
| 230141_at | ARID4A | AT rich interactive domain 4A (RBP1-like) | AI640594 | -2.64 |
| 226832_at | --- | Hypothetical LOC389188 | BF978778 | -2.65 |
| 209306_s_at | SWAP70 | SWAP-70 protein | AI139569 | -2.65 |
| 1570420_at | STXBP2 | syntaxin binding protein 2 | AF318317 | -2.65 |
| 204276_at | TK2 | thymidine kinase 2, mitochondrial | BE895437 | -2.66 |
| 228532_at | C1orf162 | chromosome 1 open reading frame 162 | AW662189 | -2.66 |
| 203825_at | BRD3 | bromodomain containing 3 | NM_007371 | -2.67 |
| 202227_s_at | BRD8 | bromodomain containing 8 | NM_006696 | -2.68 |
| 220528_at | VNN3 | vanin 3 | NM_018399 | -2.69 |
| 233955_x_at | CXXC5 | CXXC finger 5 | AK001782 | -2.69 |
| 211962_s_at | ZFP36L1 | zinc finger protein 36, C3H type-like 1 | BG250310 | -2.69 |
| 202005_at | ST14 | suppression of tumorigenicity 14 (colon carcinoma, matriptase, epithin) | NM_021978 | -2.69 |
| 1568678_s_at | FGFR1OP | FGFR1 oncogene partner | BC037785 | -2.69 |
| 212484_at | FAM89B | family with sequence similarity 89, member B | BF974389 | -2.69 |
| 219972_s_at | C14orf135 | chromosome 14 open reading frame 135 | NM_022495 | -2.69 |
| 227493_s_at | KIAA1143 | KIAA1143 | AI863484 | -2.69 |
| 218146_at | GLT8D1 | glycosyltransferase 8 domain containing 1 | NM_018446 | -2.70 |
| 202151_s_at | UBADC1 | ubiquitin associated domain containing 1 | NM_016172 | -2.70 |
| 204620_s_at | CSPG2 | chondroitin sulfate proteoglycan 2 (versican) | NM_004385 | -2.71 |
| 220066_at | CARD15 | caspase recruitment domain family, member 15 | NM_022162 | -2.71 |
| 238142_at | KCTD13 | Potassium channel tetramerisation domain containing 13 | AW029203 | -2.72 |
| 203359_s_at | MYCBP | c-myc binding protein | AL525412 | -2.73 |
| 225022_at | GOPC | golgi associated PDZ and coiled-coil motif containing | AW271409 | -2.73 |
| 215338_s_at | NKTR | natural killer-tumor recognition sequence | AI688640 | -2.74 |
| 224516_s_at | CXXC5 | CXXC finger 5 /// CXXC finger 5 | BC006428 | -2.74 |
| 201996_s_at | SPEN | spen homolog, transcriptional regulator (Drosophila) | AL524033 | -2.75 |
| 216705_s_at | ADA | adenosine deaminase | X02189 | -2.78 |
| 210282_at | ZNF198 | zinc finger protein 198 | AL136621 | -2.78 |
| 222996_s_at | CXXC5 | CXXC finger 5 | BC002490 | -2.79 |
| 238604_at | --- | CDNA FLJ25559 fis, clone JTH02834 | AA768884 | -2.83 |
| 201506_at | TGFBI | transforming growth factor, beta-induced, 68kDa | NM_000358 | -2.84 |
| 212388_at | USP24 | ubiquitin specific peptidase 24 | AB028980 | -2.84 |
| 222000_at | LOC339448 | hypothetical protein LOC339448 | AI915947 | -2.86 |
| 241458_at | HYPB | Huntingtin interacting protein B | AI868267 | -2.88 |
| 209267_s_at | SLC39A8 | solute carrier family 39 (zinc transporter), member 8 | AB040120 | -2.89 |
| 233841_s_at | SDS3 | SDS3 protein | AK026749 | -2.89 |
| 223204_at | DKFZp434L142 | hypothetical protein DKFZp434L142 | AF260333 | -2.91 |
| 204072_s_at | 13CDNA73 | hypothetical protein CG003 | NM_023037 | -2.91 |
| 222587_s_at | GALNT7 | UDP-N-acetyl-alpha-D-galactosamine:polypeptide N-acetylgalactosaminyltransferase 7 (GalNAc-T7) | BF699855 | -2.92 |
| 204059_s_at | ME1 | malic enzyme 1, NADP(+)-dependent, cytosolic | NM_002395 | -2.93 |
| 209307_at | SWAP70 | SWAP-70 protein | AB014540 | -2.93 |
| 238077_at | KCTD6 | potassium channel tetramerisation domain containing 6 | T75480 | -2.95 |
| 202114_at | SNX2 | sorting nexin 2 | NM_003100 | -2.95 |
| 219546_at | BMP2K | BMP2 inducible kinase | NM_017593 | -2.98 |
| 201701_s_at | PGRMC2 | progesterone receptor membrane component 2 | NM_006320 | -2.98 |
| 207966_s_at | GLG1 | golgi apparatus protein 1 | NM_012201 | -3.00 |
| 213425_at | WNT5A | wingless-type MMTV integration site family, member 5A /// wingless-type MMTV integration site family, member 5A | AI968085 | -3.01 |
| 58780_s_at | FLJ10357 | hypothetical protein FLJ10357 | R42449 | -3.02 |
| 210111_s_at | KIAA0265 | KIAA0265 protein | AF277175 | -3.02 |
| 222387_s_at | VPS35 | vacuolar protein sorting 35 (yeast) | BG476669 | -3.04 |
| 215646_s_at | CSPG2 | chondroitin sulfate proteoglycan 2 (versican) /// chondroitin sulfate proteoglycan 2 (versican) | R94644 | -3.05 |
| 202622_s_at | ATXN2 | ataxin 2 | NM_002973 | -3.07 |
| 214560_at | FPRL2 | formyl peptide receptor-like 2 | NM_002030 | -3.09 |
| 221983_at | C2orf17 | chromosome 2 open reading frame 17 | AL040896 | -3.11 |
| 209568_s_at | RGL1 | ral guanine nucleotide dissociation stimulator-like 1 | AF186779 | -3.12 |
| 213916_at | ZNF20 | zinc finger protein 20 (KOX 13) | AU154474 | -3.12 |
| 221565_s_at | FAM26B | family with sequence similarity 26, member B | BC000039 | -3.14 |
| 225318_at | WHSC1L1 | Wolf-Hirschhorn syndrome candidate 1-like 1 | AW292751 | -3.14 |
| 211571_s_at | CSPG2 | chondroitin sulfate proteoglycan 2 (versican) | D32039 | -3.15 |
| 212830_at | EGFL5 | EGF-like-domain, multiple 5 | W68084 | -3.18 |
| 222876_s_at | CENTA2 | centaurin, alpha 2 | AI761520 | -3.29 |
| 226214_at | MIR16 | membrane interacting protein of RGS16 | BE677894 | -3.33 |
| 220326_s_at | FLJ10357 | hypothetical protein FLJ10357 | NM_018071 | -3.34 |
| 210660_at | LILRA1 | leukocyte immunoglobulin-like receptor, subfamily A (with TM domain), member 1 /// leukocyte immunoglobulin-like receptor, subfamily A (with TM domain), member 1 | AF025529 | -3.34 |
| 36030_at | HOM-TES-103 | HOM-TES-103 tumor antigen-like | AL080214 | -3.37 |
| 229937_x_at | LILRB1 | Leukocyte immunoglobulin-like receptor, subfamily B (with TM and ITIM domains), member 3 | AI681260 | -3.40 |
| 202052_s_at | RAI14 | retinoic acid induced 14 | NM_015577 | -3.44 |
| 206978_at | CCR2 | chemokine (C-C motif) receptor 2 /// chemokine (C-C motif) receptor 2 | NM_000647 | -3.44 |
| 225731_at | KIAA1223 | KIAA1223 protein | BF196876 | -3.49 |
| 227020_at | YPEL2 | yippee-like 2 (Drosophila) | BE502982 | -3.51 |
| 204619_s_at | CSPG2 | chondroitin sulfate proteoglycan 2 (versican) | BF590263 | -3.53 |
| 225918_at | LOC146346 | hypothetical protein LOC146346 | AI742940 | -3.54 |
| 207167_at | IGSF2 | immunoglobulin superfamily, member 2 | NM_004258 | -3.56 |
| 205990_s_at | WNT5A | wingless-type MMTV integration site family, member 5A | NM_003392 | -3.67 |
| 225953_at | P15RS | hypothetical protein FLJ10656 | AI684281 | -3.78 |
| 219694_at | FLJ11127 | hypothetical protein FLJ11127 | NM_019018 | -3.94 |
| 230422_at | FPRL2 | formyl peptide receptor-like 2 | AW026543 | -3.95 |
| 219869_s_at | SLC39A8 | solute carrier family 39 (zinc transporter), member 8 | NM_022154 | -3.97 |
| 227195_at | ZNF503 | zinc finger protein 503 | AA603467 | -4.03 |
| 230707_at | SORL1 | sortilin-related receptor, L(DLR class) A repeats-containing | AA290609 | -4.07 |
| 235798_at | --- | Transcribed locus, weakly similar to XP_517655.1 PREDICTED: similar to KIAA0825 protein [Pan troglodytes] | BE671123 | -4.14 |
| 221572_s_at | SLC26A6 | solute carrier family 26, member 6 | AF288410 | -4.16 |
| 1558397_at | --- | CDNA FLJ34100 fis, clone FCBBF3007597 | BF976693 | -4.16 |
| 201613_s_at | AP1G2 | adaptor-related protein complex 1, gamma 2 subunit | BC000519 | -4.22 |
| 212547_at | FLJ35348 /// BRD3 | FLJ35348 /// Bromodomain containing 3 | N34842 | -4.37 |
| 210822_at | LOC283345 | RPL13-2 pseudogene | U72513 | -4.44 |
| 224726_at | MIB1 | mindbomb homolog 1 (Drosophila) | W80418 | -4.67 |
| 218051_s_at | FLJ12442 | hypothetical protein FLJ12442 | NM_022908 | -4.78 |
| 203979_at | CYP27A1 | cytochrome P450, family 27, subfamily A, polypeptide 1 | NM_000784 | -4.81 |
| 207556_s_at | DGKZ | diacylglycerol kinase, zeta 104kDa | NM_003646 | -4.82 |
| 206028_s_at | MERTK | c-mer proto-oncogene tyrosine kinase | NM_006343 | -4.93 |
| 202838_at | FUCA1 | fucosidase, alpha-L- 1, tissue | NM_000147 | -9.62 |
