# Supplementary material for: Blockade of T Cell Contact-Activation of Human Monocytes by High-Density Lipoproteins Reveals a New Pattern of Cytokine and Inflammatory Genes
Source: PLoS One. 2010 Feb 25;5(2):e9418. doi: 10.1371/journal.pone.0009418 (PMC2828473; doi:10.1371/journal.pone.0009418)
Supplement: Table S2 — Probe sets whose signal was affected in the presence of CEsHUT and HDL. (1.76 MB DOC) [file pone.0009418.s002.doc]

# Table S2: Probe sets whose signal was affected in the presence of CE_sHUT_ and HDL

| **Affymetrix ID** | **Gene Symbol** | **Description** | **Representative ID** | **Fold Change** |
| --- | --- | --- | --- | --- |
| **230067_at** | --- | Transcribed locus | AA151659 | 62.08 |
| **238439_at** | ANKRD22 | ankyrin repeat domain 22 | AI925518 | 59.51 |
| **227140_at** | INHBA | Inhibin, beta A (activin A, activin AB alpha polypeptide) | AI343467 | 36.91 |
| **210511_s_at** | INHBA | inhibin, beta A (activin A, activin AB alpha polypeptide) | M13436 | 22.96 |
| **204533_at** | CXCL10 | chemokine (C-X-C motif) ligand 10 | NM_001565 | 22.31 |
| **204438_at** | MRC1 /// MRC1L1 | mannose receptor, C type 1 /// mannose receptor, C type 1-like 1 | NM_002438 | 21.70 |
| **221266_s_at** | TM7SF4 | transmembrane 7 superfamily member 4 /// transmembrane 7 superfamily member 4 | NM_030788 | 20.55 |
| **212946_at** | KIAA0564 | KIAA0564 protein | AK025432 | 20.14 |
| **229625_at** | GBP5 | Guanylate binding protein 5 | BG545653 | 19.76 |
| **204363_at** | F3 | coagulation factor III (thromboplastin, tissue factor) | NM_001993 | 17.76 |
| **238581_at** | GBP5 | Guanylate binding protein 5 | BG271923 | 17.71 |
| **228186_s_at** | RSPO3 | R-spondin 3 homolog (Xenopus laevis) | BF589322 | 17.04 |
| **226498_at** | FLT1 | Fms-related tyrosine kinase 1 (vascular endothelial growth factor/vascular permeability factor receptor) | AA149648 | 15.10 |
| **226497_s_at** | FLT1 | Fms-related tyrosine kinase 1 (vascular endothelial growth factor/vascular permeability factor receptor) | AA149648 | 11.86 |
| **219385_at** | SLAMF8 | SLAM family member 8 | NM_020125 | 11.78 |
| **204011_at** | SPRY2 | sprouty homolog 2 (Drosophila) | NM_005842 | 11.58 |
| **219386_s_at** | SLAMF8 | SLAM family member 8 | NM_020125 | 11.54 |
| **239196_at** | ANKRD22 | ankyrin repeat domain 22 | AI097229 | 11.09 |
| **213895_at** | EMP1 | epithelial membrane protein 1 | BF445047 | 11.05 |
| **231578_at** | GBP1 /// LOC400759 | guanylate binding protein 1, interferon-inducible, 67kDa /// similar to Interferon-induced guanylate-binding protein 1 (GTP-binding protein 1) (Guanine nucleotide-binding protein 1) (HuGBP-1) | AW014593 | 10.45 |
| **AFFX-HUMRGE/M10098_3_at** | SRP68 | signal recognition particle 68kDa | AFFX-HUMRGE/M10098_3 | 10.18 |
| **209969_s_at** | STAT1 | signal transducer and activator of transcription 1, 91kDa | BC002704 | 9.69 |
| **AFFX-HUMISGF3A/M97935_MB_at** | STAT1 | signal transducer and activator of transcription 1, 91kDa | AFFX-HUMISGF3A/M97935_MB | 9.54 |
| **241869_at** | APOL6 | apolipoprotein L, 6 | AW026509 | 9.44 |
| **201325_s_at** | EMP1 | epithelial membrane protein 1 | NM_001423 | 9.18 |
| **205249_at** | EGR2 | early growth response 2 (Krox-20 homolog, Drosophila) | NM_000399 | 9.16 |
| **223939_at** | SUCNR1 | succinate receptor 1 | AF348078 | 8.63 |
| **210517_s_at** | AKAP12 | A kinase (PRKA) anchor protein (gravin) 12 | AB003476 | 8.47 |
| **203915_at** | CXCL9 | chemokine (C-X-C motif) ligand 9 | NM_002416 | 8.34 |
| **236898_at** | --- | Transcribed locus | AW242604 | 8.30 |
| **202270_at** | GBP1 | guanylate binding protein 1, interferon-inducible, 67kDa /// guanylate binding protein 1, interferon-inducible, 67kDa | NM_002053 | 8.16 |
| **231577_s_at** | GBP1 | guanylate binding protein 1, interferon-inducible, 67kDa | AW014593 | 7.56 |
| **AFFX-HUMISGF3A/M97935_5_at** | STAT1 | signal transducer and activator of transcription 1, 91kDa | AFFX-HUMISGF3A/M97935_5 | 7.33 |
| **227529_s_at** | AKAP12 | A kinase (PRKA) anchor protein (gravin) 12 | BF511276 | 7.27 |
| **236606_at** | SAV1 | Salvador homolog 1 (Drosophila) | N50912 | 7.08 |
| **230519_at** | FLJ30707 | hypothetical protein FLJ30707 | D59502 | 7.07 |
| **227034_at** | C2orf26 | chromosome 2 open reading frame 26 | BE669553 | 6.99 |
| **38037_at** | HBEGF | heparin-binding EGF-like growth factor | M60278 | 6.93 |
| **39248_at** | AQP3 | aquaporin 3 | N74607 | 6.53 |
| **221085_at** | TNFSF15 | tumor necrosis factor (ligand) superfamily, member 15 | NM_005118 | 6.52 |
| **1554841_at** | MTHFD2L | Methylenetetrahydrofolate dehydrogenase (NADP+ dependent) 2-like | BC032771 | 6.48 |
| **228242_at** | --- | Transcribed locus | BF055201 | 6.47 |
| **1563445_x_at** | CTSLL3 | cathepsin L-like 3 | L25629 | 6.34 |
| **1568592_at** | LOC400368 | hypothetical gene supported by BC031266 | BM976092 | 6.30 |
| **200887_s_at** | STAT1 | signal transducer and activator of transcription 1, 91kDa | NM_007315 | 6.30 |
| **227458_at** | PDCD1LG1 | CD274 antigen | AI608902 | 6.27 |
| **1559777_at** | --- | CDNA FLJ32866 fis, clone TESTI2003718 | AK057428 | 6.25 |
| **236140_at** | GCLM | glutamate-cysteine ligase, modifier subunit | AI753488 | 6.22 |
| **202269_x_at** | GBP1 | guanylate binding protein 1, interferon-inducible, 67kDa /// guanylate binding protein 1, interferon-inducible, 67kDa | BC002666 | 6.11 |
| **203821_at** | HBEGF | heparin-binding EGF-like growth factor | NM_001945 | 5.95 |
| **1564796_at** | EMP1 | epithelial membrane protein 1 | BC017854 | 5.86 |
| **226474_at** | NOD27 | nucleotide-binding oligomerization domains 27 | AA005023 | 5.84 |
| **204926_at** | INHBA | inhibin, beta A (activin A, activin AB alpha polypeptide) | NM_002192 | 5.82 |
| **232375_at** | STAT1 | Signal transducer and activator of transcription 1, 91kDa | AI539443 | 5.77 |
| **220253_s_at** | LRP12 | low density lipoprotein-related protein 12 | NM_013437 | 5.71 |
| **209324_s_at** | RGS16 | regulator of G-protein signalling 16 | BF304996 | 5.55 |
| **231972_at** | --- | Full length insert cDNA clone ZE08A03 | AK024681 | 5.53 |
| **205227_at** | IL1RAP | interleukin 1 receptor accessory protein | NM_002182 | 5.50 |
| **201324_at** | EMP1 | epithelial membrane protein 1 | NM_001423 | 5.48 |
| **1569095_at** | --- | Homo sapiens, clone IMAGE:4133286, mRNA | BC016366 | 5.42 |
| **229390_at** | LOC441168 | hypothetical protein LOC441168 | AV734646 | 5.35 |
| **220745_at** | IL19 | interleukin 19 | NM_013371 | 5.31 |
| **231996_at** | N4BP2 | Nedd4 binding protein 2 | AB037834 | 5.28 |
| **219716_at** | APOL6 | apolipoprotein L, 6 | NM_030641 | 5.26 |
| **227530_at** | AKAP12 | A kinase (PRKA) anchor protein (gravin) 12 | BF511276 | 5.26 |
| **219496_at** | C2orf26 | chromosome 2 open reading frame 26 | NM_023016 | 5.26 |
| **234491_s_at** | SAV1 | salvador homolog 1 (Drosophila) | AJ292969 | 5.24 |
| **204803_s_at** | RRAD | Ras-related associated with diabetes | NM_004165 | 5.20 |
| **207113_s_at** | TNF | tumor necrosis factor (TNF superfamily, member 2) | NM_000594 | 5.19 |
| **202307_s_at** | TAP1 | transporter 1, ATP-binding cassette, sub-family B (MDR/TAP) | NM_000593 | 5.16 |
| **210139_s_at** | PMP22 | peripheral myelin protein 22 | L03203 | 5.13 |
| **229011_at** | EMP1 | Epithelial membrane protein 1 | AA150501 | 5.12 |
| **235574_at** | GBP4 | guanylate binding protein 4 | AW392952 | 5.10 |
| **220346_at** | MTHFD2L | methylenetetrahydrofolate dehydrogenase (NADP+ dependent) 2-like | NM_025001 | 5.10 |
| **240287_at** | LOC341720 | similar to immune-responsive gene 1 | BG236136 | 5.06 |
| **1557236_at** | --- | --- | BF512806 | 5.03 |
| **209706_at** | NKX3-1 | NK3 transcription factor related, locus 1 (Drosophila) | AF247704 | 5.03 |
| **1562102_at** | AKR1C1 | Aldo-keto reductase family 1, member C1 (dihydrodiol dehydrogenase 1; 20-alpha (3-alpha)-hydroxysteroid dehydrogenase) | BC014579 | 5.01 |
| **209457_at** | DUSP5 | dual specificity phosphatase 5 | U16996 | 4.94 |
| **203725_at** | GADD45A | growth arrest and DNA-damage-inducible, alpha | NM_001924 | 4.85 |
| **231576_at** | --- | MRNA; cDNA DKFZp566C034 (from clone DKFZp566C034) | AA829940 | 4.84 |
| **219412_at** | RAB38 | RAB38, member RAS oncogene family | NM_022337 | 4.82 |
| **238725_at** | --- | Transcribed locus, weakly similar to XP_496299.1 PREDICTED: hypothetical protein LOC148206 [Homo sapiens] | AW392551 | 4.81 |
| **230741_at** | --- | CDNA FLJ41454 fis, clone BRSTN2011597 | AI655467 | 4.80 |
| **217933_s_at** | LAP3 | leucine aminopeptidase 3 | NM_015907 | 4.79 |
| **234986_at** | GCLM | Glutamate-cysteine ligase, modifier subunit | AA630626 | 4.79 |
| **1558517_s_at** | --- | CDNA FLJ37485 fis, clone BRAWH2014379 | CA773938 | 4.70 |
| **219622_at** | RAB20 | RAB20, member RAS oncogene family | NM_017817 | 4.65 |
| **1555960_at** | HINT1 | Histidine triad nucleotide binding protein 1 | AK054976 | 4.60 |
| **206995_x_at** | SCARF1 | scavenger receptor class F, member 1 | NM_003693 | 4.60 |
| **209099_x_at** | JAG1 | jagged 1 (Alagille syndrome) | U73936 | 4.50 |
| **226893_at** | ABL2 | V-abl Abelson murine leukemia viral oncogene homolog 2 (arg, Abelson-related gene) | AW173164 | 4.50 |
| **202531_at** | IRF1 | interferon regulatory factor 1 | NM_002198 | 4.49 |
| **238729_x_at** | SAV1 | Salvador homolog 1 (Drosophila) | BF983202 | 4.48 |
| **225283_at** | ARRDC4 | arrestin domain containing 4 | AV701177 | 4.48 |
| **211653_x_at** | AKR1C2 | aldo-keto reductase family 1, member C2 (dihydrodiol dehydrogenase 2; bile acid binding protein; 3-alpha hydroxysteroid dehydrogenase, type III) /// aldo-keto reductase family 1, member C2 (dihydrodiol dehydrogenase 2; bile acid binding protein; 3-alpha hydroxysteroid dehydrogenase, type III) | M33376 | 4.46 |
| **201328_at** | ETS2 | v-ets erythroblastosis virus E26 oncogene homolog 2 (avian) | AL575509 | 4.44 |
| **201431_s_at** | DPYSL3 | dihydropyrimidinase-like 3 | NM_001387 | 4.43 |
| **204286_s_at** | PMAIP1 | phorbol-12-myristate-13-acetate-induced protein 1 | NM_021127 | 4.37 |
| **201243_s_at** | ATP1B1 | ATPase, Na+/K+ transporting, beta 1 polypeptide | NM_001677 | 4.36 |
| **209210_s_at** | PLEKHC1 | pleckstrin homology domain containing, family C (with FERM domain) member 1 | Z24725 | 4.36 |
| **220865_s_at** | TPRT | trans-prenyltransferase | NM_014317 | 4.28 |
| **216594_x_at** | AKR1C1 | aldo-keto reductase family 1, member C1 (dihydrodiol dehydrogenase 1; 20-alpha (3-alpha)-hydroxysteroid dehydrogenase) | S68290 | 4.26 |
| **212500_at** | C10orf22 | chromosome 10 open reading frame 22 | AL049319 | 4.25 |
| **231899_at** | ZC3H12C | zinc finger CCCH-type containing 12C | AB051513 | 4.24 |
| **1559203_s_at** | KRAS | v-Ki-ras2 Kirsten rat sarcoma viral oncogene homolog | BC029545 | 4.23 |
| **1559051_s_at** | C6orf150 | chromosome 6 open reading frame 150 | AK097148 | 4.20 |
| **228314_at** | --- | CDNA FLJ37485 fis, clone BRAWH2014379 | BE877357 | 4.19 |
| **228033_at** | E2F7 | E2F transcription factor 7 | AI341146 | 4.15 |
| **218276_s_at** | SAV1 | salvador homolog 1 (Drosophila) | NM_021818 | 4.14 |
| **218681_s_at** | SDF2L1 | stromal cell-derived factor 2-like 1 | NM_022044 | 4.14 |
| **223533_at** | LRRC8C | leucine rich repeat containing 8 family, member C | AL136919 | 4.14 |
| **230511_at** | CREM | cAMP responsive element modulator | AI800640 | 4.11 |
| **222573_s_at** | SAV1 | salvador homolog 1 (Drosophila) | AI679398 | 4.10 |
| **222870_s_at** | B3GNT1 | UDP-GlcNAc:betaGal beta-1,3-N-acetylglucosaminyltransferase 1 | AF288208 | 4.07 |
| **240421_x_at** | --- | MRNA; cDNA DKFZp313N2115 (from clone DKFZp313N2115) | AI693524 | 4.04 |
| **223434_at** | GBP3 | guanylate binding protein 3 | AL136680 | 4.04 |
| **222139_at** | KIAA1466 | KIAA1466 gene | AI765383 | 4.02 |
| **229391_s_at** | LOC441168 | hypothetical protein LOC441168 | AV734646 | 4.01 |
| **216268_s_at** | JAG1 | jagged 1 (Alagille syndrome) | U77914 | 3.99 |
| **206148_at** | IL3RA | interleukin 3 receptor, alpha (low affinity) | NM_002183 | 3.98 |
| **212614_at** | ARID5B | AT rich interactive domain 5B (MRF1-like) | BG285011 | 3.98 |
| **209544_at** | RIPK2 | receptor-interacting serine-threonine kinase 2 | AF027706 | 3.97 |
| **204279_at** | PSMB9 | proteasome (prosome, macropain) subunit, beta type, 9 (large multifunctional peptidase 2) | NM_002800 | 3.95 |
| **211445_x_at** | NACAP1 /// LOC389240 | nascent-polypeptide-associated complex alpha polypeptide pseudogene 1 /// similar to alpha NAC/1.9.2. protein | AF315951 | 3.94 |
| **228366_at** | PPA2 | pyrophosphatase (inorganic) 2 | BF446912 | 3.92 |
| **222402_at** | C13orf12 | chromosome 13 open reading frame 12 | BC003390 | 3.90 |
| **200628_s_at** | WARS | tryptophanyl-tRNA synthetase | M61715 | 3.90 |
| **235175_at** | GBP4 | guanylate binding protein 4 | BG260886 | 3.89 |
| **203276_at** | LMNB1 | lamin B1 | NM_005573 | 3.89 |
| **215252_at** | DNAJC7 | DnaJ (Hsp40) homolog, subfamily C, member 7 | AW814026 | 3.86 |
| **1564274_at** | C9orf47 | chromosome 9 open reading frame 47 | AK094842 | 3.85 |
| **226099_at** | ELL2 | elongation factor, RNA polymerase II, 2 | AI924426 | 3.83 |
| **229307_at** | ANKRD28 | ankyrin repeat domain 28 | N32051 | 3.78 |
| **231292_at** | EID3 | E1A-like inhibitor of differentiation 3 | AI964053 | 3.78 |
| **238013_at** | PLEKHA2 | pleckstrin homology domain containing, family A (phosphoinositide binding specific) member 2 | BF347859 | 3.78 |
| **1557120_at** | --- | CDNA clone MGC:61837 IMAGE:4809394 | BE622780 | 3.77 |
| **201430_s_at** | DPYSL3 | dihydropyrimidinase-like 3 | W72516 | 3.76 |
| **212698_s_at** | 10.sept | septin 10 | BF966021 | 3.74 |
| **201649_at** | UBE2L6 | ubiquitin-conjugating enzyme E2L 6 | NM_004223 | 3.73 |
| **226921_at** | UBR1 | Ubiquitin protein ligase E3 component n-recognin 1 | AV715153 | 3.72 |
| **209795_at** | CD69 | CD69 antigen (p60, early T-cell activation antigen) | L07555 | 3.71 |
| **201466_s_at** | JUN | v-jun sarcoma virus 17 oncogene homolog (avian) | NM_002228 | 3.69 |
| **210074_at** | CTSL2 | cathepsin L2 | AF070448 | 3.69 |
| **216236_s_at** | SLC2A3 /// SLC2A14 | solute carrier family 2 (facilitated glucose transporter), member 3 /// solute carrier family 2 (facilitated glucose transporter), member 14 | AL110298 | 3.67 |
| **210896_s_at** | ASPH | aspartate beta-hydroxylase | AF306765 | 3.65 |
| **211702_s_at** | USP32 | ubiquitin specific peptidase 32 /// ubiquitin specific peptidase 32 | AF350251 | 3.64 |
| **201060_x_at** | STOM | stomatin | AI537887 | 3.62 |
| **208510_s_at** | PPARG | peroxisome proliferative activated receptor, gamma | NM_015869 | 3.61 |
| **229242_at** | --- | Transcribed locus | BF439063 | 3.56 |
| **202131_s_at** | RIOK3 | RIO kinase 3 (yeast) /// RIO kinase 3 (yeast) | NM_003831 | 3.55 |
| **201436_at** | EIF4E | eukaryotic translation initiation factor 4E | AI742789 | 3.53 |
| **1557197_a_at** | LGALS3 | Lectin, galactoside-binding, soluble, 3 (galectin 3) | AW085690 | 3.52 |
| **236313_at** | CDKN2B | cyclin-dependent kinase inhibitor 2B (p15, inhibits CDK4) | AW444761 | 3.52 |
| **223068_at** | EML4 | echinoderm microtubule associated protein like 4 | AV707345 | 3.50 |
| **220104_at** | ZC3HAV1 | zinc finger CCCH-type, antiviral 1 | NM_020119 | 3.50 |
| **201329_s_at** | ETS2 | v-ets erythroblastosis virus E26 oncogene homolog 2 (avian) | NM_005239 | 3.48 |
| **218999_at** | FLJ11000 | hypothetical protein FLJ11000 | NM_018295 | 3.46 |
| **213338_at** | RIS1 | Ras-induced senescence 1 | BF062629 | 3.46 |
| **1555854_at** | --- | CDNA FLJ33809 fis, clone CTONG2001858 | AA594609 | 3.45 |
| **200733_s_at** | PTP4A1 | protein tyrosine phosphatase type IVA, member 1 | U48296 | 3.43 |
| **202988_s_at** | RGS1 | regulator of G-protein signalling 1 | NM_002922 | 3.43 |
| **1563469_at** | ARID5B | AT rich interactive domain 5B (MRF1-like) | AL832681 | 3.43 |
| **236198_at** | --- | Transcribed locus | AW292872 | 3.42 |
| **225047_at** | NUPL1 | nucleoporin like 1 | AF113020 | 3.42 |
| **209135_at** | ASPH | aspartate beta-hydroxylase | AF289489 | 3.41 |
| **202498_s_at** | SLC2A3 | solute carrier family 2 (facilitated glucose transporter), member 3 | BE550486 | 3.40 |
| **242727_at** | ARL8 | ADP-ribosylation factor-like 8 | BG032269 | 3.40 |
| **200730_s_at** | PTP4A1 | protein tyrosine phosphatase type IVA, member 1 | BF576710 | 3.39 |
| **202499_s_at** | SLC2A3 | solute carrier family 2 (facilitated glucose transporter), member 3 | NM_006931 | 3.39 |
| **226844_at** | MOBKL2B | MOB1, Mps One Binder kinase activator-like 2B (yeast) | AI375115 | 3.36 |
| **220386_s_at** | EML4 | echinoderm microtubule associated protein like 4 | NM_019063 | 3.36 |
| **219631_at** | LRP12 | low density lipoprotein-related protein 12 | NM_024937 | 3.35 |
| **226117_at** | TIFA | TRAF-interacting protein with a forkhead-associated domain | AA195074 | 3.35 |
| **202672_s_at** | ATF3 | activating transcription factor 3 | NM_001674 | 3.34 |
| **205016_at** | TGFA | transforming growth factor, alpha | NM_003236 | 3.34 |
| **203925_at** | GCLM | glutamate-cysteine ligase, modifier subunit | NM_002061 | 3.33 |
| **203108_at** | GPRC5A | G protein-coupled receptor, family C, group 5, member A | NM_003979 | 3.33 |
| **201437_s_at** | EIF4E | eukaryotic translation initiation factor 4E | NM_001968 | 3.32 |
| **206569_at** | IL24 | interleukin 24 | NM_006850 | 3.30 |
| **223834_at** | CD274 | CD274 antigen | AF233516 | 3.30 |
| **1555247_a_at** | RAPGEF6 | Rap guanine nucleotide exchange factor (GEF) 6 | AF394782 | 3.30 |
| **216199_s_at** | MAP3K4 | mitogen-activated protein kinase kinase kinase 4 | AL109942 | 3.29 |
| **204089_x_at** | MAP3K4 | mitogen-activated protein kinase kinase kinase 4 | NM_006724 | 3.29 |
| **242907_at** | GBP2 | guanylate binding protein 2, interferon-inducible | BF509371 | 3.28 |
| **235157_at** | PARP14 | Poly (ADP-ribose) polymerase family, member 14 | AW297731 | 3.26 |
| **209765_at** | ADAM19 | ADAM metallopeptidase domain 19 (meltrin beta) | Y13786 | 3.25 |
| **201464_x_at** | JUN | v-jun sarcoma virus 17 oncogene homolog (avian) | BG491844 | 3.24 |
| **226603_at** | SAMD9L | sterile alpha motif domain containing 9-like | BE966604 | 3.23 |
| **215236_s_at** | PICALM | phosphatidylinositol binding clathrin assembly protein | AV721177 | 3.23 |
| **242388_x_at** | TAGAP | T-cell activation GTPase activating protein | AW576600 | 3.23 |
| **202759_s_at** | PALM2-AKAP2 | PALM2-AKAP2 protein | BE879367 | 3.22 |
| **201435_s_at** | EIF4E | eukaryotic translation initiation factor 4E | AW268640 | 3.21 |
| **206115_at** | EGR3 | early growth response 3 | NM_004430 | 3.21 |
| **203810_at** | DNAJB4 | DnaJ (Hsp40) homolog, subfamily B, member 4 | BG252490 | 3.20 |
| **213872_at** | C6orf62 | Chromosome 6 open reading frame 62 | BE465032 | 3.20 |
| **203840_at** | BLZF1 | basic leucine zipper nuclear factor 1 (JEM-1) | NM_003666 | 3.19 |
| **237032_x_at** | LOC283567 | hypothetical protein LOC283567 | BG055441 | 3.18 |
| **1552612_at** | CDC42SE2 | CDC42 small effector 2 | NM_020240 | 3.17 |
| **241068_at** | DREV1 | DORA reverse strand protein 1 | BF510881 | 3.15 |
| **202497_x_at** | SLC2A3 | solute carrier family 2 (facilitated glucose transporter), member 3 | AI631159 | 3.15 |
| **1552613_s_at** | CDC42SE2 | CDC42 small effector 2 | NM_020240 | 3.15 |
| **228167_at** | KLHL6 | kelch-like 6 (Drosophila) | AW574798 | 3.14 |
| **228231_at** | SNX8 | Sorting nexin 8 | BE221804 | 3.14 |
| **216316_x_at** | --- | PREDICTED: Homo sapiens similar to GKP3 protein (LOC201989), mRNA | X78713 | 3.14 |
| **242312_x_at** | --- | Transcribed locus | AV736963 | 3.13 |
| **210340_s_at** | CSF2RA | colony stimulating factor 2 receptor, alpha, low-affinity (granulocyte-macrophage) | BC002635 | 3.13 |
| **203665_at** | HMOX1 | heme oxygenase (decycling) 1 | NM_002133 | 3.12 |
| **226025_at** | ANKRD28 | ankyrin repeat domain 28 | AV740426 | 3.12 |
| **204977_at** | DDX10 | DEAD (Asp-Glu-Ala-Asp) box polypeptide 10 | NM_004398 | 3.12 |
| **209226_s_at** | TNPO1 | transportin 1 | U72069 | 3.11 |
| **1559204_x_at** | KRAS | v-Ki-ras2 Kirsten rat sarcoma viral oncogene homolog | BC029545 | 3.11 |
| **201279_s_at** | DAB2 | disabled homolog 2, mitogen-responsive phosphoprotein (Drosophila) | BC003064 | 3.11 |
| **205745_x_at** | ADAM17 | ADAM metallopeptidase domain 17 (tumor necrosis factor, alpha, converting enzyme) | NM_003183 | 3.09 |
| **221211_s_at** | C21orf7 | chromosome 21 open reading frame 7 | NM_020152 | 3.09 |
| **223220_s_at** | PARP9 | poly (ADP-ribose) polymerase family, member 9 | AF307338 | 3.09 |
| **235711_at** | PURB | purine-rich element binding protein B | AA495775 | 3.08 |
| **1552542_s_at** | TAGAP | T-cell activation GTPase activating protein | NM_138810 | 3.08 |
| **217752_s_at** | CNDP2 | CNDP dipeptidase 2 (metallopeptidase M20 family) | NM_018235 | 3.07 |
| **241887_at** | --- | CDNA FLJ41537 fis, clone BRTHA2017985 | AI370381 | 3.05 |
| **225636_at** | STAT2 | signal transducer and activator of transcription 2, 113kDa | H98105 | 3.03 |
| **201468_s_at** | NQO1 | NAD(P)H dehydrogenase, quinone 1 | NM_000903 | 3.03 |
| **235816_s_at** | Rgr | Ral-GDS related protein Rgr | AI867408 | 3.03 |
| **222846_at** | RAB8B | RAB8B, member RAS oncogene family | AB038995 | 3.03 |
| **1570007_at** | FAD158 | Leucine rich repeat containing 8 family, member C | BC036122 | 3.03 |
| **231956_at** | KIAA1618 | KIAA1618 | AA976354 | 3.02 |
| **205269_at** | LCP2 | lymphocyte cytosolic protein 2 (SH2 domain containing leukocyte protein of 76kDa) | AI123251 | 3.02 |
| **218647_s_at** | YRDC | yrdC domain containing (E.coli) | NM_024640 | 3.01 |
| **228176_at** | EDG3 | endothelial differentiation, sphingolipid G-protein-coupled receptor, 3 | AA534817 | 3.01 |
| **226694_at** | PALM2-AKAP2 | PALM2-AKAP2 protein | BG540494 | 3.00 |
| **218145_at** | TRIB3 | tribbles homolog 3 (Drosophila) | NM_021158 | 2.99 |
| **1557116_at** | --- | --- | BM980001 | 2.99 |
| **201363_s_at** | IVNS1ABP | influenza virus NS1A binding protein | AB020657 | 2.97 |
| **209803_s_at** | PHLDA2 | pleckstrin homology-like domain, family A, member 2 | AF001294 | 2.97 |
| **216609_at** | TXN | Thioredoxin | AF065241 | 2.95 |
| **228468_at** | MASTL | microtubule associated serine/threonine kinase-like | BF108964 | 2.95 |
| **1561908_a_at** | HS3ST3B1 | Hypothetical protein MGC12916 | AL832823 | 2.95 |
| **244026_at** | ELL2 | Elongation factor, RNA polymerase II, 2 | BF063657 | 2.94 |
| **208864_s_at** | TXN | thioredoxin | AF313911 | 2.94 |
| **221510_s_at** | GLS | glutaminase | AF158555 | 2.93 |
| **225252_at** | SRXN1 | sulfiredoxin 1 homolog (S. cerevisiae) | AL121758 | 2.92 |
| **202823_at** | TCEB1 | transcription elongation factor B (SIII), polypeptide 1 (15kDa, elongin C) | N89607 | 2.92 |
| **229718_at** | --- | CDNA clone IMAGE:4111575 | BF448287 | 2.92 |
| **205239_at** | AREG | amphiregulin (schwannoma-derived growth factor) | NM_001657 | 2.92 |
| **229437_at** | BIC | BIC transcript | BG231961 | 2.92 |
| **210118_s_at** | IL1A | interleukin 1, alpha | M15329 | 2.92 |
| **209193_at** | PIM1 | pim-1 oncogene /// pim-1 oncogene | M24779 | 2.91 |
| **239258_at** | RHOQ | Ras homolog gene family, member Q | BE551407 | 2.91 |
| **209122_at** | ADFP | adipose differentiation-related protein | BC005127 | 2.91 |
| **214329_x_at** | TNFSF10 | Tumor necrosis factor (ligand) superfamily, member 10 /// Tumor necrosis factor (ligand) superfamily, member 10 | AW474434 | 2.90 |
| **217599_s_at** | MDFIC | MyoD family inhibitor domain containing | BE910600 | 2.89 |
| **211924_s_at** | PLAUR | plasminogen activator, urokinase receptor /// plasminogen activator, urokinase receptor | AY029180 | 2.89 |
| **204748_at** | PTGS2 | prostaglandin-endoperoxide synthase 2 (prostaglandin G/H synthase and cyclooxygenase) | NM_000963 | 2.89 |
| **204151_x_at** | AKR1C1 | aldo-keto reductase family 1, member C1 (dihydrodiol dehydrogenase 1; 20-alpha (3-alpha)-hydroxysteroid dehydrogenase) | NM_001353 | 2.89 |
| **207850_at** | CXCL3 | chemokine (C-X-C motif) ligand 3 | NM_002090 | 2.89 |
| **210519_s_at** | NQO1 | NAD(P)H dehydrogenase, quinone 1 | BC000906 | 2.89 |
| **203427_at** | ASF1A | ASF1 anti-silencing function 1 homolog A (S. cerevisiae) | NM_014034 | 2.88 |
| **231270_at** | CA13 | carbonic anhydrase XIII | BF111998 | 2.88 |
| **227947_at** | PHACTR2 | phosphatase and actin regulator 2 | AV724107 | 2.88 |
| **230233_at** | RASGEF1B | RasGEF domain family, member 1B | BF110534 | 2.87 |
| **231769_at** | FBXO6 | F-box protein 6 | AF129536 | 2.87 |
| **226390_at** | STARD4 | START domain containing 4, sterol regulated | AA628398 | 2.87 |
| **224701_at** | PARP14 | poly (ADP-ribose) polymerase family, member 14 | AA056548 | 2.87 |
| **232829_at** | OR52K3P | olfactory receptor, family 52, subfamily K, member 3 pseudogene | AF143328 | 2.87 |
| **1569004_at** | --- | CDNA FLJ43560 fis, clone PUAEN2000175 | BG541677 | 2.87 |
| **222309_at** | C6orf62 | Chromosome 6 open reading frame 62 | AW972292 | 2.86 |
| **217371_s_at** | IL15 | interleukin 15 | Y09908 | 2.86 |
| **226541_at** | FBXO30 | F-box protein 30 | AI808182 | 2.85 |
| **203479_s_at** | OTUD4 | OTU domain containing 4 | T79216 | 2.85 |
| **204802_at** | RRAD | Ras-related associated with diabetes | NM_004165 | 2.85 |
| **201131_s_at** | CDH1 | cadherin 1, type 1, E-cadherin (epithelial) | NM_004360 | 2.85 |
| **239835_at** | KBTBD8 | kelch repeat and BTB (POZ) domain containing 8 | AA669114 | 2.85 |
| **211985_s_at** | CALM1 | calmodulin 1 (phosphorylase kinase, delta) | AI653730 | 2.84 |
| **215910_s_at** | FNDC3A | fibronectin type III domain containing 3A | AL137000 | 2.84 |
| **231832_at** | GALNT4 | UDP-N-acetyl-alpha-D-galactosamine:polypeptide N-acetylgalactosaminyltransferase 4 (GalNAc-T4) | AI890347 | 2.84 |
| **219684_at** | IFRG28 | 28kD interferon responsive protein | NM_022147 | 2.83 |
| **221087_s_at** | APOL3 | apolipoprotein L, 3 | NM_014349 | 2.83 |
| **214438_at** | HLX1 | H2.0-like homeo box 1 (Drosophila) | M60721 | 2.83 |
| **209585_s_at** | MINPP1 | multiple inositol polyphosphate histidine phosphatase, 1 | AF084943 | 2.83 |
| **235204_at** | COX15 | COX15 homolog, cytochrome c oxidase assembly protein (yeast) | BF878343 | 2.81 |
| **244322_at** | --- | CDNA FLJ38230 fis, clone FCBBF2004448 | AW362008 | 2.81 |
| **225646_at** | CTSC | cathepsin C | AI246687 | 2.81 |
| **222553_x_at** | OXR1 | oxidation resistance 1 | AL541048 | 2.81 |
| **1554237_at** | SDCCAG8 | serologically defined colon cancer antigen 8 | BC032454 | 2.80 |
| **207630_s_at** | CREM | cAMP responsive element modulator | NM_001881 | 2.79 |
| **222088_s_at** | SLC2A3 /// SLC2A14 | solute carrier family 2 (facilitated glucose transporter), member 3 /// solute carrier family 2 (facilitated glucose transporter), member 14 | AA778684 | 2.79 |
| **225973_at** | TAP2 | transporter 2, ATP-binding cassette, sub-family B (MDR/TAP) | AA573502 | 2.79 |
| **1558836_at** | --- | MRNA; cDNA DKFZp667A182 (from clone DKFZp667A182) | BQ024490 | 2.78 |
| **1559585_at** | --- | --- | AK096369 | 2.78 |
| **202907_s_at** | NBN | nibrin | NM_002485 | 2.78 |
| **212298_at** | NRP1 | neuropilin 1 | BE620457 | 2.78 |
| **239562_at** | MTHFD2L | methylenetetrahydrofolate dehydrogenase (NADP+ dependent) 2-like | AW272411 | 2.77 |
| **201089_at** | ATP6V1B2 | ATPase, H+ transporting, lysosomal 56/58kDa, V1 subunit B, isoform 2 | NM_001693 | 2.77 |
| **213638_at** | PHACTR1 | phosphatase and actin regulator 1 | AW054711 | 2.77 |
| **207339_s_at** | LTB | lymphotoxin beta (TNF superfamily, member 3) | NM_002341 | 2.77 |
| **32088_at** | BLZF1 | basic leucine zipper nuclear factor 1 (JEM-1) | U79751 | 2.76 |
| **209875_s_at** | SPP1 | secreted phosphoprotein 1 (osteopontin, bone sialoprotein I, early T-lymphocyte activation 1) | M83248 | 2.75 |
| **1560069_at** | LOC389072 | hypothetical protein LOC389072 | BC020812 | 2.75 |
| **223461_at** | TBC1D7 | TBC1 domain family, member 7 | AF151073 | 2.74 |
| **203543_s_at** | KLF9 | Kruppel-like factor 9 | NM_001206 | 2.74 |
| **224593_at** | ZNF664 | zinc finger protein 664 | BE965646 | 2.74 |
| **213355_at** | ST3GAL6 | ST3 beta-galactoside alpha-2,3-sialyltransferase 6 | AI989567 | 2.74 |
| **241396_at** | NEDD4L | neural precursor cell expressed, developmentally down-regulated 4-like | BG540188 | 2.73 |
| **213618_at** | CENTD1 | centaurin, delta 1 | AB011152 | 2.73 |
| **229026_at** | CDC42SE2 | CDC42 small effector 2 | BE675995 | 2.72 |
| **214590_s_at** | UBE2D1 | ubiquitin-conjugating enzyme E2D 1 (UBC4/5 homolog, yeast) | AL545760 | 2.72 |
| **225580_at** | MRPL50 | mitochondrial ribosomal protein L50 | BG028213 | 2.71 |
| **227391_x_at** | LRRFIP1 | leucine rich repeat (in FLII) interacting protein 1 | BE674143 | 2.71 |
| **224595_at** | SLC44A1 | solute carrier family 44, member 1 | AK022549 | 2.71 |
| **237718_at** | EIF4E | eukaryotic translation initiation factor 4E | AA913840 | 2.71 |
| **231907_at** | ABL2 | V-abl Abelson murine leukemia viral oncogene homolog 2 (arg, Abelson-related gene) | AK025877 | 2.70 |
| **1568768_s_at** | BRE | brain and reproductive organ-expressed (TNFRSF1A modulator) | AW080339 | 2.70 |
| **225647_s_at** | CTSC | cathepsin C | AI246687 | 2.70 |
| **231274_s_at** | MSCP | Solute carrier family 25, member 37 | R92925 | 2.69 |
| **225604_s_at** | C9orf19 | chromosome 9 open reading frame 19 | AA284532 | 2.69 |
| **244235_at** | IVNS1ABP | influenza virus NS1A binding protein | AW273860 | 2.68 |
| **226397_s_at** | --- | Full length insert cDNA clone ZE08A03 | BG502771 | 2.68 |
| **219947_at** | CLEC4A | C-type lectin domain family 4, member A | NM_016184 | 2.67 |
| **203373_at** | SOCS2 | suppressor of cytokine signaling 2 | NM_003877 | 2.67 |
| **237252_at** | THBD | thrombomodulin | AW119113 | 2.67 |
| **226982_at** | ELL2 | elongation factor, RNA polymerase II, 2 | AI745624 | 2.67 |
| **225922_at** | FLJ25371 | Hypothetical protein FLJ25371 | BE501838 | 2.66 |
| **233689_at** | TCF7L2 | Transcription factor 7-like 2 (T-cell specific, HMG-box) | AK026816 | 2.66 |
| **226762_at** | PURB | purine-rich element binding protein B | AV709094 | 2.66 |
| **232587_at** | EML4 | echinoderm microtubule associated protein like 4 | AI208611 | 2.65 |
| **231312_at** | --- | Transcribed locus | W87470 | 2.65 |
| **210233_at** | IL1RAP | interleukin 1 receptor accessory protein | AF167343 | 2.65 |
| **204048_s_at** | PHACTR2 | phosphatase and actin regulator 2 | AA551142 | 2.65 |
| **211984_at** | CALM1 | calmodulin 1 (phosphorylase kinase, delta) | AI653730 | 2.64 |
| **200959_at** | FUS | fusion (involved in t(12;16) in malignant liposarcoma) | NM_004960 | 2.64 |
| **235299_at** | --- | Transcribed locus, weakly similar to XP_510104.1 PREDICTED: similar to hypothetical protein FLJ25224 [Pan troglodytes] | AI769269 | 2.63 |
| **207181_s_at** | CASP7 | caspase 7, apoptosis-related cysteine peptidase | NM_001227 | 2.63 |
| **226034_at** | --- | Homo sapiens, clone IMAGE:3881549, mRNA | BE222344 | 2.63 |
| **214974_x_at** | CXCL5 | chemokine (C-X-C motif) ligand 5 | AK026546 | 2.62 |
| **214511_x_at** | FCGR1A /// LOC440607 | Fc fragment of IgG, high affinity Ia, receptor (CD64) /// Fc-gamma receptor I B2 | L03419 | 2.62 |
| **204014_at** | DUSP4 | dual specificity phosphatase 4 | NM_001394 | 2.62 |
| **202688_at** | TNFSF10 | tumor necrosis factor (ligand) superfamily, member 10 /// tumor necrosis factor (ligand) superfamily, member 10 | NM_003810 | 2.61 |
| **215101_s_at** | CXCL5 | chemokine (C-X-C motif) ligand 5 | BG166705 | 2.61 |
| **235028_at** | --- | CDNA FLJ46440 fis, clone THYMU3016022 | BG288330 | 2.61 |
| **228758_at** | --- | Hypothetical LOC389185 | AW264036 | 2.61 |
| **1569190_at** | FLJ30655 | hypothetical protein FLJ30655 | BC014677 | 2.60 |
| **232331_at** | PPARG | Peroxisome proliferative activated receptor, gamma | AK027107 | 2.60 |
| **219938_s_at** | PSTPIP2 | proline-serine-threonine phosphatase interacting protein 2 | NM_024430 | 2.59 |
| **219191_s_at** | BIN2 | bridging integrator 2 | NM_016293 | 2.59 |
| **217823_s_at** | UBE2J1 | ubiquitin-conjugating enzyme E2, J1 (UBC6 homolog, yeast) | AL562528 | 2.59 |
| **217503_at** | --- | --- | AA203487 | 2.59 |
| **201280_s_at** | DAB2 | disabled homolog 2, mitogen-responsive phosphoprotein (Drosophila) | NM_001343 | 2.59 |
| **211764_s_at** | UBE2D1 | ubiquitin-conjugating enzyme E2D 1 (UBC4/5 homolog, yeast) /// ubiquitin-conjugating enzyme E2D 1 (UBC4/5 homolog, yeast) | BC005980 | 2.57 |
| **212565_at** | STK38L | serine/threonine kinase 38 like | BE302191 | 2.57 |
| **236213_at** | NFE2L2 | Nuclear factor (erythroid-derived 2)-like 2 | AI809760 | 2.57 |
| **202193_at** | LIMK2 | LIM domain kinase 2 | NM_005569 | 2.57 |
| **204951_at** | RHOH | ras homolog gene family, member H | NM_004310 | 2.57 |
| **205633_s_at** | ALAS1 | aminolevulinate, delta-, synthase 1 | NM_000688 | 2.56 |
| **206513_at** | AIM2 | absent in melanoma 2 | NM_004833 | 2.56 |
| **217092_x_at** | RPL7 /// LOC90193 /// LOC388401 /// LOC389305 /// LOC392550 /// LOC439954 | ribosomal protein L7 /// similar to ribosomal protein L7 /// similar to ribosomal protein L7 /// similar to 60S ribosomal protein L7 /// similar to 60S ribosomal protein L7 /// similar to 60S ribosomal protein L7 | AL031589 | 2.56 |
| **202149_at** | NEDD9 | neural precursor cell expressed, developmentally down-regulated 9 | AL136139 | 2.56 |
| **223984_s_at** | NUPL1 | nucleoporin like 1 | BC001104 | 2.56 |
| **225414_at** | RNF149 | ring finger protein 149 | AL558987 | 2.55 |
| **201865_x_at** | NR3C1 | nuclear receptor subfamily 3, group C, member 1 (glucocorticoid receptor) | AI432196 | 2.55 |
| **221561_at** | SOAT1 | sterol O-acyltransferase (acyl-Coenzyme A: cholesterol acyltransferase) 1 | L21934 | 2.55 |
| **236488_s_at** | --- | CDNA FLJ36309 fis, clone THYMU2004986 | AI683805 | 2.55 |
| **212761_at** | TCF7L2 | transcription factor 7-like 2 (T-cell specific, HMG-box) | AI949687 | 2.54 |
| **203234_at** | UPP1 | uridine phosphorylase 1 | NM_003364 | 2.54 |
| **227056_at** | KIAA0141 | KIAA0141 | AA181172 | 2.54 |
| **224392_s_at** | OPN3 | opsin 3 (encephalopsin, panopsin) /// opsin 3 (encephalopsin, panopsin) | AF303588 | 2.54 |
| **211671_s_at** | NR3C1 | nuclear receptor subfamily 3, group C, member 1 (glucocorticoid receptor) /// nuclear receptor subfamily 3, group C, member 1 (glucocorticoid receptor) | U01351 | 2.53 |
| **212511_at** | PICALM | phosphatidylinositol binding clathrin assembly protein | AI766247 | 2.53 |
| **216321_s_at** | NR3C1 | nuclear receptor subfamily 3, group C, member 1 (glucocorticoid receptor) | X03348 | 2.53 |
| **204049_s_at** | PHACTR2 | phosphatase and actin regulator 2 | NM_014721 | 2.53 |
| **204472_at** | GEM | GTP binding protein overexpressed in skeletal muscle | NM_005261 | 2.52 |
| **205992_s_at** | IL15 | interleukin 15 | NM_000585 | 2.51 |
| **227364_at** | --- | --- | AW084125 | 2.51 |
| **202843_at** | DNAJB9 | DnaJ (Hsp40) homolog, subfamily B, member 9 | NM_012328 | 2.50 |
| **202043_s_at** | SMS | spermine synthase | NM_004595 | 2.50 |
| **1554414_a_at** | C8orf1 | chromosome 8 open reading frame 1 | BC031054 | 2.49 |
| **218951_s_at** | PLCXD1 | phosphatidylinositol-specific phospholipase C, X domain containing 1 | NM_018390 | 2.49 |
| **207657_x_at** | TNPO1 | transportin 1 | NM_002270 | 2.49 |
| **231967_at** | PHF20L1 | PHD finger protein 20-like 1 | AI913146 | 2.49 |
| **210757_x_at** | DAB2 | disabled homolog 2, mitogen-responsive phosphoprotein (Drosophila) | AF188298 | 2.48 |
| **225612_s_at** | B3GNT5 | UDP-GlcNAc:betaGal beta-1,3-N-acetylglucosaminyltransferase 5 | BE672260 | 2.48 |
| **205114_s_at** | CCL3 /// CCL3L1 /// CCL3L3 | chemokine (C-C motif) ligand 3 /// chemokine (C-C motif) ligand 3-like 1 /// chemokine (C-C motif) ligand 3-like 3 | NM_002983 | 2.48 |
| **212978_at** | TA-LRRP | Leucine rich repeat containing 8 family, member B | AU146004 | 2.48 |
| **212989_at** | TMEM23 | transmembrane protein 23 | AI377497 | 2.48 |
| **1560396_at** | KLHL6 | Kelch-like 6 (Drosophila) | AK097976 | 2.47 |
| **241342_at** | TMEM65 | transmembrane protein 65 | BG288115 | 2.47 |
| **1552711_a_at** | FLJ32499 | hypothetical protein FLJ32499 | NM_144607 | 2.47 |
| **218451_at** | CDCP1 | CUB domain containing protein 1 | NM_022842 | 2.47 |
| **232392_at** | SFRS3 | Splicing factor, arginine/serine-rich 3 | BE927772 | 2.46 |
| **227786_at** | THRAP6 | thyroid hormone receptor associated protein 6 | AI026938 | 2.46 |
| **202748_at** | GBP2 | guanylate binding protein 2, interferon-inducible /// guanylate binding protein 2, interferon-inducible | NM_004120 | 2.46 |
| **200731_s_at** | PTP4A1 | protein tyrosine phosphatase type IVA, member 1 | AW165960 | 2.46 |
| **204015_s_at** | DUSP4 | dual specificity phosphatase 4 | BC002671 | 2.45 |
| **226109_at** | C21orf91 | chromosome 21 open reading frame 91 | AK023825 | 2.45 |
| **215966_x_at** | GK | glycerol kinase | AA292874 | 2.45 |
| **209545_s_at** | RIPK2 | receptor-interacting serine-threonine kinase 2 | AF064824 | 2.45 |
| **1555858_at** | THUMPD3 | THUMP domain containing 3 | CA430188 | 2.45 |
| **1553096_s_at** | BCL2L11 | BCL2-like 11 (apoptosis facilitator) | NM_138627 | 2.45 |
| **203006_at** | INPP5A | inositol polyphosphate-5-phosphatase, 40kDa | NM_005539 | 2.44 |
| **226345_at** | ARL8 | ADP-ribosylation factor-like 8 | AW270158 | 2.44 |
| **204769_s_at** | TAP2 | transporter 2, ATP-binding cassette, sub-family B (MDR/TAP) | M74447 | 2.44 |
| **221477_s_at** | MGC5618 | hypothetical protein MGC5618 | BF575213 | 2.44 |
| **231644_at** | --- | Transcribed locus | AW016812 | 2.44 |
| **231970_at** | C14orf118 | Chromosome 14 open reading frame 118 | AK025117 | 2.43 |
| **238021_s_at** | LOC388279 | hypothetical gene supported by AF275804 | AA954994 | 2.43 |
| **205819_at** | MARCO | macrophage receptor with collagenous structure /// macrophage receptor with collagenous structure | NM_006770 | 2.43 |
| **227609_at** | EPSTI1 | epithelial stromal interaction 1 (breast) | AA633203 | 2.43 |
| **201866_s_at** | NR3C1 | nuclear receptor subfamily 3, group C, member 1 (glucocorticoid receptor) | NM_000176 | 2.43 |
| **225290_at** | --- | MRNA; cDNA DKFZp566C034 (from clone DKFZp566C034) | AV692425 | 2.42 |
| **222646_s_at** | ERO1L | ERO1-like (S. cerevisiae) | AW268365 | 2.41 |
| **229951_x_at** | --- | LOC440667 | H24473 | 2.41 |
| **224298_s_at** | PHGDHL1 | phosphoglycerate dehydrogenase like 1 | BC004528 | 2.41 |
| **223069_s_at** | EML4 | echinoderm microtubule associated protein like 4 | AF177377 | 2.41 |
| **210258_at** | RGS13 | regulator of G-protein signalling 13 | AF030107 | 2.40 |
| **201662_s_at** | ACSL3 | acyl-CoA synthetase long-chain family member 3 | D89053 | 2.40 |
| **206173_x_at** | GABPB2 | GA binding protein transcription factor, beta subunit 2 | NM_002041 | 2.39 |
| **212506_at** | PICALM | phosphatidylinositol binding clathrin assembly protein | AL135735 | 2.39 |
| **44120_at** | ADCK2 | aarF domain containing kinase 2 | AI879381 | 2.39 |
| **244598_at** | --- | Full length insert cDNA clone ZD66F04 | W72060 | 2.39 |
| **241418_at** | --- | Hypothetical LOC344887 | AI819386 | 2.39 |
| **239143_x_at** | RNF138 | ring finger protein 138 | AW291944 | 2.38 |
| **208810_at** | DNAJB6 | DnaJ (Hsp40) homolog, subfamily B, member 6 | AF080569 | 2.38 |
| **232068_s_at** | TLR4 | toll-like receptor 4 | AF177765 | 2.38 |
| **1561181_at** | ARID5B | AT rich interactive domain 5B (MRF1-like) | AI075770 | 2.38 |
| **210145_at** | PLA2G4A | phospholipase A2, group IVA (cytosolic, calcium-dependent) | M68874 | 2.38 |
| **228899_at** | LOC441297 | LOC441297 | AI870903 | 2.38 |
| **225415_at** | DTX3L | deltex 3-like (Drosophila) | AA577672 | 2.38 |
| **218450_at** | HEBP1 | heme binding protein 1 | NM_015987 | 2.37 |
| **221062_at** | HS3ST3B1 | heparan sulfate (glucosamine) 3-O-sulfotransferase 3B1 | NM_006041 | 2.37 |
| **202241_at** | TRIB1 | tribbles homolog 1 (Drosophila) | NM_025195 | 2.37 |
| **212502_at** | C10orf22 | chromosome 10 open reading frame 22 | AV713053 | 2.37 |
| **222808_at** | GLT28D1 | glycosyltransferase 28 domain containing 1 | BC005336 | 2.36 |
| **223798_at** | SLC41A2 | solute carrier family 41, member 2 | AL136828 | 2.36 |
| **203651_at** | ZFYVE16 | zinc finger, FYVE domain containing 16 | NM_014733 | 2.36 |
| **204747_at** | IFIT3 | interferon-induced protein with tetratricopeptide repeats 3 | NM_001549 | 2.36 |
| **226392_at** | --- | CDNA: FLJ21652 fis, clone COL08582 | AI888503 | 2.36 |
| **214146_s_at** | PPBP | pro-platelet basic protein (chemokine (C-X-C motif) ligand 7) | R64130 | 2.35 |
| **226460_at** | KIAA1450 | KIAA1450 protein | AB040883 | 2.35 |
| **211527_x_at** | VEGF | vascular endothelial growth factor | M27281 | 2.35 |
| **216950_s_at** | FCGR1A | Fc fragment of IgG, high affinity Ia, receptor (CD64) | X14355 | 2.35 |
| **225924_at** | FLJ25371 | Hypothetical protein FLJ25371 | AI478634 | 2.34 |
| **207907_at** | TNFSF14 | tumor necrosis factor (ligand) superfamily, member 14 | NM_003807 | 2.34 |
| **244251_at** | LCP2 | Lymphocyte cytosolic protein 2 (SH2 domain containing leukocyte protein of 76kDa) | BF363430 | 2.34 |
| **225406_at** | TWSG1 | twisted gastrulation homolog 1 (Drosophila) | AA195009 | 2.33 |
| **1553736_at** | MGC23401 | hypothetical protein MGC23401 | NM_144982 | 2.31 |
| **1555780_a_at** | RHEB | Ras homolog enriched in brain | AF493921 | 2.31 |
| **202901_x_at** | CTSS | cathepsin S | BC002642 | 2.31 |
| **1554285_at** | HAVCR2 | hepatitis A virus cellular receptor 2 | AF450243 | 2.30 |
| **231412_at** | DKFZp686L14188 | hypothetical gene supported by BX538329 | H04388 | 2.30 |
| **225878_at** | KIF1B | Kinesin family member 1B | AL135264 | 2.30 |
| **213069_at** | HEG | HEG homolog 1 (zebrafish) | AI148659 | 2.29 |
| **227571_at** | --- | CDNA FLJ35556 fis, clone SPLEN2004844 | AW516665 | 2.29 |
| **1561042_at** | EMR3 | Egf-like module containing, mucin-like, hormone receptor-like 3 | AF086249 | 2.29 |
| **226333_at** | IL6R | interleukin 6 receptor | AV700030 | 2.29 |
| **212619_at** | KIAA0286 | KIAA0286 protein | AW205215 | 2.29 |
| **218856_at** | TNFRSF21 | tumor necrosis factor receptor superfamily, member 21 | NM_016629 | 2.28 |
| **228674_s_at** | EML4 | echinoderm microtubule associated protein like 4 | AA524507 | 2.28 |
| **218197_s_at** | OXR1 | oxidation resistance 1 | NM_018002 | 2.28 |
| **213440_at** | RAB1A | RAB1A, member RAS oncogene family | AL530264 | 2.28 |
| **1554999_at** | RASGEF1B | RasGEF domain family, member 1B | BC036784 | 2.28 |
| **204285_s_at** | PMAIP1 | phorbol-12-myristate-13-acetate-induced protein 1 | AI857639 | 2.28 |
| **226335_at** | RPS6KA3 | ribosomal protein S6 kinase, 90kDa, polypeptide 3 | BG498334 | 2.28 |
| **241956_at** | PCGF5 | Polycomb group ring finger 5 | AI521883 | 2.28 |
| **209310_s_at** | CASP4 | caspase 4, apoptosis-related cysteine peptidase | U25804 | 2.28 |
| **202679_at** | NPC1 | Niemann-Pick disease, type C1 | NM_000271 | 2.27 |
| **236439_at** | BCL6 | B-cell CLL/lymphoma 6 (zinc finger protein 51) | AI733564 | 2.27 |
| **209967_s_at** | CREM | cAMP responsive element modulator | D14826 | 2.27 |
| **212274_at** | LPIN1 | lipin 1 | AV705559 | 2.26 |
| **219492_at** | CHIC2 | cysteine-rich hydrophobic domain 2 | NM_012110 | 2.26 |
| **218195_at** | C6orf211 | chromosome 6 open reading frame 211 | NM_024573 | 2.26 |
| **223288_at** | USP38 | ubiquitin specific peptidase 38 | AW977401 | 2.25 |
| **222412_s_at** | SSR3 | signal sequence receptor, gamma (translocon-associated protein gamma) | AW150923 | 2.25 |
| **203745_at** | HCCS | holocytochrome c synthase (cytochrome c heme-lyase) | AI801013 | 2.25 |
| **232052_at** | THUMPD3 | THUMP domain containing 3 | AL110136 | 2.25 |
| **226914_at** | ARPC5L | actin related protein 2/3 complex, subunit 5-like | AU158936 | 2.25 |
| **211367_s_at** | CASP1 | caspase 1, apoptosis-related cysteine peptidase (interleukin 1, beta, convertase) | U13699 | 2.24 |
| **200629_at** | WARS | tryptophanyl-tRNA synthetase | NM_004184 | 2.24 |
| **228029_at** | KIAA1982 | KIAA1982 protein | AW513477 | 2.24 |
| **202284_s_at** | CDKN1A | cyclin-dependent kinase inhibitor 1A (p21, Cip1) | NM_000389 | 2.23 |
| **228042_at** | ADPRH | ADP-ribosylarginine hydrolase | N54957 | 2.22 |
| **231897_at** | LTB4DH | leukotriene B4 12-hydroxydehydrogenase | AL135787 | 2.22 |
| **235643_at** | SAMD9L | sterile alpha motif domain containing 9-like | BE886225 | 2.22 |
| **209451_at** | TANK | TRAF family member-associated NFKB activator | U59863 | 2.22 |
| **241837_at** | ARID5B | AT rich interactive domain 5B (MRF1-like) | AI289774 | 2.22 |
| **227620_at** | --- | --- | AV721564 | 2.22 |
| **202693_s_at** | STK17A | serine/threonine kinase 17a (apoptosis-inducing) | AW194730 | 2.22 |
| **207791_s_at** | RAB1A | RAB1A, member RAS oncogene family | NM_004161 | 2.21 |
| **225749_at** | LOC283951 | hypothetical protein LOC283951 | BE889319 | 2.21 |
| **210942_s_at** | ST3GAL6 | ST3 beta-galactoside alpha-2,3-sialyltransferase 6 | AB022918 | 2.21 |
| **226038_at** | LONRF1 | LON peptidase N-terminal domain and ring finger 1 | BF680438 | 2.21 |
| **226525_at** | STK17B | Serine/threonine kinase 17b (apoptosis-inducing) | N51102 | 2.21 |
| **210017_at** | MALT1 | mucosa associated lymphoid tissue lymphoma translocation gene 1 | AF070528 | 2.20 |
| **211286_x_at** | CSF2RA | colony stimulating factor 2 receptor, alpha, low-affinity (granulocyte-macrophage) | L29349 | 2.20 |
| **204194_at** | BACH1 | BTB and CNC homology 1, basic leucine zipper transcription factor 1 | NM_001186 | 2.19 |
| **226505_x_at** | USP32 | ubiquitin specific peptidase 32 | AI148567 | 2.18 |
| **211317_s_at** | CFLAR | CASP8 and FADD-like apoptosis regulator | AF041461 | 2.18 |
| **229450_at** | IFIT3 | interferon-induced protein with tetratricopeptide repeats 3 | AI075407 | 2.18 |
| **204024_at** | C8orf1 | chromosome 8 open reading frame 1 | NM_004337 | 2.18 |
| **218085_at** | CHMP5 | chromatin modifying protein 5 | NM_015961 | 2.18 |
| **227038_at** | MGC26963 | hypothetical protein MGC26963 | AI963083 | 2.17 |
| **215977_x_at** | GK | glycerol kinase | X68285 | 2.17 |
| **203897_at** | LOC57149 | hypothetical protein A-211C6.1 | BE963444 | 2.17 |
| **230621_at** | LOC285148 | hypothetical protein LOC285148 | AA502936 | 2.16 |
| **235592_at** | ELL2 | Elongation factor, RNA polymerase II, 2 | AW960145 | 2.16 |
| **206522_at** | MGAM | maltase-glucoamylase (alpha-glucosidase) | NM_004668 | 2.15 |
| **231093_at** | FCRH3 | Fc receptor-like 3 | BF514552 | 2.15 |
| **1554472_a_at** | PHF20L1 | PHD finger protein 20-like 1 | BC015211 | 2.15 |
| **212289_at** | ANKRD12 | ankyrin repeat domain 12 | AB020681 | 2.14 |
| **205220_at** | GPR109B | G protein-coupled receptor 109B /// G protein-coupled receptor 109B | NM_006018 | 2.14 |
| **201266_at** | TXNRD1 | thioredoxin reductase 1 | NM_003330 | 2.13 |
| **224806_at** | TRIM25 | tripartite motif-containing 25 | BE563152 | 2.12 |
| **203742_s_at** | TDG | thymine-DNA glycosylase | BF674842 | 2.12 |
| **209571_at** | CIR | CBF1 interacting corepressor | U03644 | 2.12 |
| **226686_at** | LOC493856 | similar to RIKEN cDNA 1500009M05 gene | AI188518 | 2.11 |
| **217299_s_at** | NBN | nibrin | AK001017 | 2.11 |
| **203888_at** | THBD | thrombomodulin | NM_000361 | 2.10 |
| **214430_at** | GLA | galactosidase, alpha | NM_000169 | 2.08 |
| **224685_at** | MLLT4 | myeloid/lymphoid or mixed-lineage leukemia (trithorax homolog, Drosophila); translocated to, 4 | AI675354 | 2.07 |
| **208485_x_at** | CFLAR | CASP8 and FADD-like apoptosis regulator | NM_003879 | 2.06 |
| **239843_at** | RIT1 | Ras-like without CAAX 1 | AI655057 | 2.06 |
| **220123_at** | SLC35F5 | solute carrier family 35, member F5 | NM_025181 | 2.05 |
| **201088_at** | KPNA2 | karyopherin alpha 2 (RAG cohort 1, importin alpha 1) | NM_002266 | 2.04 |
| **218404_at** | SNX10 | sorting nexin 10 | NM_013322 | 2.03 |
| **217915_s_at** | C15orf15 | chromosome 15 open reading frame 15 | NM_016304 | 2.03 |
| **203510_at** | MET | met proto-oncogene (hepatocyte growth factor receptor) | BG170541 | 2.03 |
| **201536_at** | DUSP3 | dual specificity phosphatase 3 (vaccinia virus phosphatase VH1-related) | AL048503 | -2.00 |
| **226554_at** | ZBTB7A | zinc finger and BTB domain containing 7A | AW445134 | -2.01 |
| **202412_s_at** | USP1 | ubiquitin specific peptidase 1 | AW499935 | -2.02 |
| **202840_at** | TAF15 | TAF15 RNA polymerase II, TATA box binding protein (TBP)-associated factor, 68kDa | NM_003487 | -2.02 |
| **203153_at** | IFIT1 | interferon-induced protein with tetratricopeptide repeats 1 /// interferon-induced protein with tetratricopeptide repeats 1 | NM_001548 | -2.03 |
| **244811_at** | IRAK1BP1 | Interleukin-1 receptor-associated kinase 1 binding protein 1 | AI561173 | -2.04 |
| **228244_at** | BLOC1S3 | Biogenesis of lysosome-related organelles complex-1, subunit 3 | BF062383 | -2.04 |
| **AFFX-M27830_5_at** | SOX18 | SRY (sex determining region Y)-box 18 | AFFX-M27830_5 | -2.05 |
| **204638_at** | ACP5 | acid phosphatase 5, tartrate resistant | NM_001611 | -2.05 |
| **204425_at** | ARHGAP4 | Rho GTPase activating protein 4 | NM_001666 | -2.05 |
| **1559502_s_at** | LRRC25 | leucine rich repeat containing 25 | AJ422148 | -2.05 |
| **227122_at** | --- | Transcribed locus, moderately similar to XP_512541.1 PREDICTED: similar to hypothetical protein [Pan troglodytes] | AI807498 | -2.06 |
| **222413_s_at** | MLL3 | myeloid/lymphoid or mixed-lineage leukemia 3 | AW137099 | -2.06 |
| **222468_at** | KIAA0319L | KIAA0319-like | W58365 | -2.06 |
| **212080_at** | LOC143941 | Similar to CDNA sequence BC021608 | AV714029 | -2.06 |
| **202365_at** | MGC5139 | hypothetical protein MGC5139 | BC004815 | -2.07 |
| **1566509_s_at** | FBXO9 | F-box protein 9 | AK095315 | -2.07 |
| **209307_at** | SWAP70 | SWAP-70 protein | AB014540 | -2.07 |
| **234302_s_at** | FLJ20308 | hypothetical protein FLJ20308 | AL137263 | -2.07 |
| **240249_at** | --- | Transcribed locus | R40316 | -2.07 |
| **206855_s_at** | HYAL2 | hyaluronoglucosaminidase 2 | NM_003773 | -2.07 |
| **223591_at** | RNF135 | ring finger protein 135 | BC005084 | -2.08 |
| **201388_at** | PSMD3 | proteasome (prosome, macropain) 26S subunit, non-ATPase, 3 | NM_002809 | -2.09 |
| **1559582_at** | RHOQ | ras homolog gene family, member Q | BC033251 | -2.09 |
| **218530_at** | FHOD1 | formin homology 2 domain containing 1 | NM_013241 | -2.10 |
| **203162_s_at** | KATNB1 | katanin p80 (WD repeat containing) subunit B 1 | NM_005886 | -2.10 |
| **1559759_at** | KIFC3 | Kinesin family member C3 | BE407830 | -2.10 |
| **209517_s_at** | ASH2L | ash2 (absent, small, or homeotic)-like (Drosophila) | AB020982 | -2.10 |
| **204276_at** | TK2 | thymidine kinase 2, mitochondrial | BE895437 | -2.10 |
| **230630_at** | AK3 | Adenylate kinase 3-like 1 | AI566130 | -2.10 |
| **45288_at** | ABHD6 | abhydrolase domain containing 6 | AA209239 | -2.10 |
| **216071_x_at** | MED12 | mediator of RNA polymerase II transcription, subunit 12 homolog (yeast) | AF132033 | -2.11 |
| **215031_x_at** | RNF126 | ring finger protein 126 | BG420893 | -2.11 |
| **210283_x_at** | PAIP1 /// LOC388345 | poly(A) binding protein interacting protein 1 /// similar to poly(A) binding protein interacting protein 1 isoform 1; polyadenylate binding protein-interacting protein 1; PABC1-interacting protein 1 | BC005295 | -2.11 |
| **212024_x_at** | FLII | flightless I homolog (Drosophila) | U80184 | -2.11 |
| **210266_s_at** | TRIM33 | tripartite motif-containing 33 | AF220137 | -2.12 |
| **208982_at** | PECAM1 | Platelet/endothelial cell adhesion molecule (CD31 antigen) | AW574504 | -2.12 |
| **227847_at** | EPM2AIP1 | EPM2A (laforin) interacting protein 1 | BF432224 | -2.12 |
| **202065_s_at** | PPFIA1 | protein tyrosine phosphatase, receptor type, f polypeptide (PTPRF), interacting protein (liprin), alpha 1 | BG033593 | -2.12 |
| **202962_at** | KIF13B | kinesin family member 13B | NM_015254 | -2.13 |
| **1560297_at** | HM13 | Histocompatibility (minor) 13 | BG928538 | -2.13 |
| **210044_s_at** | LYL1 | lymphoblastic leukemia derived sequence 1 | BC002796 | -2.13 |
| **203096_s_at** | RAPGEF2 | Rap guanine nucleotide exchange factor (GEF) 2 | BF439282 | -2.13 |
| **226298_at** | RUNDC1 | RUN domain containing 1 | BE394201 | -2.13 |
| **240240_at** | UBE2J2 | Ubiquitin-conjugating enzyme E2, J2 (UBC6 homolog, yeast) | R10087 | -2.13 |
| **226975_at** | RNPC3 | RNA-binding region (RNP1, RRM) containing 3 | BF116157 | -2.13 |
| **232095_at** | SRGAP2 | SLIT-ROBO Rho GTPase activating protein 2 | BG109134 | -2.13 |
| **209932_s_at** | DUT | dUTP pyrophosphatase | U90223 | -2.14 |
| **211976_at** | RPL35 | Ribosomal protein L35 | AK026168 | -2.14 |
| **204952_at** | LYPD3 | LY6/PLAUR domain containing 3 | NM_014400 | -2.14 |
| **212452_x_at** | MYST4 | MYST histone acetyltransferase (monocytic leukemia) 4 | AF113514 | -2.14 |
| **222619_at** | ZNF281 | Zinc finger protein 281 | AU150752 | -2.15 |
| **203385_at** | DGKA | diacylglycerol kinase, alpha 80kDa | NM_001345 | -2.15 |
| **201783_s_at** | RELA | v-rel reticuloendotheliosis viral oncogene homolog A, nuclear factor of kappa light polypeptide gene enhancer in B-cells 3, p65 (avian) | NM_021975 | -2.15 |
| **236794_at** | CTBP2 | C-terminal binding protein 2 | AW629436 | -2.16 |
| **203278_s_at** | PHF21A | PHD finger protein 21A | NM_016621 | -2.16 |
| **1554287_at** | TRIM4 | tripartite motif-containing 4 | BC011763 | -2.16 |
| **216705_s_at** | ADA | adenosine deaminase | X02189 | -2.16 |
| **219563_at** | C14orf139 | chromosome 14 open reading frame 139 | NM_024633 | -2.16 |
| **202944_at** | NAGA | N-acetylgalactosaminidase, alpha- | NM_000262 | -2.16 |
| **201561_s_at** | CLSTN1 | calsyntenin 1 | NM_014944 | -2.16 |
| **201730_s_at** | TPR | translocated promoter region (to activated MET oncogene) | BF110993 | -2.17 |
| **210347_s_at** | BCL11A | B-cell CLL/lymphoma 11A (zinc finger protein) | AF080216 | -2.18 |
| **218661_at** | FLJ14154 | hypothetical protein FLJ14154 | NM_024845 | -2.18 |
| **212211_at** | ANKRD17 | ankyrin repeat domain 17 | AI986295 | -2.18 |
| **225995_x_at** | MGC52000 | CXYorf1-related protein | BG178695 | -2.18 |
| **210146_x_at** | LILRB2 | leukocyte immunoglobulin-like receptor, subfamily B (with TM and ITIM domains), member 2 | AF004231 | -2.18 |
| **226054_at** | BRD4 | bromodomain containing 4 | AA702437 | -2.19 |
| **227999_at** | PWWP2 | PWWP domain containing 2 | AI290476 | -2.19 |
| **203879_at** | PIK3CD | phosphoinositide-3-kinase, catalytic, delta polypeptide /// phosphoinositide-3-kinase, catalytic, delta polypeptide | U86453 | -2.19 |
| **221767_x_at** | HDLBP | high density lipoprotein binding protein (vigilin) | AA515560 | -2.19 |
| **211962_s_at** | ZFP36L1 | zinc finger protein 36, C3H type-like 1 | BG250310 | -2.19 |
| **223008_s_at** | C9orf5 | chromosome 9 open reading frame 5 | AF153415 | -2.19 |
| **208420_x_at** | SUPT6H | suppressor of Ty 6 homolog (S. cerevisiae) | NM_003170 | -2.20 |
| **200758_s_at** | NFE2L1 | nuclear factor (erythroid-derived 2)-like 1 | AI361227 | -2.20 |
| **AFFX-HUMRGE/M10098_5_at** | --- | --- | AFFX-HUMRGE/M10098_5 | -2.21 |
| **209568_s_at** | RGL1 | ral guanine nucleotide dissociation stimulator-like 1 | AF186779 | -2.21 |
| **239234_at** | OGDH | Oxoglutarate (alpha-ketoglutarate) dehydrogenase (lipoamide) | AW452419 | -2.21 |
| **229322_at** | PPP2R5E | protein phosphatase 2, regulatory subunit B (B56), epsilon isoform | BF529715 | -2.21 |
| **208070_s_at** | REV3L | REV3-like, catalytic subunit of DNA polymerase zeta (yeast) | NM_002912 | -2.21 |
| **218301_at** | RNPEPL1 | arginyl aminopeptidase (aminopeptidase B)-like 1 | NM_018226 | -2.22 |
| **209257_s_at** | CSPG6 | chondroitin sulfate proteoglycan 6 (bamacan) | BF795297 | -2.22 |
| **221765_at** | UGCG | UDP-glucose ceramide glucosyltransferase | AI378044 | -2.22 |
| **222876_s_at** | CENTA2 | centaurin, alpha 2 | AI761520 | -2.23 |
| **243720_at** | CMIP | C-Maf-inducing protein | AA039576 | -2.23 |
| **224716_at** | SLC35B2 | solute carrier family 35, member B2 | BG163267 | -2.23 |
| **231406_at** | LOC401394 /// LOC402578 | hypothetical LOC401394 /// hypothetical LOC402578 | AW205664 | -2.23 |
| **212152_x_at** | ARID1A | AT rich interactive domain 1A (SWI- like) | AI679080 | -2.24 |
| **212886_at** | DKFZP434C171 | DKFZP434C171 protein | AL080169 | -2.24 |
| **214560_at** | FPRL2 | formyl peptide receptor-like 2 | NM_002030 | -2.24 |
| **211417_x_at** | GGT1 | gamma-glutamyltransferase 1 | L20493 | -2.24 |
| **224735_at** | CYBASC3 | cytochrome b, ascorbate dependent 3 | AA683481 | -2.24 |
| **212146_at** | PLEKHM2 | pleckstrin homology domain containing, family M (with RUN domain) member 2 | AB020649 | -2.24 |
| **201913_s_at** | COASY | Coenzyme A synthase | NM_025233 | -2.25 |
| **202117_at** | ARHGAP1 | Rho GTPase activating protein 1 | BG468434 | -2.25 |
| **213039_at** | ARHGEF18 | rho/rac guanine nucleotide exchange factor (GEF) 18 | AB011093 | -2.25 |
| **224572_s_at** | IRF2BP2 | interferon regulatory factor 2 binding protein 2 | BG485163 | -2.26 |
| **1552621_at** | POLR2J2 | DNA directed RNA polymerase II polypeptide J-related gene | BQ613856 | -2.26 |
| **217827_s_at** | SPG21 | spastic paraplegia 21 (autosomal recessive, Mast syndrome) | NM_016630 | -2.26 |
| **1556067_a_at** | JMJD3 | jumonji domain containing 3 | AI830331 | -2.26 |
| **212547_at** | FLJ35348 /// BRD3 | FLJ35348 /// Bromodomain containing 3 | N34842 | -2.26 |
| **200760_s_at** | ARL6IP5 | ADP-ribosylation-like factor 6 interacting protein 5 | N92494 | -2.26 |
| **226102_at** | ZNF192 | Zinc finger protein 192 | AI920953 | -2.26 |
| **74694_s_at** | RABEP2 | rabaptin, RAB GTPase binding effector protein 2 | AA907940 | -2.26 |
| **205546_s_at** | TYK2 | tyrosine kinase 2 | NM_003331 | -2.27 |
| **203556_at** | ZHX2 | zinc fingers and homeoboxes 2 | NM_014943 | -2.27 |
| **227811_at** | FGD3 | FYVE, RhoGEF and PH domain containing 3 | AK000004 | -2.27 |
| **232008_s_at** | BBX | bobby sox homolog (Drosophila) | AF283775 | -2.28 |
| **203973_s_at** | CEBPD | CCAAT/enhancer binding protein (C/EBP), delta | NM_005195 | -2.28 |
| **218061_at** | MEA1 | male-enhanced antigen 1 | NM_014623 | -2.28 |
| **213170_at** | GPX7 | glutathione peroxidase 7 | AA406605 | -2.28 |
| **1557170_at** | NEK8 | NIMA (never in mitosis gene a)- related kinase 8 | AI073943 | -2.28 |
| **37462_i_at** | SF3A2 | splicing factor 3a, subunit 2, 66kDa | L21990 | -2.28 |
| **201168_x_at** | ARHGDIA | Rho GDP dissociation inhibitor (GDI) alpha | NM_004309 | -2.28 |
| **208912_s_at** | CNP | 2',3'-cyclic nucleotide 3' phosphodiesterase | BC001362 | -2.28 |
| **1555495_a_at** | SDCCAG10 | serologically defined colon cancer antigen 10 | BC012117 | -2.28 |
| **225832_s_at** | LOC221955 | KCCR13L | BE795104 | -2.28 |
| **218151_x_at** | GPR172A | G protein-coupled receptor 172A | NM_024531 | -2.28 |
| **233013_x_at** | RPL7L1 /// LOC401197 | ribosomal protein L7-like 1 /// similar to RPL7L1 protein | AU146717 | -2.28 |
| **228008_at** | --- | CDNA clone IMAGE:3462401 | AI972511 | -2.28 |
| **1554516_at** | --- | LOC441434 | BC011779 | -2.29 |
| **228170_at** | OLIG1 | oligodendrocyte transcription factor 1 | AL355743 | -2.29 |
| **205627_at** | CDA | cytidine deaminase | NM_001785 | -2.29 |
| **237768_x_at** | --- | --- | AA825925 | -2.29 |
| **218855_at** | GPR175 | G protein-coupled receptor 175 | NM_016372 | -2.29 |
| **221506_s_at** | TNPO2 | transportin 2 (importin 3, karyopherin beta 2b) | BG258639 | -2.29 |
| **206845_s_at** | RNF40 | ring finger protein 40 | NM_014771 | -2.30 |
| **200852_x_at** | GNB2 | guanine nucleotide binding protein (G protein), beta polypeptide 2 | NM_005273 | -2.30 |
| **219269_at** | FLJ21616 | hypothetical protein FLJ21616 | NM_024567 | -2.30 |
| **225224_at** | C20orf112 | chromosome 20 open reading frame 112 | AL034550 | -2.30 |
| **209346_s_at** | PI4KII | phosphatidylinositol 4-kinase type II | BC003167 | -2.30 |
| **209342_s_at** | IKBKB | inhibitor of kappa light polypeptide gene enhancer in B-cells, kinase beta | AF080158 | -2.30 |
| **201051_at** | ANP32A | Acidic (leucine-rich) nuclear phosphoprotein 32 family, member A | BE560202 | -2.31 |
| **202519_at** | MONDOA | MondoA | NM_014938 | -2.31 |
| **244408_at** | RAPGEF1 | Rap guanine nucleotide exchange factor (GEF) 1 | AA927995 | -2.31 |
| **205740_s_at** | MGC10433 | hypothetical protein MGC10433 | NM_024321 | -2.31 |
| **241346_at** | ARHGAP30 | Rho GTPase activating protein 30 | AW974499 | -2.31 |
| **203286_at** | RNF44 | ring finger protein 44 | NM_014901 | -2.31 |
| **221552_at** | ABHD6 | abhydrolase domain containing 6 | BC001698 | -2.31 |
| **221734_at** | LOC133619 | hypothetical protein MGC12103 | BE328312 | -2.31 |
| **1555446_s_at** | TMEM1 | transmembrane protein 1 | BC046241 | -2.31 |
| **209381_x_at** | SF3A2 | splicing factor 3a, subunit 2, 66kDa | BC004434 | -2.31 |
| **206782_s_at** | DNAJC4 | DnaJ (Hsp40) homolog, subfamily C, member 4 | NM_005528 | -2.31 |
| **219259_at** | SEMA4A | sema domain, immunoglobulin domain (Ig), transmembrane domain (TM) and short cytoplasmic domain, (semaphorin) 4A | NM_022367 | -2.31 |
| **208998_at** | UCP2 | uncoupling protein 2 (mitochondrial, proton carrier) | U94592 | -2.32 |
| **232180_at** | --- | --- | U00954 | -2.32 |
| **212901_s_at** | CSTF2T | cleavage stimulation factor, 3' pre-RNA, subunit 2, 64kDa, tau variant | BF732638 | -2.32 |
| **238077_at** | KCTD6 | potassium channel tetramerisation domain containing 6 | T75480 | -2.32 |
| **221882_s_at** | TMEM8 | transmembrane protein 8 (five membrane-spanning domains) | AI636233 | -2.32 |
| **232396_at** | DKFZp547K1113 | Hypothetical protein DKFZp547K1113 | AV711227 | -2.32 |
| **202212_at** | PES1 | pescadillo homolog 1, containing BRCT domain (zebrafish) | NM_014303 | -2.32 |
| **210208_x_at** | BAT3 | HLA-B associated transcript 3 | BC003133 | -2.32 |
| **226605_at** | DGKQ | diacylglycerol kinase, theta 110kDa | N45308 | -2.33 |
| **206729_at** | TNFRSF8 | tumor necrosis factor receptor superfamily, member 8 | NM_001243 | -2.33 |
| **221800_s_at** | FLJ22175 | hypothetical protein FLJ22175 | AA551370 | -2.33 |
| **60471_at** | RIN3 | Ras and Rab interactor 3 | AA625133 | -2.33 |
| **225731_at** | KIAA1223 | KIAA1223 protein | BF196876 | -2.33 |
| **213649_at** | SFRS7 | splicing factor, arginine/serine-rich 7, 35kDa | AA524053 | -2.33 |
| **218052_s_at** | ATP13A1 | ATPase type 13A1 | NM_020410 | -2.33 |
| **201017_at** | EIF1AX | eukaryotic translation initiation factor 1A, X-linked | BG149698 | -2.33 |
| **229010_at** | CBL | Cas-Br-M (murine) ecotropic retroviral transforming sequence | AI807026 | -2.34 |
| **212269_s_at** | MCM3AP | MCM3 minichromosome maintenance deficient 3 (S. cerevisiae) associated protein | AJ010089 | -2.34 |
| **38487_at** | STAB1 | stabilin 1 | D87433 | -2.34 |
| **44822_s_at** | KIAA1193 | KIAA1193 | AW003889 | -2.35 |
| **206593_s_at** | SURF5 | surfeit 5 | NM_006752 | -2.35 |
| **213140_s_at** | SS18L1 | synovial sarcoma translocation gene on chromosome 18-like 1 | AB014593 | -2.35 |
| **201461_s_at** | MAPKAPK2 | mitogen-activated protein kinase-activated protein kinase 2 | NM_004759 | -2.35 |
| **201732_s_at** | CLCN3 | chloride channel 3 | AF029346 | -2.35 |
| **209275_s_at** | CLN3 | ceroid-lipofuscinosis, neuronal 3, juvenile (Batten, Spielmeyer-Vogt disease) | AF015593 | -2.35 |
| **212420_at** | ELF1 | E74-like factor 1 (ets domain transcription factor) | AL559590 | -2.36 |
| **203777_s_at** | RPS6KB2 | ribosomal protein S6 kinase, 70kDa, polypeptide 2 | NM_003952 | -2.36 |
| **214911_s_at** | BRD2 | bromodomain containing 2 | S78771 | -2.36 |
| **224082_at** | --- | --- | AF113013 | -2.37 |
| **212994_at** | THOC2 | THO complex 2 | BE543527 | -2.37 |
| **225913_at** | KIAA2002 | KIAA2002 protein | AK025943 | -2.37 |
| **215089_s_at** | RBM10 | RNA binding motif protein 10 | AW409974 | -2.38 |
| **212069_s_at** | KIAA0515 | KIAA0515 | AK026025 | -2.38 |
| **226530_at** | BMF | Bcl2 modifying factor | AK024472 | -2.38 |
| **56821_at** | FLJ10815 | amino acid transporter | AI963454 | -2.38 |
| **239393_at** | PAN3 | PABP1-dependent poly A-specific ribonuclease subunit PAN3 | AW510927 | -2.38 |
| **212520_s_at** | SMARCA4 | SWI/SNF related, matrix associated, actin dependent regulator of chromatin, subfamily a, member 4 | AI684141 | -2.38 |
| **230337_at** | SOS1 | son of sevenless homolog 1 (Drosophila) | AW241962 | -2.38 |
| **209229_s_at** | SAPS1 | SAPS domain family, member 1 | BC002799 | -2.39 |
| **225204_at** | TA-PP2C | T-cell activation protein phosphatase 2C | AA521311 | -2.39 |
| **212075_s_at** | CSNK2A1 | casein kinase 2, alpha 1 polypeptide | AI161318 | -2.39 |
| **224714_at** | MKI67IP | MKI67 (FHA domain) interacting nucleolar phosphoprotein | AL542544 | -2.39 |
| **242875_at** | PSEN1 | Presenilin 1 (Alzheimer disease 3) | AI659439 | -2.39 |
| **226005_at** | UBE2G1 | Ubiquitin-conjugating enzyme E2G 1 (UBC7 homolog, yeast) | BG170762 | -2.39 |
| **211074_at** | FOLR1 | folate receptor 1 (adult) /// folate receptor 1 (adult) | AF000381 | -2.40 |
| **204135_at** | DOC1 | downregulated in ovarian cancer 1 | NM_014890 | -2.40 |
| **235009_at** | FAM44A | family with sequence similarity 44, member A | AI049791 | -2.40 |
| **208629_s_at** | HADHA | hydroxyacyl-Coenzyme A dehydrogenase/3-ketoacyl-Coenzyme A thiolase/enoyl-Coenzyme A hydratase (trifunctional protein), alpha subunit | BG472176 | -2.40 |
| **236533_at** | DDEF1 | development and differentiation enhancing factor 1 | AW236958 | -2.41 |
| **38069_at** | CLCN7 | chloride channel 7 | Z67743 | -2.41 |
| **229336_at** | ST3GAL2 | ST3 beta-galactoside alpha-2,3-sialyltransferase 2 | BE300666 | -2.41 |
| **AFFX-hum_alu_at** | ADAR /// ADRBK2 /// etc. | adenosine deaminase, RNA-specific /// adrenergic, beta, receptor kinase 2 /// etc. | AFFX-hum_alu | -2.41 |
| **205312_at** | SPI1 | spleen focus forming virus (SFFV) proviral integration oncogene spi1 | NM_003120 | -2.41 |
| **222062_at** | IL27RA | interleukin 27 receptor, alpha | AI983115 | -2.41 |
| **221496_s_at** | TOB2 | transducer of ERBB2, 2 | D64109 | -2.41 |
| **200616_s_at** | KIAA0152 | KIAA0152 | BC000371 | -2.41 |
| **218714_at** | MGC3121 | hypothetical protein MGC3121 | NM_024031 | -2.42 |
| **1553551_s_at** | --- | --- | NM_173709 | -2.42 |
| **202206_at** | ARL7 | ADP-ribosylation factor-like 7 | AW450363 | -2.42 |
| **223382_s_at** | ZNRF1 | zinc and ring finger 1 | AL136903 | -2.42 |
| **32209_at** | FAM89B | family with sequence similarity 89, member B | AF052151 | -2.42 |
| **242398_x_at** | ATP5F1 | ATP synthase, H+ transporting, mitochondrial F0 complex, subunit b, isoform 1 | AA605121 | -2.43 |
| **241938_at** | QKI | Quaking homolog, KH domain RNA binding (mouse) | AA935633 | -2.43 |
| **210378_s_at** | SSNA1 | Sjogren's syndrome nuclear autoantigen 1 | BC004118 | -2.43 |
| **223166_x_at** | C9orf86 | chromosome 9 open reading frame 86 | BC002945 | -2.43 |
| **227232_at** | EVL | Enah/Vasp-like | T58044 | -2.44 |
| **222313_at** | CNOT2 | CCR4-NOT transcription complex, subunit 2 | AW972359 | -2.44 |
| **219040_at** | CORO7 | coronin 7 | NM_024535 | -2.44 |
| **211937_at** | EIF4B | eukaryotic translation initiation factor 4B | NM_001417 | -2.44 |
| **225373_at** | C10orf54 | chromosome 10 open reading frame 54 | BE271644 | -2.45 |
| **201299_s_at** | MOBK1B | MOB1, Mps One Binder kinase activator-like 1B (yeast) | NM_018221 | -2.45 |
| **227207_x_at** | ZNF213 | zinc finger protein 213 | BF510572 | -2.45 |
| **201182_s_at** | CHD4 | chromodomain helicase DNA binding protein 4 | AI761771 | -2.45 |
| **201276_at** | RAB5B | RAB5B, member RAS oncogene family | AF267863 | -2.46 |
| **221924_at** | DKFZp761I2123 | hypothetical protein DKFZp761I2123 | AW444969 | -2.46 |
| **204132_s_at** | FOXO3A | forkhead box O3A | NM_001455 | -2.46 |
| **210719_s_at** | HMG20B | high-mobility group 20B | BC002552 | -2.46 |
| **226663_at** | ANKRD10 | ankyrin repeat domain 10 | BE670056 | -2.46 |
| **201235_s_at** | BTG2 | BTG family, member 2 | BG339064 | -2.46 |
| **228918_at** | --- | CDNA FLJ32207 fis, clone PLACE6003204 | AI457453 | -2.47 |
| **205090_s_at** | NAGPA | N-acetylglucosamine-1-phosphodiester alpha-N-acetylglucosaminidase | NM_016256 | -2.47 |
| **202860_at** | KIAA0476 | KIAA0476 | NM_014856 | -2.47 |
| **211716_x_at** | ARHGDIA | Rho GDP dissociation inhibitor (GDI) alpha /// Rho GDP dissociation inhibitor (GDI) alpha | BC005851 | -2.47 |
| **213839_at** | KIAA0500 | KIAA0500 protein | AW028110 | -2.47 |
| **202492_at** | ABCB6 /// ATG9A | ATP-binding cassette, sub-family B (MDR/TAP), member 6 /// ATG9 autophagy related 9 homolog A (S. cerevisiae) | NM_024085 | -2.47 |
| **213318_s_at** | BAT3 | HLA-B associated transcript 3 | BG028844 | -2.48 |
| **227237_x_at** | ATAD3B | ATPase family, AAA domain containing 3B | AW593303 | -2.48 |
| **202102_s_at** | BRD4 | bromodomain containing 4 | BF718610 | -2.48 |
| **221664_s_at** | F11R | F11 receptor | AF154005 | -2.49 |
| **238638_at** | SLC37A2 | solute carrier family 37 (glycerol-3-phosphate transporter), member 2 | AI935644 | -2.49 |
| **57715_at** | FAM26B | family with sequence similarity 26, member B | W72694 | -2.50 |
| **203737_s_at** | PPRC1 | peroxisome proliferative activated receptor, gamma, coactivator-related 1 | NM_015062 | -2.50 |
| **239213_at** | SERPINB1 | Serpin peptidase inhibitor, clade B (ovalbumin), member 1 | W52010 | -2.50 |
| **212028_at** | RBM25 | RNA binding motif protein 25 | BE466128 | -2.50 |
| **225135_at** | SIN3A | SIN3 homolog A, transcription regulator (yeast) | AI433017 | -2.50 |
| **215684_s_at** | ASCC2 | activating signal cointegrator 1 complex subunit 2 | AL096741 | -2.51 |
| **221565_s_at** | FAM26B | family with sequence similarity 26, member B | BC000039 | -2.51 |
| **217899_at** | FLJ20254 | Hypothetical protein FLJ20254 | NM_017727 | -2.51 |
| **225115_at** | HIPK2 | Homeodomain interacting protein kinase 2 | BF529628 | -2.51 |
| **204998_s_at** | ATF5 | activating transcription factor 5 | NM_012068 | -2.52 |
| **202264_s_at** | TOMM40 | translocase of outer mitochondrial membrane 40 homolog (yeast) | NM_006114 | -2.52 |
| **227972_at** | TOR2A | torsin family 2, member A | AA873275 | -2.52 |
| **213733_at** | MYO1F | myosin IF | BF740152 | -2.53 |
| **230742_at** | --- | --- | AA742596 | -2.53 |
| **202570_s_at** | DLGAP4 | discs, large (Drosophila) homolog-associated protein 4 | BF346592 | -2.53 |
| **207801_s_at** | RNF10 | ring finger protein 10 | NM_014868 | -2.53 |
| **212198_s_at** | TM9SF4 | transmembrane 9 superfamily protein member 4 | AL515964 | -2.54 |
| **209333_at** | ULK1 | unc-51-like kinase 1 (C. elegans) | AB018265 | -2.55 |
| **221267_s_at** | C19orf27 | chromosome 19 open reading frame 27 /// chromosome 19 open reading frame 27 | NM_031213 | -2.55 |
| **207697_x_at** | LILRB2 | leukocyte immunoglobulin-like receptor, subfamily B (with TM and ITIM domains), member 2 | NM_005874 | -2.55 |
| **204206_at** | MNT | MAX binding protein | NM_020310 | -2.55 |
| **201683_x_at** | C14orf92 | chromosome 14 open reading frame 92 | BE783632 | -2.55 |
| **226310_at** | AVO3 | TORC2-specific protein AVO3 | AI743511 | -2.55 |
| **222694_at** | MGC2752 | hypothetical protein MGC2752 | AI015612 | -2.55 |
| **224846_at** | SHKBP1 | SH3KBP1 binding protein 1 | AI348000 | -2.56 |
| **1557081_at** | RBM25 | RNA binding motif protein 25 | AA580691 | -2.56 |
| **226531_at** | FLJ14466 | hypothetical protein FLJ14466 | AL530596 | -2.56 |
| **213045_at** | MAST3 | microtubule associated serine/threonine kinase 3 | AB011133 | -2.56 |
| **43544_at** | THRAP5 | thyroid hormone receptor associated protein 5 | AA314406 | -2.57 |
| **227970_at** | GPR157 | G protein-coupled receptor 157 | AK026883 | -2.58 |
| **226640_at** | LOC221955 | KCCR13L | AA015606 | -2.58 |
| **208322_s_at** | ST3GAL1 | ST3 beta-galactoside alpha-2,3-sialyltransferase 1 | NM_003033 | -2.58 |
| **225157_at** | MONDOA | Mlx interactor | AW245631 | -2.58 |
| **204336_s_at** | RGS19 | regulator of G-protein signalling 19 | NM_005873 | -2.59 |
| **202151_s_at** | UBADC1 | ubiquitin associated domain containing 1 | NM_016172 | -2.59 |
| **210172_at** | SF1 | splicing factor 1 | D26121 | -2.59 |
| **227698_s_at** | RAB40C | RAB40C, member RAS oncogene family | AW007215 | -2.59 |
| **233841_s_at** | SDS3 | SDS3 protein | AK026749 | -2.59 |
| **222565_s_at** | PRKD3 | protein kinase D3 | BF978541 | -2.59 |
| **222729_at** | FBXW7 | F-box and WD-40 domain protein 7 (archipelago homolog, Drosophila) | BE551877 | -2.59 |
| **33148_at** | ZFR | zinc finger RNA binding protein | AI459274 | -2.60 |
| **225753_at** | ZNF513 | zinc finger protein 513 | AW003280 | -2.60 |
| **222165_x_at** | C9orf16 | chromosome 9 open reading frame 16 | AK022885 | -2.61 |
| **203419_at** | MLL4 | myeloid/lymphoid or mixed-lineage leukemia 4 | NM_014727 | -2.61 |
| **230422_at** | FPRL2 | formyl peptide receptor-like 2 | AW026543 | -2.61 |
| **242732_at** | NDUFB9 | NADH dehydrogenase (ubiquinone) 1 beta subcomplex, 9, 22kDa | BG010493 | -2.62 |
| **57163_at** | ELOVL1 | elongation of very long chain fatty acids (FEN1/Elo2, SUR4/Elo3, yeast)-like 1 | H93026 | -2.62 |
| **210282_at** | ZNF198 | zinc finger protein 198 | AL136621 | -2.62 |
| **225116_at** | HIPK2 | Homeodomain interacting protein kinase 2 | AW300045 | -2.62 |
| **212860_at** | ZDHHC18 | zinc finger, DHHC-type containing 18 | BG168720 | -2.63 |
| **207522_s_at** | ATP2A3 | ATPase, Ca++ transporting, ubiquitous | NM_005173 | -2.63 |
| **219878_s_at** | KLF13 | Kruppel-like factor 13 | NM_015995 | -2.63 |
| **226053_at** | MAP2K7 | mitogen-activated protein kinase kinase 7 | AI090153 | -2.63 |
| **1569150_x_at** | PDLIM7 | PDZ and LIM domain 7 (enigma) | BC023629 | -2.64 |
| **208685_x_at** | BRD2 | bromodomain containing 2 | AA902767 | -2.64 |
| **224726_at** | MIB1 | mindbomb homolog 1 (Drosophila) | W80418 | -2.64 |
| **213763_at** | HIPK2 | Homeodomain interacting protein kinase 2 | R37104 | -2.65 |
| **1552275_s_at** | PXK | PX domain containing serine/threonine kinase | BG573647 | -2.65 |
| **210778_s_at** | MXD4 | MAX dimerization protein 4 | BC002713 | -2.66 |
| **219457_s_at** | RIN3 | Ras and Rab interactor 3 | NM_024832 | -2.66 |
| **208610_s_at** | SRRM2 | serine/arginine repetitive matrix 2 | AI655799 | -2.66 |
| **228762_at** | LFNG | lunatic fringe homolog (Drosophila) | AW151924 | -2.66 |
| **223281_s_at** | COX15 | COX15 homolog, cytochrome c oxidase assembly protein (yeast) | AF026850 | -2.66 |
| **203490_at** | ELF4 | E74-like factor 4 (ets domain transcription factor) | NM_001421 | -2.67 |
| **1570511_at** | ARHGEF10L | Rho guanine nucleotide exchange factor (GEF) 10-like | BC029928 | -2.67 |
| **225935_at** | --- | CDNA clone IMAGE:4769453 | AI350995 | -2.67 |
| **219988_s_at** | C1orf164 | chromosome 1 open reading frame 164 | NM_018150 | -2.67 |
| **223113_at** | HSPC196 | hypothetical protein HSPC196 | AF151030 | -2.68 |
| **212360_at** | AMPD2 | adenosine monophosphate deaminase 2 (isoform L) | AI916249 | -2.68 |
| **224904_at** | PDPR | pyruvate dehydrogenase phosphatase regulatory subunit | AV724415 | -2.68 |
| **218376_s_at** | MICAL1 | microtubule associated monoxygenase, calponin and LIM domain containing 1 | NM_022765 | -2.68 |
| **200990_at** | TRIM28 | tripartite motif-containing 28 | NM_005762 | -2.69 |
| **228801_at** | ORMDL1 | ORM1-like 1 (S. cerevisiae) | AI809749 | -2.69 |
| **1555913_at** | GON4 | gon-4 homolog (C.elegans) | AK098734 | -2.70 |
| **201602_s_at** | PPP1R12A | protein phosphatase 1, regulatory (inhibitor) subunit 12A | BE737620 | -2.71 |
| **211342_x_at** | MED12 | mediator of RNA polymerase II transcription, subunit 12 homolog (yeast) | BC004354 | -2.71 |
| **227069_at** | --- | CDNA clone IMAGE:4769453 | AA806989 | -2.72 |
| **221732_at** | CANT1 | calcium activated nucleotidase 1 | AK026161 | -2.72 |
| **224908_s_at** | TTL | tubulin tyrosine ligase | AI888594 | -2.72 |
| **209864_at** | FRAT2 | frequently rearranged in advanced T-cell lymphomas 2 | AB045118 | -2.72 |
| **223553_s_at** | DOK3 | docking protein 3 | BC004564 | -2.72 |
| **204153_s_at** | MFNG | manic fringe homolog (Drosophila) | NM_002405 | -2.72 |
| **205823_at** | RGS12 | regulator of G-protein signalling 12 | AI824113 | -2.72 |
| **202331_at** | BCKDHA | branched chain keto acid dehydrogenase E1, alpha polypeptide (maple syrup urine disease) | NM_000709 | -2.74 |
| **218509_at** | LPPR2 | lipid phosphate phosphatase-related protein type 2 | NM_022737 | -2.74 |
| **212431_at** | KIAA0194 | KIAA0194 protein | D83778 | -2.74 |
| **204198_s_at** | RUNX3 | runt-related transcription factor 3 | AA541630 | -2.75 |
| **91703_at** | EHBP1L1 | EH domain binding protein 1-like 1 | AA149545 | -2.76 |
| **226783_at** | MGC15875 | hypothetical protein MGC15875 | AI762154 | -2.76 |
| **210428_s_at** | HGS | hepatocyte growth factor-regulated tyrosine kinase substrate | AF260566 | -2.76 |
| **212451_at** | KIAA0256 | KIAA0256 gene product | N52532 | -2.76 |
| **229164_s_at** | ABTB1 | ankyrin repeat and BTB (POZ) domain containing 1 | AI337369 | -2.76 |
| **208890_s_at** | PLXNB2 | plexin B2 | BC004542 | -2.76 |
| **221748_s_at** | TNS1 | tensin 1 /// tensin 1 | AL046979 | -2.77 |
| **209895_at** | PTPN11 | protein tyrosine phosphatase, non-receptor type 11 (Noonan syndrome 1) | AF119855 | -2.77 |
| **1554251_at** | HP1BP3 | heterochromatin protein 1, binding protein 3 | BC022342 | -2.77 |
| **239486_at** | --- | Transcribed locus | BG111636 | -2.77 |
| **202082_s_at** | SEC14L1 | SEC14-like 1 (S. cerevisiae) | AV748469 | -2.77 |
| **227514_at** | LOC162073 | Hypothetical protein LOC162073 | AI766311 | -2.78 |
| **222482_at** | SSBP3 | single stranded DNA binding protein 3 | AA102468 | -2.78 |
| **223152_at** | PPP1R12C | protein phosphatase 1, regulatory (inhibitor) subunit 12C | AF312028 | -2.78 |
| **204538_x_at** | NPIP /// LOC339047 /// LOC440341 | nuclear pore complex interacting protein /// hypothetical protein LOC339047 /// similar to hypothetical protein LOC339047 | NM_006985 | -2.78 |
| **1569149_at** | PDLIM7 | PDZ and LIM domain 7 (enigma) | BC023629 | -2.78 |
| **202153_s_at** | NUP62 | nucleoporin 62kDa | NM_016553 | -2.78 |
| **202116_at** | DPF2 | D4, zinc and double PHD fingers family 2 | NM_006268 | -2.79 |
| **203085_s_at** | TGFB1 | transforming growth factor, beta 1 (Camurati-Engelmann disease) | BC000125 | -2.79 |
| **218938_at** | FBXL15 | F-box and leucine-rich repeat protein 15 | NM_024326 | -2.79 |
| **241701_at** | ARHGAP21 | Rho GTPase activating protein 21 | BF369489 | -2.79 |
| **1554670_at** | GGA1 | golgi associated, gamma adaptin ear containing, ARF binding protein 1 | BC029388 | -2.79 |
| **211993_at** | WNK1 | WNK lysine deficient protein kinase 1 /// WNK lysine deficient protein kinase 1 | AI768512 | -2.79 |
| **208858_s_at** | FAM62A | family with sequence similarity 62 (C2 domain containing), member A | BC004998 | -2.79 |
| **206978_at** | CCR2 | chemokine (C-C motif) receptor 2 /// chemokine (C-C motif) receptor 2 | NM_000647 | -2.80 |
| **212368_at** | ZNF292 | zinc finger protein 292 | AA972711 | -2.80 |
| **200727_s_at** | ACTR2 | ARP2 actin-related protein 2 homolog (yeast) | AA699583 | -2.80 |
| **221501_x_at** | LOC339047 | hypothetical protein LOC339047 | AF229069 | -2.81 |
| **201185_at** | HTRA1 | HtrA serine peptidase 1 | NM_002775 | -2.81 |
| **219558_at** | ATP13A3 | ATPase type 13A3 | NM_024524 | -2.82 |
| **225937_at** | --- | CDNA clone IMAGE:4769453 | BF002121 | -2.82 |
| **241955_at** | HECTD1 | HECT domain containing 1 | BE243270 | -2.82 |
| **213514_s_at** | DIAPH1 | diaphanous homolog 1 (Drosophila) | AU158818 | -2.82 |
| **201945_at** | FURIN | furin (paired basic amino acid cleaving enzyme) | NM_002569 | -2.82 |
| **201715_s_at** | ACIN1 | apoptotic chromatin condensation inducer 1 | NM_014977 | -2.82 |
| **202424_at** | MAP2K2 | mitogen-activated protein kinase kinase 2 | NM_030662 | -2.82 |
| **204546_at** | KIAA0513 | KIAA0513 | NM_014732 | -2.82 |
| **228499_at** | PFKFB4 | 6-phosphofructo-2-kinase/fructose-2,6-biphosphatase 4 | AL038787 | -2.83 |
| **210281_s_at** | ZNF198 | zinc finger protein 198 | AL136621 | -2.83 |
| **201977_s_at** | KIAA0141 | KIAA0141 | AI539425 | -2.83 |
| **200601_at** | ACTN4 | actinin, alpha 4 | U48734 | -2.84 |
| **225364_at** | STK4 | serine/threonine kinase 4 | BE222274 | -2.85 |
| **213286_at** | ZFR | zinc finger RNA binding protein | BF445199 | -2.86 |
| **35436_at** | GOLGA2 | golgi autoantigen, golgin subfamily a, 2 | L06147 | -2.86 |
| **212076_at** | MLL | myeloid/lymphoid or mixed-lineage leukemia (trithorax homolog, Drosophila) | AI701430 | -2.86 |
| **212492_s_at** | JMJD2B | jumonji domain containing 2B | AW237172 | -2.87 |
| **212484_at** | FAM89B | family with sequence similarity 89, member B | BF974389 | -2.87 |
| **1555945_s_at** | C9orf10 | chromosome 9 open reading frame 10 | AK091785 | -2.87 |
| **204166_at** | KIAA0963 | KIAA0963 | NM_014963 | -2.87 |
| **210660_at** | LILRA1 | leukocyte immunoglobulin-like receptor, subfamily A (with TM domain), member 1 /// leukocyte immunoglobulin-like receptor, subfamily A (with TM domain), member 1 | AF025529 | -2.87 |
| **242665_at** | FMNL2 | formin-like 2 | AL042120 | -2.87 |
| **203230_at** | DVL1 | dishevelled, dsh homolog 1 (Drosophila) | AF006011 | -2.88 |
| **225701_at** | AKNA | AT-hook transcription factor | AK024431 | -2.89 |
| **230703_at** | C14orf32 | Chromosome 14 open reading frame 32 | AA001543 | -2.89 |
| **218913_s_at** | GMIP | GEM interacting protein | NM_016573 | -2.89 |
| **215338_s_at** | NKTR | natural killer-tumor recognition sequence | AI688640 | -2.89 |
| **240652_at** | --- | --- | T91029 | -2.89 |
| **201039_s_at** | RAD23A | RAD23 homolog A (S. cerevisiae) | BF572938 | -2.90 |
| **219028_at** | HIPK2 | homeodomain interacting protein kinase 2 | NM_022740 | -2.90 |
| **209136_s_at** | USP10 | ubiquitin specific peptidase 10 | BG390445 | -2.90 |
| **234306_s_at** | SLAMF7 | SLAM family member 7 | AJ271869 | -2.90 |
| **222586_s_at** | OSBPL11 | oxysterol binding protein-like 11 | AI884890 | -2.91 |
| **218812_s_at** | C7orf19 | chromosome 7 open reading frame 19 | NM_025156 | -2.91 |
| **212064_x_at** | MAZ | MYC-associated zinc finger protein (purine-binding transcription factor) | AI471665 | -2.91 |
| **207604_s_at** | SLC4A7 | solute carrier family 4, sodium bicarbonate cotransporter, member 7 | NM_003615 | -2.92 |
| **230180_at** | DDX17 | DEAD (Asp-Glu-Ala-Asp) box polypeptide 17 | AA521056 | -2.92 |
| **1568680_s_at** | YTHDC2 | YTH domain containing 2 | BC019100 | -2.92 |
| **214250_at** | NUMA1 | nuclear mitotic apparatus protein 1 | AI337584 | -2.93 |
| **201224_s_at** | SRRM1 | serine/arginine repetitive matrix 1 | AU147713 | -2.93 |
| **238642_at** | ANKRD13D | ankyrin repeat domain 13 family, member D | AW367571 | -2.93 |
| **242903_at** | IFNGR1 | Interferon gamma receptor 1 | AI458949 | -2.94 |
| **204184_s_at** | ADRBK2 | adrenergic, beta, receptor kinase 2 | NM_005160 | -2.94 |
| **227152_at** | FLJ10652 | hypothetical protein FLJ10652 | AI979334 | -2.95 |
| **201251_at** | PKM2 | pyruvate kinase, muscle | NM_002654 | -2.95 |
| **207167_at** | IGSF2 | immunoglobulin superfamily, member 2 | NM_004258 | -2.95 |
| **1556066_at** | JMJD3 | jumonji domain containing 3 | AI830331 | -2.95 |
| **217234_s_at** | VIL2 | villin 2 (ezrin) | AF199015 | -2.96 |
| **207966_s_at** | GLG1 | golgi apparatus protein 1 | NM_012201 | -2.96 |
| **225440_at** | AGPAT3 | 1-acylglycerol-3-phosphate O-acyltransferase 3 | BE737251 | -2.96 |
| **233167_at** | SELO | selenoprotein O | BC001099 | -2.97 |
| **50221_at** | TFEB | transcription factor EB | AI524138 | -2.97 |
| **1562194_at** | ZFHX1B | Zinc finger homeobox 1b | AF086037 | -2.98 |
| **205205_at** | RELB | v-rel reticuloendotheliosis viral oncogene homolog B, nuclear factor of kappa light polypeptide gene enhancer in B-cells 3 (avian) | NM_006509 | -2.98 |
| **219159_s_at** | SLAMF7 | SLAM family member 7 | NM_021181 | -2.98 |
| **227062_at** | TncRNA | trophoblast-derived noncoding RNA | AU155361 | -2.99 |
| **228343_at** | POU2F2 | POU domain, class 2, transcription factor 2 | AA805754 | -2.99 |
| **207453_s_at** | DNAJB5 | DnaJ (Hsp40) homolog, subfamily B, member 5 | NM_012266 | -2.99 |
| **38157_at** | DOM3Z | dom-3 homolog Z (C. elegans) | AF059252 | -3.00 |
| **201711_x_at** | RANBP2 | RAN binding protein 2 | AI681120 | -3.00 |
| **210218_s_at** | SP100 | nuclear antigen Sp100 | U36501 | -3.00 |
| **213328_at** | NEK1 | NIMA (never in mitosis gene a)-related kinase 1 | AI936517 | -3.01 |
| **220748_s_at** | ZNF580 | zinc finger protein 580 | NM_016202 | -3.01 |
| **203958_s_at** | ZBTB40 | zinc finger and BTB domain containing 40 | AI557467 | -3.02 |
| **203825_at** | BRD3 | bromodomain containing 3 | NM_007371 | -3.03 |
| **218555_at** | ANAPC2 | anaphase promoting complex subunit 2 | NM_013366 | -3.04 |
| **239296_at** | ZFHX1B | Zinc finger homeobox 1b | AI623184 | -3.05 |
| **202030_at** | BCKDK | branched chain ketoacid dehydrogenase kinase | NM_005881 | -3.06 |
| **205025_at** | HKR3 | GLI-Kruppel family member HKR3 | NM_005341 | -3.07 |
| **203514_at** | MAP3K3 | mitogen-activated protein kinase kinase kinase 3 | BF971923 | -3.07 |
| **37384_at** | PPM1F | protein phosphatase 1F (PP2C domain containing) | D86995 | -3.07 |
| **206756_at** | CHST7 | carbohydrate (N-acetylglucosamine 6-O) sulfotransferase 7 | NM_019886 | -3.07 |
| **226572_at** | SOCS7 | Suppressor of cytokine signaling 7 | AL045666 | -3.08 |
| **229937_x_at** | LILRB1 | Leukocyte immunoglobulin-like receptor, subfamily B (with TM and ITIM domains), member 3 | AI681260 | -3.09 |
| **210269_s_at** | DXYS155E | DNA segment on chromosome X and Y (unique) 155 expressed sequence | M99578 | -3.09 |
| **1553993_s_at** | MED25 | mediator of RNA polymerase II transcription, subunit 25 homolog (yeast) | BC024312 | -3.11 |
| **214792_x_at** | VAMP2 | vesicle-associated membrane protein 2 (synaptobrevin 2) | AI955119 | -3.11 |
| **222522_x_at** | MRPS10 | mitochondrial ribosomal protein S10 | BG393476 | -3.11 |
| **224516_s_at** | CXXC5 | CXXC finger 5 /// CXXC finger 5 | BC006428 | -3.11 |
| **219869_s_at** | SLC39A8 | solute carrier family 39 (zinc transporter), member 8 | NM_022154 | -3.11 |
| **215780_s_at** | SET /// LOC389168 | SET translocation (myeloid leukemia-associated) /// similar to SET protein (Phosphatase 2A inhibitor I2PP2A) (I-2PP2A) (Template activating factor I) (TAF-I) (HLA-DR associated protein II) (PHAPII) (Inhibitor of granzyme A-activated DNase) (IGAAD) | Z95126 | -3.12 |
| **220326_s_at** | FLJ10357 | hypothetical protein FLJ10357 | NM_018071 | -3.12 |
| **212107_s_at** | DHX9 | DEAH (Asp-Glu-Ala-His) box polypeptide 9 | BE561014 | -3.13 |
| **212437_at** | CENPB | centromere protein B, 80kDa | AL109804 | -3.13 |
| **202771_at** | FAM38A | family with sequence similarity 38, member A | NM_014745 | -3.14 |
| **201613_s_at** | AP1G2 | adaptor-related protein complex 1, gamma 2 subunit | BC000519 | -3.15 |
| **238468_at** | TNRC6B | trinucleotide repeat containing 6B | AA214704 | -3.16 |
| **227749_at** | --- | Transcribed locus | AI703496 | -3.16 |
| **229776_at** | SLCO3A1 | solute carrier organic anion transporter family, member 3A1 | AW138118 | -3.16 |
| **204860_s_at** | BIRC1 | baculoviral IAP repeat-containing 1 | AI817801 | -3.16 |
| **212890_at** | MGC15523 | hypothetical protein MGC15523 | BF663461 | -3.17 |
| **204923_at** | CXorf9 | chromosome X open reading frame 9 | AL023653 | -3.17 |
| **224247_s_at** | MRPS10 | mitochondrial ribosomal protein S10 | AF277180 | -3.18 |
| **225034_at** | LOC286167 | hypothetical protein LOC286167 | AI885015 | -3.19 |
| **213956_at** | CAP350 | centrosome-associated protein 350 | AW299294 | -3.20 |
| **1558747_at** | SMCHD1 | structural maintenance of chromosomes flexible hinge domain containing 1 | AA336502 | -3.20 |
| **210649_s_at** | ARID1A | AT rich interactive domain 1A (SWI- like) | AF231056 | -3.23 |
| **230392_at** | HNRPM | Heterogeneous nuclear ribonucleoprotein M | AW298141 | -3.24 |
| **222544_s_at** | WHSC1L1 | Wolf-Hirschhorn syndrome candidate 1-like 1 | AI697751 | -3.25 |
| **201140_s_at** | RAB5C | RAB5C, member RAS oncogene family | NM_004583 | -3.25 |
| **212178_s_at** | POM121 /// LOC340318 /// LOC441253 | POM121 membrane glycoprotein (rat) /// nuclear envelope pore membrane LOC340318 /// nuclear envelope pore membrane LOC441253 | AK022555 | -3.25 |
| **224909_s_at** | PREX1 | phosphatidylinositol 3,4,5-trisphosphate-dependent RAC exchanger 1 | BF308645 | -3.25 |
| **214659_x_at** | YLPM1 | YLP motif containing 1 | AC007956 | -3.26 |
| **227741_at** | PTPLB | protein tyrosine phosphatase-like (proline instead of catalytic arginine), member b | AI813654 | -3.27 |
| **236023_at** | CDK9 | cyclin-dependent kinase 9 (CDC2-related kinase) | AI703465 | -3.27 |
| **225757_s_at** | CLMN | calmin (calponin-like, transmembrane) | AU147564 | -3.27 |
| **212032_s_at** | PTOV1 | prostate tumor overexpressed gene 1 | AL046054 | -3.28 |
| **218632_at** | HECTD3 | HECT domain containing 3 | NM_024602 | -3.28 |
| **224943_at** | BTBD7 | BTB (POZ) domain containing 7 | AI580162 | -3.29 |
| **201072_s_at** | SMARCC1 | SWI/SNF related, matrix associated, actin dependent regulator of chromatin, subfamily c, member 1 | AW152160 | -3.31 |
| **203110_at** | PTK2B | PTK2B protein tyrosine kinase 2 beta | U43522 | -3.32 |
| **204789_at** | FMNL1 | formin-like 1 | NM_005892 | -3.32 |
| **200998_s_at** | CKAP4 | cytoskeleton-associated protein 4 | AW029619 | -3.32 |
| **212291_at** | HIPK1 | homeodomain interacting protein kinase 1 | AI393355 | -3.33 |
| **1556601_a_at** | SPATA13 | Spermatogenesis associated 13 | R69077 | -3.34 |
| **221745_at** | WDR68 | WD repeat domain 68 | BE538424 | -3.35 |
| **204999_s_at** | ATF5 | activating transcription factor 5 | BC005174 | -3.35 |
| **212329_at** | SCAP | SREBP cleavage-activating protein | D83782 | -3.36 |
| **1557227_s_at** | TPR | translocated promoter region (to activated MET oncogene) | AW235355 | -3.36 |
| **227195_at** | ZNF503 | zinc finger protein 503 | AA603467 | -3.37 |
| **229272_at** | FNBP4 | Formin binding protein 4 | AI083506 | -3.38 |
| **203940_s_at** | VASH1 | vasohibin 1 | NM_014909 | -3.38 |
| **203826_s_at** | PITPNM1 | phosphatidylinositol transfer protein, membrane-associated 1 | NM_004910 | -3.38 |
| **224576_at** | KIAA1181 | endoplasmic reticulum-golgi intermediate compartment 32 kDa protein | AK000752 | -3.39 |
| **200767_s_at** | C9orf10 | chromosome 9 open reading frame 10 | NM_014612 | -3.42 |
| **205787_x_at** | ZC3H11A | zinc finger CCCH-type containing 11A | AI803216 | -3.44 |
| **35160_at** | LDB1 | LIM domain binding 1 | AF064491 | -3.45 |
| **203063_at** | PPM1F | protein phosphatase 1F (PP2C domain containing) | NM_014634 | -3.48 |
| **236125_at** | DKFZp586I1420 | Hypothetical protein DKFZp586I1420 | AA832073 | -3.48 |
| **1552611_a_at** | JAK1 | Janus kinase 1 (a protein tyrosine kinase) | AL555086 | -3.48 |
| **225918_at** | LOC146346 | hypothetical protein LOC146346 | AI742940 | -3.49 |
| **223179_at** | YPEL3 | yippee-like 3 (Drosophila) | BC005009 | -3.51 |
| **208178_x_at** | TRIO | triple functional domain (PTPRF interacting) | NM_007118 | -3.51 |
| **233955_x_at** | CXXC5 | CXXC finger 5 | AK001782 | -3.53 |
| **209411_s_at** | GGA3 | golgi associated, gamma adaptin ear containing, ARF binding protein 3 | AW008018 | -3.55 |
| **204908_s_at** | BCL3 | B-cell CLL/lymphoma 3 | NM_005178 | -3.57 |
| **223701_s_at** | USP47 | ubiquitin specific peptidase 47 | AF059318 | -3.58 |
| **221747_at** | TNS | Tensin 1 /// Tensin 1 | AL046979 | -3.58 |
| **201809_s_at** | ENG | endoglin (Osler-Rendu-Weber syndrome 1) | NM_000118 | -3.59 |
| **1569385_s_at** | KIAA1546 | KIAA1546 protein | BC019007 | -3.59 |
| **201668_x_at** | MARCKS | myristoylated alanine-rich protein kinase C substrate | AW163148 | -3.60 |
| **214870_x_at** | NPIP /// LOC339047 /// LOC440341 | nuclear pore complex interacting protein /// hypothetical protein LOC339047 /// similar to hypothetical protein LOC339047 | AC002045 | -3.61 |
| **208067_x_at** | UTY | ubiquitously transcribed tetratricopeptide repeat gene, Y-linked | NM_007125 | -3.63 |
| **230707_at** | SORL1 | sortilin-related receptor, L(DLR class) A repeats-containing | AA290609 | -3.63 |
| **216212_s_at** | DKC1 | dyskeratosis congenita 1, dyskerin | AJ010395 | -3.64 |
| **202773_s_at** | SFRS8 | splicing factor, arginine/serine-rich 8 (suppressor-of-white-apricot homolog, Drosophila) | AI023864 | -3.66 |
| **1557749_at** | EHBP1L1 | EH domain binding protein 1-like 1 | AK092750 | -3.69 |
| **205809_s_at** | WASL | Wiskott-Aldrich syndrome-like | BE504979 | -3.70 |
| **213998_s_at** | DDX17 | DEAD (Asp-Glu-Ala-Asp) box polypeptide 17 | AW188131 | -3.74 |
| **202205_at** | VASP | vasodilator-stimulated phosphoprotein | NM_003370 | -3.74 |
| **203123_s_at** | SLC11A2 | solute carrier family 11 (proton-coupled divalent metal ion transporters), member 2 | AU154469 | -3.75 |
| **201635_s_at** | FXR1 | fragile X mental retardation, autosomal homolog 1 | AI990766 | -3.75 |
| **200896_x_at** | HDGF | hepatoma-derived growth factor (high-mobility group protein 1-like) | NM_004494 | -3.76 |
| **214121_x_at** | PDLIM7 | PDZ and LIM domain 7 (enigma) | AA086229 | -3.76 |
| **1555526_a_at** | 06.sept | septin 6 | AF403061 | -3.76 |
| **201728_s_at** | KIAA0100 | KIAA0100 gene product | AA904674 | -3.76 |
| **203370_s_at** | PDLIM7 | PDZ and LIM domain 7 (enigma) | NM_005451 | -3.77 |
| **212220_at** | PSME4 | proteasome (prosome, macropain) activator subunit 4 | AI972268 | -3.79 |
| **200956_s_at** | SSRP1 | structure specific recognition protein 1 | BE795648 | -3.83 |
| **235661_at** | --- | Transcribed locus | T99553 | -3.86 |
| **222996_s_at** | CXXC5 | CXXC finger 5 | BC002490 | -3.86 |
| **201107_s_at** | THBS1 | thrombospondin 1 | AI812030 | -3.87 |
| **208710_s_at** | AP3D1 | adaptor-related protein complex 3, delta 1 subunit | AI424923 | -3.91 |
| **233031_at** | ZFHX1B | zinc finger homeobox 1b | AV739670 | -3.91 |
| **230106_at** | ZXDC | ZXD family zinc finger C | BF447897 | -3.91 |
| **200598_s_at** | TRA1 | tumor rejection antigen (gp96) 1 | AI582238 | -3.93 |
| **202876_s_at** | PBX2 | pre-B-cell leukemia transcription factor 2 | NM_002586 | -3.95 |
| **AFFX-HUMRGE/M10098_M_at** | --- | --- | AFFX-HUMRGE/M10098_M | -3.96 |
| **1567013_at** | NFE2L2 | nuclear factor (erythroid-derived 2)-like 2 | AF323119 | -3.98 |
| **229274_at** | GNAS | GNAS complex locus | AI693143 | -3.98 |
| **214035_x_at** | LOC399491 | LOC399491 protein | AA308853 | -4.06 |
| **218029_at** | FAM65A | family with sequence similarity 65, member A | NM_024519 | -4.07 |
| **238350_at** | FLJ25778 | hypothetical protein FLJ25778 | AW967956 | -4.11 |
| **212620_at** | ZNF609 | zinc finger protein 609 | AW165979 | -4.13 |
| **200607_s_at** | RAD21 | RAD21 homolog (S. pombe) | BG289967 | -4.13 |
| **238365_s_at** | MGC33556 | hypothetical LOC339541 | AI638342 | -4.14 |
| **212332_at** | RBL2 | retinoblastoma-like 2 (p130) | BF110947 | -4.22 |
| **229389_at** | FLJ00012 | ATG16 autophagy related 16-like 2 (S. cerevisiae) | AA741058 | -4.23 |
| **45749_at** | FAM65A | family with sequence similarity 65, member A | AA400206 | -4.24 |
| **208987_s_at** | FBXL11 | F-box and leucine-rich repeat protein 11 | AK024505 | -4.25 |
| **210666_at** | IDS | iduronate 2-sulfatase (Hunter syndrome) | AF050145 | -4.25 |
| **204384_at** | GOLGA2 | golgi autoantigen, golgin subfamily a, 2 | NM_004486 | -4.27 |
| **203141_s_at** | AP3B1 | adaptor-related protein complex 3, beta 1 subunit | AW058575 | -4.29 |
| **214333_x_at** | IDH3G | isocitrate dehydrogenase 3 (NAD+) gamma | U69268 | -4.30 |
| **223916_s_at** | BCOR | BCL6 co-repressor | AF317392 | -4.32 |
| **202111_at** | SLC4A2 | solute carrier family 4, anion exchanger, member 2 (erythrocyte membrane protein band 3-like 1) | NM_003040 | -4.34 |
| **204265_s_at** | GPSM3 | G-protein signalling modulator 3 (AGS3-like, C. elegans) | NM_022107 | -4.36 |
| **209675_s_at** | HNRPUL1 | heterogeneous nuclear ribonucleoprotein U-like 1 | BC004242 | -4.37 |
| **242352_at** | NIPBL | Nipped-B homolog (Drosophila) | AW272262 | -4.45 |
| **212831_at** | EGFL5 | EGF-like-domain, multiple 5 | BF110421 | -4.48 |
| **231799_at** | DKFZP564D166 | putative ankyrin-repeat containing protein | AK021886 | -4.65 |
| **216484_x_at** | HDGF | Hepatoma-derived growth factor (high-mobility group protein 1-like) | L24521 | -4.71 |
| **208151_x_at** | DDX17 | DEAD (Asp-Glu-Ala-Asp) box polypeptide 17 /// DEAD (Asp-Glu-Ala-Asp) box polypeptide 17 | NM_030881 | -4.75 |
| **222562_s_at** | TNKS2 | tankyrase, TRF1-interacting ankyrin-related ADP-ribose polymerase 2 | BF060683 | -4.86 |
| **222387_s_at** | VPS35 | vacuolar protein sorting 35 (yeast) | BG476669 | -4.95 |
| **202838_at** | FUCA1 | fucosidase, alpha-L- 1, tissue | NM_000147 | -5.00 |
| **221572_s_at** | SLC26A6 | solute carrier family 26, member 6 | AF288410 | -5.01 |
| **224775_at** | FLJ10006 | hypothetical protein FLJ10006 | AW451291 | -5.01 |
| **242911_at** | THRAP2 | thyroid hormone receptor associated protein 2 | AW999644 | -5.02 |
| **203104_at** | CSF1R | colony stimulating factor 1 receptor, formerly McDonough feline sarcoma viral (v-fms) oncogene homolog /// colony stimulating factor 1 receptor, formerly McDonough feline sarcoma viral (v-fms) oncogene homolog | NM_005211 | -5.06 |
| **208947_s_at** | RENT1 | regulator of nonsense transcripts 1 | U59323 | -5.08 |
| **234725_s_at** | SEMA4B | sema domain, immunoglobulin domain (Ig), transmembrane domain (TM) and short cytoplasmic domain, (semaphorin) 4B | AK026133 | -5.09 |
| **206028_s_at** | MERTK | c-mer proto-oncogene tyrosine kinase | NM_006343 | -5.18 |
| **224992_s_at** | CMIP | c-Maf-inducing protein | AI363061 | -5.30 |
| **222679_s_at** | DCUN1D1 | DCN1, defective in cullin neddylation 1, domain containing 1 (S. cerevisiae) | AW468880 | -5.31 |
| **1558397_at** | --- | CDNA FLJ34100 fis, clone FCBBF3007597 | BF976693 | -5.34 |
| **201996_s_at** | SPEN | spen homolog, transcriptional regulator (Drosophila) | AL524033 | -5.34 |
| **216222_s_at** | MYO10 | myosin X | AI561354 | -5.52 |
| **205423_at** | AP1B1 | adaptor-related protein complex 1, beta 1 subunit | NM_001127 | -5.67 |
| **207556_s_at** | DGKZ | diacylglycerol kinase, zeta 104kDa | NM_003646 | -5.69 |
| **224962_at** | LOC90120 | hypothetical gene supported by AK023162 | AU153030 | -5.72 |
| **220091_at** | SLC2A6 | solute carrier family 2 (facilitated glucose transporter), member 6 | NM_017585 | -5.79 |
| **225377_at** | C9orf86 | chromosome 9 open reading frame 86 | BE783949 | -6.39 |
| **200917_s_at** | SRPR | signal recognition particle receptor ('docking protein') | BG474541 | -6.42 |
| **204270_at** | SKI | v-ski sarcoma viral oncogene homolog (avian) | AI568728 | -6.50 |
| **236495_at** | PBEF1 | Pre-B-cell colony enhancing factor 1 | AI681868 | -6.59 |
| **201373_at** | PLEC1 | plectin 1, intermediate filament binding protein 500kDa | NM_000445 | -6.64 |
| **218051_s_at** | FLJ12442 | hypothetical protein FLJ12442 | NM_022908 | -6.80 |
| **240773_at** | TRIO | Triple functional domain (PTPRF interacting) | AW449903 | -6.93 |
| **1557910_at** | HSPCB | heat shock 90kDa protein 1, beta | BG612458 | -7.21 |
| **216361_s_at** | MYST3 | MYST histone acetyltransferase (monocytic leukemia) 3 | AJ251844 | -7.33 |
| **216392_s_at** | SEC23IP | SEC23 interacting protein | AK021846 | -8.49 |
| **208879_x_at** | C20orf14 | chromosome 20 open reading frame 14 | BG469030 | -9.55 |
| **224563_at** | WASF2 | WAS protein family, member 2 | BG338758 | -9.92 |
| **222527_s_at** | RBM22 | RNA binding motif protein 22 | AL538762 | -10.78 |
| **215775_at** | THBS1 | Thrombospondin 1 | BF084105 | -13.55 |
| **239336_at** | THBS1 | Thrombospondin 1 | BF109732 | -15.54 |
