# Supplementary material for: Blockade of T Cell Contact-Activation of Human Monocytes by High-Density Lipoproteins Reveals a New Pattern of Cytokine and Inflammatory Genes
Source: PLoS One. 2010 Feb 25;5(2):e9418. doi: 10.1371/journal.pone.0009418 (PMC2828473; doi:10.1371/journal.pone.0009418)
Supplement: Table S3 — Probe sets whose signal was modulated by HDL. (0.15 MB DOC) [file pone.0009418.s003.doc]

# Table S3: Probe sets whose signal was modulated by HDL

| **Affymetrix ID** | **Gene Symbol** | **Description** | **GeneBank ID** | **Fold Change*** |
| --- | --- | --- | --- | --- |
| **AFFX-r2-Bs-lys-3_at** | --- | --- | AFFX-r2-Bs-lys-3 | 12.04 |
| **AFFX-LysX-3_at** | --- | --- | AFFX-LysX-3 | 10.11 |
| **225207_at** | PDK4 | pyruvate dehydrogenase kinase, isoenzyme 4 | AV707102 | 7.49 |
| **203665_at** | HMOX1 | heme oxygenase (decycling) 1 | NM_002133 | 6.62 |
| **AFFX-r2-Bs-phe-3_at** | --- | --- | AFFX-r2-Bs-phe-3 | 6.06 |
| **228325_at** | KIAA0146 | KIAA0146 protein | AI363213 | 5.33 |
| **213006_at** | CEBPD | CCAAT/enhancer binding protein (C/EBP), delta | AV655640 | 4.59 |
| **236140_at** | GCLM | glutamate-cysteine ligase, modifier subunit | AI753488 | 4.44 |
| **214020_x_at** | ITGB5 | Integrin, beta 5 | BE138575 | 4.39 |
| **228186_s_at** | RSPO3 | R-spondin 3 homolog (Xenopus laevis) | BF589322 | 4.32 |
| **205333_s_at** | RCE1 | RCE1 homolog, prenyl protein peptidase (S. cerevisiae) | NM_005133 | 4.06 |
| **208808_s_at** | HMGB2 | high-mobility group box 2 | BC000903 | 3.33 |
| **242280_x_at** | CPEB4 | Cytoplasmic polyadenylation element binding protein 4 | N53564 | 3.29 |
| **201354_s_at** | BAZ2A | bromodomain adjacent to zinc finger domain, 2A | AA788652 | 3.23 |
| **203973_s_at** | CEBPD | CCAAT/enhancer binding protein (C/EBP), delta | NM_005195 | 3.15 |
| **211653_x_at** | AKR1C2 | aldo-keto reductase family 1, member C2 (dihydrodiol dehydrogenase 2; bile acid binding protein; 3-alpha hydroxysteroid dehydrogenase, type III) /// aldo-keto reductase family 1, member C2 (dihydrodiol dehydrogenase 2; bile acid binding protein; 3-alpha hydroxysteroid dehydrogenase, type III) | M33376 | 2.98 |
| **234986_at** | GCLM | Glutamate-cysteine ligase, modifier subunit | AA630626 | 2.91 |
| **205770_at** | GSR | glutathione reductase | NM_000637 | 2.89 |
| **203925_at** | GCLM | glutamate-cysteine ligase, modifier subunit | NM_002061 | 2.87 |
| **231897_at** | LTB4DH | leukotriene B4 12-hydroxydehydrogenase | AL135787 | 2.85 |
| **219475_at** | OKL38 | pregnancy-induced growth inhibitor | NM_013370 | 2.74 |
| **219132_at** | PELI2 | pellino homolog 2 (Drosophila) | NM_021255 | 2.73 |
| **201466_s_at** | JUN | v-jun sarcoma virus 17 oncogene homolog (avian) | NM_002228 | 2.71 |
| **201369_s_at** | ZFP36L2 | zinc finger protein 36, C3H type-like 2 | NM_006887 | 2.69 |
| **225451_at** | GRIPAP1 | GRIP1 associated protein 1 | AL136847 | 2.66 |
| **1565162_s_at** | MGST1 | microsomal glutathione S-transferase 1 | D16947 | 2.62 |
| **222258_s_at** | SH3BP4 | SH3-domain binding protein 4 | AF015043 | 2.61 |
| **204151_x_at** | AKR1C1 | aldo-keto reductase family 1, member C1 (dihydrodiol dehydrogenase 1; 20-alpha (3-alpha)-hydroxysteroid dehydrogenase) | NM_001353 | 2.58 |
| **204759_at** | RCBTB2 | regulator of chromosome condensation (RCC1) and BTB (POZ) domain containing protein 2 | NM_001268 | 2.56 |
| **216594_x_at** | AKR1C1 | aldo-keto reductase family 1, member C1 (dihydrodiol dehydrogenase 1; 20-alpha (3-alpha)-hydroxysteroid dehydrogenase) | S68290 | 2.52 |
| **206662_at** | GLRX | glutaredoxin (thioltransferase) | NM_002064 | 2.51 |
| **209160_at** | AKR1C3 | aldo-keto reductase family 1, member C3 (3-alpha hydroxysteroid dehydrogenase, type II) | AB018580 | 2.46 |
| **210519_s_at** | NQO1 | NAD(P)H dehydrogenase, quinone 1 | BC000906 | 2.42 |
| **203427_at** | ASF1A | ASF1 anti-silencing function 1 homolog A (S. cerevisiae) | NM_014034 | 2.35 |
| **236488_s_at** | --- | CDNA FLJ36309 fis, clone THYMU2004986 | AI683805 | 2.26 |
| **228120_at** | --- | CDNA: FLJ22073 fis, clone HEP11868 | AW136032 | 2.22 |
| **222378_at** | FLJ43663 | Hypothetical protein FLJ43663 | AW973791 | 2.17 |
| **242735_x_at** | --- | Transcribed locus | T85294 | 2.10 |
| **210361_s_at** | ELF2 | E74-like factor 2 (ets domain transcription factor) | AF256223 | 2.10 |
| **213524_s_at** | G0S2 | G0/G1switch 2 | NM_015714 | -2.06 |
| **1569003_at** | TMEM49 | transmembrane protein 49 | AL541655 | -2.07 |
| **213746_s_at** | FLNA | filamin A, alpha (actin binding protein 280) | AW051856 | -2.08 |
| **1552423_at** | ETV3 | ets variant gene 3 | NM_005240 | -2.09 |
| **222703_s_at** | YRDC | yrdC domain containing (E.coli) | BE464161 | -2.12 |
| **232150_at** | C20orf18 | Chromosome 20 open reading frame 18 | AA134418 | -2.13 |
| **1556744_a_at** | LOC440971 | similar to Zinc finger protein Rlf (Rearranged L-myc fusion gene protein) (Zn-15 related protein) | AI732587 | -2.19 |
| **213624_at** | SMPDL3A | sphingomyelin phosphodiesterase, acid-like 3A | AA873600 | -2.20 |
| **228170_at** | OLIG1 | oligodendrocyte transcription factor 1 | AL355743 | -2.24 |
| **211367_s_at** | CASP1 | caspase 1, apoptosis-related cysteine peptidase (interleukin 1, beta, convertase) | U13699 | -2.24 |
| **222669_s_at** | SBDS | Shwachman-Bodian-Diamond syndrome | AK001779 | -2.26 |
| **1553785_at** | RASGEF1B | RasGEF domain family, member 1B | NM_152545 | -2.27 |
| **44790_s_at** | C13orf18 | chromosome 13 open reading frame 18 | AI129310 | -2.32 |
| **1569409_x_at** | INHA | Inhibin, alpha | BC037812 | -2.34 |
| **201127_s_at** | ACLY | ATP citrate lyase | AI971281 | -2.35 |
| **219648_at** | DSU | dilute suppressor | NM_018000 | -2.44 |
| **219736_at** | TRIM36 | tripartite motif-containing 36 | NM_018700 | -2.48 |
| **217299_s_at** | NBN | nibrin | AK001017 | -2.49 |
| **240232_at** | C3orf1 | Chromosome 3 open reading frame 1 | AA503803 | -2.49 |
| **202909_at** | EPM2AIP1 | EPM2A (laforin) interacting protein 1 | NM_014805 | -2.53 |
| **238729_x_at** | SAV1 | Salvador homolog 1 (Drosophila) | BF983202 | -2.62 |
| **227847_at** | EPM2AIP1 | EPM2A (laforin) interacting protein 1 | BF432224 | -2.69 |
| **242029_at** | FNDC3B | Fibronectin type III domain containing 3B | N32832 | -2.70 |
| **214333_x_at** | IDH3G | isocitrate dehydrogenase 3 (NAD+) gamma | U69268 | -2.70 |
| **219471_at** | C13orf18 | chromosome 13 open reading frame 18 | NM_025113 | -2.71 |
| **1554519_at** | CD80 | CD80 antigen (CD28 antigen ligand 1, B7-1 antigen) | BC042665 | -2.73 |
| **205327_s_at** | ACVR2A | activin A receptor, type IIA | NM_001616 | -2.78 |
| **219869_s_at** | SLC39A8 | solute carrier family 39 (zinc transporter), member 8 | NM_022154 | -2.83 |
| **206157_at** | PTX3 | pentraxin-related gene, rapidly induced by IL-1 beta | NM_002852 | -2.83 |
| **244868_at** | ZHX2 | Zinc fingers and homeoboxes 2 | AA001941 | -2.84 |
| **212659_s_at** | IL1RN | interleukin 1 receptor antagonist | AW083357 | -2.90 |
| **235549_at** | IBRDC2 | IBR domain containing 2 | AL575512 | -2.96 |
| **209267_s_at** | SLC39A8 | solute carrier family 39 (zinc transporter), member 8 | AB040120 | -3.06 |
| **205569_at** | LAMP3 | lysosomal-associated membrane protein 3 | NM_014398 | -3.08 |
| **207536_s_at** | TNFRSF9 | tumor necrosis factor receptor superfamily, member 9 | NM_001561 | -3.18 |
| **229242_at** | --- | Transcribed locus | BF439063 | -3.20 |
| **225342_at** | AK3L1 | adenylate kinase 3-like 1 | AK026966 | -3.23 |
| **201946_s_at** | CCT2 | chaperonin containing TCP1, subunit 2 (beta) | AL545982 | -3.25 |
| **229437_at** | BIC | BIC transcript | BG231961 | -3.25 |
| **216598_s_at** | CCL2 | chemokine (C-C motif) ligand 2 | S69738 | -3.55 |
| **216061_x_at** | PDGFB | platelet-derived growth factor beta polypeptide (simian sarcoma viral (v-sis) oncogene homolog) | AU150748 | -3.59 |
| **204103_at** | CCL4 | chemokine (C-C motif) ligand 4 | NM_002984 | -3.71 |
| **205114_s_at** | CCL3 /// CCL3L1 /// CCL3L3 | chemokine (C-C motif) ligand 3 /// chemokine (C-C motif) ligand 3-like 1 /// chemokine (C-C motif) ligand 3-like 3 | NM_002983 | -3.72 |
| **204348_s_at** | AK3L1 | adenylate kinase 3-like 1 | NM_013410 | -3.76 |
| **1562749_at** | --- | CDNA FLJ27026 fis, clone SLV07378 | BC040865 | -3.92 |
| **210118_s_at** | IL1A | interleukin 1, alpha | M15329 | -4.01 |
| **225987_at** | STEAP4 | STEAP family member 4 | AA650281 | -4.21 |
| **240287_at** | LOC341720 | similar to immune-responsive gene 1 | BG236136 | -4.31 |
| **233811_at** | RIN2 | Ras and Rab interactor 2 | AK026753 | -4.74 |
| **231513_at** | KCNJ2 | Potassium inwardly-rectifying channel, subfamily J, member 2 | BF111326 | -5.20 |
| **205476_at** | CCL20 | chemokine (C-C motif) ligand 20 | NM_004591 | -5.25 |
| **239012_at** | IBRDC2 | IBR domain containing 2 | R83905 | -6.54 |
| **206765_at** | KCNJ2 | potassium inwardly-rectifying channel, subfamily J, member 2 | AF153820 | -6.62 |
