# Supplementary material for: Blockade of T Cell Contact-Activation of Human Monocytes by High-Density Lipoproteins Reveals a New Pattern of Cytokine and Inflammatory Genes
Source: PLoS One. 2010 Feb 25;5(2):e9418. doi: 10.1371/journal.pone.0009418 (PMC2828473; doi:10.1371/journal.pone.0009418)
Supplement: Table S4 — Probe sets whose signals was lower in monocytes activated by CEsHUT + HDL than in monocytes activated by CEsHUT. (0.29 MB DOC) [file pone.0009418.s004.doc]

# Table S4: Probe sets whose signals was lower in monocytes activated by CE_sHUT_ + HDL than in monocytes activated by CE_sHUT_

| **Affymetrix ID** | **Gene Symbol** | **Description** | **Representative ID** | **fold change** |
| --- | --- | --- | --- | --- |
| 200808_s_at | ZYX | zyxin | NM_003461 | -5.19 |
| 209716_at | CSF1 | Macrophage colony-stimulating factor | M37435 | -5.19 |
| 202768_at | FOSB | Protein fosB (G0/G1 switch regulatory protein 3) | NM_006732 | -4.42 |
| 41386_i_at | JMJD3 | Lysine-specific demethylase 6B | AB002344 | -4.40 |
| 209325_s_at | RGS16 | regulator of G-protein signalling 16 | U94829 | -4.36 |
| 212659_s_at | IL1RN | interleukin 1 receptor antagonist | AW083357 | -4.30 |
| 244423_at | C10orf45 | Chromosome 10 open reading frame 45 | R99268 | -4.09 |
| 209324_s_at | RGS16 | regulator of G-protein signalling 16 | BF304996 | -3.91 |
| 201693_s_at | EGR1 | early growth response 1 | AV733950 | -3.81 |
| 222802_at | EDN1 | endothelin 1 | J05008 | -3.80 |
| 226201_at | DOT1L | Histone-lysine N-methyltransferase, H3 lysine-79 specific | AI224128 | -3.78 |
| 206359_at | SOCS3 | suppressor of cytokine signaling 3 | BG035761 | -3.56 |
| 206374_at | DUSP8 | dual specificity phosphatase 8 | NM_004420 | -3.50 |
| 212762_s_at | TCF7L2 | transcription factor 7-like 2 (T-cell specific, HMG-box) | AI375916 | -3.44 |
| 229242_at | --- | Transcribed locus | BF439063 | -3.21 |
| 220054_at | IL23A | interleukin 23, alpha subunit p19 | NM_016584 | -3.14 |
| 206267_s_at | MATK | megakaryocyte-associated tyrosine kinase | NM_002378 | -3.09 |
| 235042_at | CMYA1 | Xin actin-binding repeat-containing protein 1 | AW755250 | -3.08 |
| 219150_s_at | ADAP1//CENTA1 | Arf-GAP with dual PH domain-containing protein 1 (centaurin, alpha 1) | NM_006869 | -3.02 |
| 231297_at | DOT1L | Histone-lysine N-methyltransferase, H3 lysine-79 specific | AI479899 | -2.98 |
| 216243_s_at | IL1RN | interleukin 1 receptor antagonist | BE563442 | -2.91 |
| 244035_at | BCL2 | B-cell CLL/lymphoma 2 | BF003032 | -2.89 |
| 202340_x_at | NR4A1 | nuclear receptor subfamily 4, group A, member 1 | NM_002135 | -2.85 |
| 209039_x_at | EHD1 | EH-domain containing 1 | AF001434 | -2.82 |
| 201242_s_at | ATP1B1 | Sodium/potassium-transporting ATPase subunit beta-1 | BC000006 | -2.82 |
| 213338_at | RIS1 // TREM158 | Ras-induced senescence 1 (transmembrane protein 158) | BF062629 | -2.77 |
| 227404_s_at | EGR1 | Early growth response 1 | AI459194 | -2.73 |
| 205476_at | CCL20 | C-C motif chemokine 20 (Macrophage inflammatory protein 3 α; MIP-3α) | NM_004591 | -2.72 |
| 214349_at | --- | Hypothetical LOC388388 | AV764378 | -2.68 |
| 235249_at | RDH13 | Retinol dehydrogenase 13 (all-trans/9-cis) | AA736589 | -2.67 |
| 90265_at | ADAP1 // CENTA1 | Arf-GAP with dual PH domain-containing protein 1 (centaurin, alpha 1) | AW050627 | -2.66 |
| 1560397_s_at | KLHL6 | kelch-like 6 (Drosophila) | AK097976 | -2.63 |
| 204200_s_at | PDGFB | platelet-derived growth factor beta polypeptide (simian sarcoma viral (v-sis) oncogene homolog) | NM_002608 | -2.51 |
| 218266_s_at | FREQ // NCS1 | Neuronal calcium sensor 1 (NCS-1) // frequenin homolog (Drosophila) | NM_014286 | -2.48 |
| 224920_x_at | MYADM | myeloid-associated differentiation marker | AA909044 | -2.45 |
| 212518_at | PIP5K1C | phosphatidylinositol-4-phosphate 5-kinase, type I, gamma | AB011161 | -2.45 |
| 228303_at | GALNT6 | UDP-N-acetyl-alpha-D-galactosamine:polypeptide N-acetylgalactosaminyltransferase 6 (GalNAc-T6) | AW014155 | -2.38 |
| 222221_x_at | EHD1 | EH-domain containing 1 | AY007161 | -2.34 |
| 213475_s_at | ITGAL | integrin, α L (antigen CD11A (p180), lymphocyte function-associated antigen 1; α polypeptide) | AC002310 | -2.32 |
| 205781_at | C16orf7 | Protein ATP-BL // chromosome 16 open reading frame 7 | NM_004913 | -2.31 |
| 205088_at | MAMLD1 // CXorf6 | mastermind-like domain containing 1 // chromosome X open reading frame 6 | NM_005491 | -2.30 |
| 218451_at | CDCP1 | CUB domain containing protein 1 | NM_022842 | -2.30 |
| 223961_s_at | CISH | cytokine inducible SH2-containing protein (CIS) | D83532 | -2.29 |
| 235122_at | HIVEP3 | Transcription factor HIVEP3 (human immunodeficiency virus type I enhancer binding protein 3) | AI800713 | -2.29 |
| 204103_at | CCL4 | chemokine (C-C motif) ligand 4 (Macrophage inflammatory protein 1-β, (MIP-1β) | NM_002984 | -2.29 |
| 204475_at | MMP1 | matrix metallopeptidase 1 (interstitial collagenase) | NM_002421 | -2.27 |
| 238729_x_at | SAV1 | Protein salvador homolog 1 | BF983202 | -2.26 |
| 220882_at | --- | --- | NM_018612 | -2.25 |
| 207376_at | VENTX | Homeobox protein VENTX | NM_014468 | -2.23 |
| 218695_at | EXOSC4 | Exosc4 protein (Exosome complex exonuclease RRP41) | NM_019037 | -2.21 |
| 1554997_a_at | PTGS2 // COX2 | Cyclooxygenase-2 (COX-2) (prostaglandin-endoperoxide synthase 2 // prostaglandin G/H synthase and cyclooxygenase) | AY151286 | -2.19 |
| 219270_at | CHAC1 | Cation transport regulator-like protein 1 | NM_024111 | -2.18 |
| 209875_s_at | SPP1 | secreted phosphoprotein 1 (osteopontin, bone sialoprotein I, early T-lymphocyte activation 1) | M83248 | -2.18 |
| 204926_at | INHBA | inhibin, βA (activin A, activin AB α polypeptide) | NM_002192 | -2.17 |
| 233824_at | --- | CDNA: FLJ21428 fis, clone COL04203 | AK025081 | -2.17 |
| 204411_at | KIF21B | kinesin family member 21B | NM_017596 | -2.15 |
| 1555962_at | B3GNT7 | UDP-GlcNAc:betaGal beta-1,3-N-acetylglucosaminyltransferase 7 | CA503291 | -2.14 |
| 208893_s_at | DUSP6 | dual specificity phosphatase 6 // (Mitogen-activated protein kinase phosphatase 3) (MAP kinase phosphatase 3) (MKP-3) (Dual specificity protein phosphatase PYST1) | BC005047 | -2.13 |
| 210357_s_at | SMOX | spermine oxidase | BC000669 | -2.12 |
| 242633_x_at | ZNF75A | Zinc finger protein 75a | AA829635 | -2.11 |
| 203888_at | THBD | thrombomodulin | NM_000361 | -2.09 |
| 218145_at | TRIB3 | tribbles homolog 3 (Neuronal cell death-inducible putative kinase) (p65-interacting inhibitor of NF-kappa-B) (SINK) | NM_021158 | -2.07 |
| 239105_at | --- | Transcribed locus | AI681581 | -2.06 |
| 207113_s_at | TNF | tumor necrosis factor (TNF superfamily, member 2) (TNF-α) | NM_000594 | -2.06 |
| 224057_s_at | THAP4 | THAP domain containing 4 | BC000767 | -2.05 |
| 237204_at | DPH1 // DPH2L1 | Candidate tumor suppressor in ovarian cancer 2 | AI681671 | -2.04 |
| AFFX-HUMISGF3A/M97935_MA_at | STAT1 | signal transducer and activator of transcription 1, 91kDa | AFFX-HUMISGF3A/M97935_MA | -2.03 |
| 204011_at | SPRY2 | Protein sprouty homolog 2 | NM_005842 | -2.02 |
| 237718_at | EIF4E | eukaryotic translation initiation factor 4E | AA913840 | -2.02 |
| 203470_s_at | PLEK | pleckstrin | AI433595 | -1.99 |
| 204363_at | F3 | coagulation factor III (thromboplastin, tissue factor) | NM_001993 | -1.98 |
| 207316_at | HAS1 | hyaluronan synthase 1 | NM_001523 | -1.95 |
| 215990_s_at | BCL6 | B-cell CLL/lymphoma 6 (zinc finger protein 51) | S67779 | -1.93 |
| 204852_s_at | PTPN7 | protein tyrosine phosphatase, non-receptor type 7 | NM_002832 | -1.92 |
| 212803_at | NAB2 | NGFI-A binding protein 2 (EGR1 binding protein 2) | BF337329 | -1.92 |
| 214867_at | KIAA0913 | Zinc finger SWIM domain-containing protein KIAA0913 | AL080182 | -1.91 |
| 218951_s_at | PLCXD1 | PI-PLC X domain-containing protein 1 (phosphatidylinositol-specific phospholipase C, X domain containing 1) | NM_018390 | -1.91 |
| 215284_at | SNX9 | Sorting nexin 9 | AF070575 | -1.90 |
| 203887_s_at | THBD | thrombomodulin | NM_000361 | -1.90 |
| 1552711_a_at | CYB5D1 | Cytochrome b5 domain-containing protein 1 | NM_144607 | -1.89 |
| 227530_at | AKAP12 | A kinase (PRKA) anchor protein (gravin) 12 | BF511276 | -1.88 |
| 206100_at | CPM | carboxypeptidase M | NM_001874 | -1.87 |
| 221085_at | TNFSF15 | tumor necrosis factor (ligand) superfamily, member 15 (Vascular endothelial cell growth inhibitor) (TNF ligand-related molecule 1) | NM_005118 | -1.87 |
| 203889_at | SCG5 // SGNE1 | **Neuroendocrine protein 7B2** | NM_003020 | -1.85 |
| 204015_s_at | DUSP4 | dual specificity phosphatase 4 | BC002671 | -1.85 |
| 202905_x_at | NBN | nibrin | AI796269 | -1.85 |
| 204804_at | TRIM21 | 52 kDa Ro protein (Sjoegren syndrome type A antigen) (tripartite motif-containing 21) | NM_003141 | -1.83 |
| 35666_at | SEMA3F | sema domain, immunoglobulin domain (Ig), short basic domain, secreted, (semaphorin) 3F | U38276 | -1.82 |
| 221893_s_at | ADCK2 | aarF domain containing kinase 2 | N32831 | -1.82 |
| 227607_at | STAMBPL1// AMSH-LP | STAM binding protein-like 1 (associated molecule with the SH3 domain of STAM (AMSH) like protein) | AI638611 | -1.82 |
| 222816_s_at | ZCCHC2 | zinc finger, CCHC domain containing 2 | BE676543 | -1.81 |
| 203395_s_at | HES1 | Transcription factor HES-1 | NM_005524 | -1.81 |
| 228648_at | LRG1 | leucine-rich alpha-2-glycoprotein 1 | AA622495 | -1.80 |
| 225557_at | CSRNP1 // AXUD1 | AXIN1 up-regulated 1 | AI091372 | -1.79 |
| 227262_at | HAPLN3 | hyaluronan and proteoglycan link protein 3 | BE348293 | -1.79 |
| 205193_at | MAFF | Transcription factor MafF (V-maf musculoaponeurotic fibrosarcoma oncogene homolog F) (U-Maf) | NM_012323 | -1.77 |
| 231078_at | --- | --- | H69701 | -1.76 |
| 221877_at | --- | CDNA FLJ46713 fis, clone TRACH3016885 | BF508835 | -1.76 |
| 202638_s_at | ICAM1 | intercellular adhesion molecule 1 (CD54), human rhinovirus receptor | NM_000201 | -1.75 |
| 240655_at | ALCAM // MEMD | CD166 antigen (Activated leukocyte cell adhesion molecule) (CD antigen CD166) | BE502785 | -1.74 |
| 227250_at | KREMEN1 | Kringle containing transmembrane protein 1 | BF221745 | -1.73 |
| 235688_s_at | TRAF4 | TNF receptor-associated factor 4 | AI992283 | -1.73 |
| 236439_at | BCL6 | B-cell CLL/lymphoma 6 (zinc finger protein 51) | AI733564 | -1.71 |
| 38037_at | HBEGF | heparin-binding EGF-like growth factor (Diphtheria toxin receptor) | M60278 | -1.71 |
| 210517_s_at | AKAP12 | A kinase (PRKA) anchor protein (gravin) 12 | AB003476 | -1.71 |
| 227099_s_at | LOC387763 | hypothetical LOC387763 | AW276078 | -1.70 |
| 205207_at | IL6 | interleukin 6 (interferon, beta 2) | NM_000600 | -1.70 |
| 214228_x_at | TNFRSF4 | tumor necrosis factor receptor superfamily, member 4 (OX40L receptor) (ACT35 antigen) (TAX transcriptionally-activated glycoprotein 1 receptor) (CD134) | AJ277151 | -1.69 |
| 206868_at | STARD8 | START domain containing 8 | NM_014725 | -1.67 |
| 213457_at | MFHAS1 | malignant fibrous histiocytoma amplified sequence 1 | BF739959 | -1.66 |
| 217997_at | PHLDA1 | pleckstrin homology-like domain, family A, member 1 | AI795908 | -1.65 |
| 229437_at | MIRHG2 // BIC | BIC transcript (microRNA host gene 2)(miR-155)(non-protein coding) | BG231961 | -1.65 |
| 200907_s_at | PALLD // KIAA0992 | Palladin (cytoskeletal associated protein) | AU157932 | -1.65 |
| 216015_s_at | CIAS1 // NLRP3 | NACHT, LRR and PYD domains-containing protein 3 (cold autoinflammatory syndrome 1 protein) | AK027194 | -1.64 |
| 212312_at | BCL2L1 | BCL2-like 1 /// BCL2-like 1 | AL117381 | -1.63 |
| 206932_at | CH25H | cholesterol 25-hydroxylase | NM_003956 | -1.63 |
| 218276_s_at | SAV1 | Protein salvador homolog 1 | NM_021818 | -1.63 |
| 230127_at | --- | Transcribed locus | AW044663 | -1.61 |
| 219386_s_at | SLAMF8 | SLAM family member 8 (B-lymphocyte activator macrophage expressed BCM-like membrane protein) | NM_020125 | -1.61 |
| 202637_s_at | ICAM1 | intercellular adhesion molecule 1 (CD54), human rhinovirus receptor | AI608725 | -1.60 |
| 223377_x_at | CISH | cytokine inducible SH2-containing protein | AF035947 | -1.59 |
| 216061_x_at | PDGFB | platelet-derived growth factor beta polypeptide (simian sarcoma viral (v-sis) oncogene homolog) | AU150748 | -1.59 |
| 210118_s_at | IL1A | interleukin 1α (IL-1α) | M15329 | -1.59 |
| 203140_at | BCL6 | B-cell CLL/lymphoma 6 (zinc finger protein 51) /// B-cell CLL/lymphoma 6 (zinc finger protein 51) | NM_001706 | -1.58 |
| 202760_s_at | AKAP2 /// PALM2-AKAP2 | A-kinase anchor protein 2 (AKAP-2) (Protein kinase A-anchoring protein 2) (PRKA2) (AKAP-KL) (PALM2-AKAP2 protein) | NM_007203 | -1.57 |
| 227529_s_at | AKAP12 | A kinase (PRKA) anchor protein (gravin) 12 | BF511276 | -1.57 |
| 201170_s_at | BHLHB2 | Class E basic helix-loop-helix protein 40 (bHLHe40) (Class B basic helix-loop-helix protein 2) (bHLHB2) (Differentially expressed in chondrocytes protein 1) (DEC1) (Enhancer-of-split and hairy-related protein 2) (SHARP-2) (Stimulated by retinoic acid gene 13 protein) | NM_003670 | -1.57 |
| 237252_at | THBD | thrombomodulin | AW119113 | -1.56 |
| 205114_s_at | CCL3 /// CCL3L1 /// CCL3L3 | C-C motif chemokine 3 (Macrophage inflammatory protein 1-α) (MIP-1α) | NM_002983 | -1.55 |
| 223974_at | MGC11082 | hypothetical protein MGC11082 | BC005130 | -1.55 |
| 225955_at | METRNL | meteorin, glial cell differentiation regulator-like | BG231494 | -1.54 |
| 207091_at | P2RX7 | P2X purinoceptor 7 (P2X7) (ATP receptor) (Purinergic receptor) (P2Z receptor) | NM_002562 | -1.53 |
| 203471_s_at | PLEK | pleckstrin | NM_002664 | -1.53 |
| 239331_at | --- | Transcribed locus | AW954199 | -1.53 |
| 210582_s_at | LIMK2 | LIM domain kinase 2 | AL117466 | -1.53 |
| 222573_s_at | SAV1 | Protein salvador homolog 1 | AI679398 | -1.52 |
| 1555976_s_at | --- | LOC440476 | BU676221 | -1.52 |
| 204014_at | DUSP4 | dual specificity phosphatase 4 | NM_001394 | -1.52 |
| 201661_s_at | ACSL3 | Long-chain-fatty-acid--CoA ligase 3 (EC 6.2.1.3) (Long-chain acyl-CoA synthetase 3) (LACS 3) | NM_004457 | -1.51 |
| 203433_at | MTHFS | 5-formyltetrahydrofolate cyclo-ligase (EC 6.3.3.2) (5,10-methenyl-tetrahydrofolate synthetase) (Methenyl-THF synthetase) (MTHFS) | NM_006441 | -1.51 |
| 228738_at | D2HGD | D-2-hydroxyglutarate dehydrogenase, mitochondrial | AI927858 | -1.50 |
| 38158_at | ESPL1 | Separin (EC 3.4.22.49) (Separase) (Caspase-like protein ESPL1) (Extra spindle poles-like 1 protein) | D79987 | -1.50 |
| 223028_s_at | SNX9 | sorting nexin 9 | BC005022 | -1.50 |
| 228674_s_at | EML4 | echinoderm microtubule associated protein like 4 | AA524507 | -1.50 |
| 229715_at | --- | CDNA FLJ41663 fis, clone FEBRA2027297 | AW006182 | -1.50 |
| 235221_at | CBLN3 | cerebellin 3 precursor | R52665 | -1.49 |
| 206420_at | IGSF6 | immunoglobulin superfamily, member 6 (Protein DORA) | NM_005849 | -1.49 |
| 204401_at | KCNN4 | Intermediate conductance calcium-activated potassium channel protein 4 (SK4) (KCa4) (IKCa1) (IK1) (Putative Gardos channel) | NM_002250 | -1.49 |
| 201465_s_at | JUN | Transcription factor AP-1 (Activator protein 1) (AP1) (Proto-oncogene c-jun) (V-jun avian sarcoma virus 17 oncogene homolog) (p39) | BC002646 | -1.49 |
| 214446_at | ELL2 | elongation factor, RNA polymerase II, 2 | NM_012081 | -1.48 |
| 1555833_a_at | IRGQ | Immunity-related GTPase family Q protein (immunity-related GTPase family, Q) (CDNA FLJ46713 fis, clone TRACH3016885) | AK096168 | -1.48 |
| 236606_at | SAV1 | Protein salvador homolog 1 | N50912 | -1.48 |
| 225924_at | FLJ25371 | Hypothetical protein FLJ25371 | AI478634 | -1.48 |
| 1570007_at | LRRC8C // FAD158 | Leucine rich repeat containing 8 family, member C (Protein AD158) | BC036122 | -1.48 |
| 221223_x_at | CISH | cytokine inducible SH2-containing protein (CIS) | NM_013324 | -1.47 |
| 223027_at | SNX9 | sorting nexin 9 | BF972871 | -1.45 |
| 236898_at | --- | Transcribed locus | AW242604 | -1.44 |
| 226498_at | FLT1 | Vascular endothelial growth factor receptor 1 (VEGFR-1) (EC 2.7.10.1) (Vascular permeability factor receptor) (Tyrosine-protein kinase receptor FLT) (Flt-1) (Tyrosine-protein kinase FRT) (Fms-like tyrosine kinase 1) | AA149648 | -1.44 |
| 216563_at | ANKRD12 | Ankyrin repeat domain 12 (Ankyrin repeat-containing cofactor 2) (GAC-1 protein) | X80821 | -1.44 |
| 44120_at | ADCK2 | aarF domain containing kinase 2 | AI879381 | -1.43 |
| 204089_x_at | MAP3K4 | Mitogen-activated protein kinase kinase kinase 4 (EC 2.7.11.25) (MAPK/ERK kinase kinase 4) (MEK kinase 4) (MEKK 4) (MAP three kinase 1) | NM_006724 | -1.42 |
| 231313_at | FLJ27354 | hypothetical gene supported by AK130864 | AW134984 | -1.42 |
| 210845_s_at | PLAUR | Urokinase plasminogen activator surface receptor (uPAR) (U-PAR) (Monocyte activation antigen Mo3) (CD antigen CD87) | U08839 | -1.41 |
| 223196_s_at | SESN2 | Sestrin-2 (Hi95) | AL136551 | -1.40 |
